# Supplementary material for: Biosynthetic pathway for furanosteroid demethoxyviridin and identification of an unusual pregnane side-chain cleavage
Source: Nat Commun. 2018 May 9;9:1838. doi: 10.1038/s41467-018-04298-2 (PMC5943271; doi:10.1038/s41467-018-04298-2)
Supplement: Supplementary file 1 — Supplementary Information [file 41467_2018_4298_MOESM1_ESM.pdf]

Supplementary Information

Biosynthetic Pathway for Furanosteroid Demethoxyviridin and Identification of an Unusual Pregnane Side-chain Cleavage

Table of contents

Supplementary Figures 6

Supplementary Figure 1. Twelve CYP clusters in the genome of *Nodulisporium* sp. (No. 65-12-7-1). 6

Supplementary Figure 2. Production of demethoxyviridin and its derivative by *Nodulisporium* sp. (No. 65-12-7-1) under different culture conditions. 7

Supplementary Figure 3.Expression analysis of a randomly selected CYP gene from each of the twelve CYP clusters. 8

Supplementary Figure 4. Expression analysis of the three candidate CYP clusters. 9

Supplementary Figure 5. Expression analysis of the genes in cluster V and its adjacent genes by RNA-Seq. 10

Supplementary Figure 6. Determination of the boundary of the biosynthetic gene cluster for demethoxyviridin. 11

Supplementary Figure 7. Two different pregnane side-chain cleavage processes. 12

Supplementary Figure 8. SDS-PAGE analysis for purified recombinant VidF. 13

Supplementary Figure 9. The cytotoxicity of compounds **1**−**14** against breast cancer cell line MCF-7. 14

Supplementary Figure 10. Representative gene clusters containing enzymes for pregnane side-chain cleavage from fungi. 15

Supplementary Figure 11. Key ^{1}H−^{1}H COSY and HMBC correlations. 16

Supplementary Figure 12. Key ROESY or NOESY correlations. 17

Supplementary Figure 13. Experimental ECD spectra of **1**–**4** and **6**–**11**. 18

Supplementary Figure 14. The HRESIMS spectrum of **1**. 19

Supplementary Figure 15. The ^{1}H NMR and ^{13}C NMR spectra of **1**. 20

Supplementary Figure 16. The HRESIMS spectrum of **2**. 21

Supplementary Figure 17. The ^{1}H NMR and ^{13}C NMR spectra of **2**. 22

Supplementary Figure 18. The HSQC and ^{1}H-^{1}H COSY spectra of **2**. 23

Supplementary Figure 19. The HMBC and ROESY spectra of **2**. 24

Supplementary Figure 20. The HRESIMS spectrum of **3**. 25

Supplementary Figure 21. The ^{1}H NMR and ^{13}C NMR spectra of **3**. 26

Supplementary Figure 22. The HSQC and ^{1}H-^{1}H COSY spectra of **3**. 27

Supplementary Figure 23. The HMBC spectrum of **3**. 28

Supplementary Figure 24. The HRESIMS spectrum of **4**. 29

Supplementary Figure 25. The ^{1}H NMR and ^{13}C NMR spectra of **4**. 30
Supplementary Figure 26. The HSQC and ^{1}H-^{1}H COSY spectra of **4**. 31
Supplementary Figure 27. The HMBC and ROESY spectra of **4**. 32
Supplementary Figure 28. The HRESIMS spectrum of **5**. 33
Supplementary Figure 29. The ^{1}H NMR and ^{13}C NMR spectra of **5**. 34
Supplementary Figure 30. The HSQC and ^{1}H-^{1}H COSY spectra of **5**. 35
Supplementary Figure 31. The HMBC and NOESY spectra of **5**. 36
Supplementary Figure 32. The HRESIMS spectrum of **6**. 37
Supplementary Figure 33. The ^{1}H NMR and ^{13}C NMR spectra of **6**. 38
Supplementary Figure 34. The HSQC and ^{1}H-^{1}H COSY spectra of **6**. 39
Supplementary Figure 35. The HMBC and ROESY spectra of **6**. 40
Supplementary Figure 36. The HRESIMS spectrum of **7**. 41
Supplementary Figure 37. The ^{1}H NMR and ^{13}C NMR spectra of **7**. 42
Supplementary Figure 38. The HSQC and ^{1}H-^{1}H COSY spectra of **7**. 43
Supplementary Figure 39. The HMBC and ROESY spectra of **7**. 44
Supplementary Figure 40. The HRESIMS spectrum of **8**. 45
Supplementary Figure 41. The ^{1}H NMR and ^{13}C NMR spectra of **8**. 46
Supplementary Figure 42. The HSQC and ^{1}H-^{1}H COSY spectra of **8**. 47
Supplementary Figure 43. The HMBC and ROESY spectra of **8**. 48
Supplementary Figure 44. The HRESIMS spectrum of **9**. 49
Supplementary Figure 45. The ^{1}H NMR and ^{13}C NMR spectra of **9**. 50
Supplementary Figure 46. The HSQC and ^{1}H-^{1}H COSY spectra of **9**. 51
Supplementary Figure 47. The HMBC and NOESY spectra of **9**. 52
Supplementary Figure 48. The HRESIMS spectrum of **10**. 53
Supplementary Figure 49. The ^{1}H NMR and ^{13}C NMR spectra of **10**. 54
Supplementary Figure 50. The HSQC and ^{1}H-^{1}H COSY spectra of **10**. 55
Supplementary Figure 51. The HMBC and ROESY spectra of **10**. 56
Supplementary Figure 52. The HRESIMS spectrum of **11**. 57
Supplementary Figure 53. The ^{1}H NMR and ^{13}C NMR spectra of **11**. 58
Supplementary Figure 54. The HRESIMS spectrum of **12**. 59

Supplementary Figure 55. The ^{1}H NMR and ^{13}C NMR spectra of **12**. 60

Supplementary Figure 56. The HRESIMS spectrum of **13**. 61

Supplementary Figure 57. The ^{1}H NMR and ^{13}C NMR spectra of **13**. 62

Supplementary Figure 58. The HSQC and ^{1}H-^{1}H COSY spectra of **13**. 63

Supplementary Figure 59. The HMBC and ROESY spectra of **13**. 64

Supplementary Figure 60. The HRESIMS spectrum of **14**. 65

Supplementary Figure 61. The ^{1}H NMR and ^{13}C NMR spectra of **14**. 66

Supplementary Figure 62. The HSQC and ^{1}H-^{1}H COSY spectra of **14**. 67

Supplementary Figure 63. The HMBC and ROESY spectra of **14**. 68

Supplementary Figure 64. The HRESIMS spectrum of **15**. 69

Supplementary Figure 65. The ^{1}H NMR and ^{13}C NMR spectra of **15**. 70

Supplementary Figure 66. The HSQC and ^{1}H-^{1}H COSY spectra of **15**. 71

Supplementary Figure 67. The HMBC and ROESY spectra of **15**. 72

Supplementary Figure 68. The HRESIMS spectrum of **21**. 73

Supplementary Figure 69. The ^{1}H NMR and ^{13}C NMR spectra of **21**. 74

Supplementary Figure 70. The HRESIMS spectrum of **24**. 75

Supplementary Figure 71. The ^{1}H NMR and ^{13}C NMR spectra of **24**. 76

Supplementary Tables 77

Supplementary Table 1. The genes and primers used in this study for RT-PCR. 77

Supplementary Table 2. Primers used for constructing recombinant plasmids. 78

Supplementary Table 3. Plasmids used in this study 80

Supplementary Table 4. Strains used in the study 81

Supplementary Table 5. Differential expression analysis of cluster V 82

Supplementary Table 6. NMR data for **2** (400 MHz for ^{1}H and 100 MHz for ^{13}C, in DMSO-d_6) 83

Supplementary Table 7. NMR data for **3** (400 MHz for ^{1}H and 100 MHz for ^{13}C, in DMSO-d_6) 84

Supplementary Table 8. NMR data for **4** (400 MHz for ^{1}H and 100 MHz for ^{13}C, in DMSO-d_6) 85

Supplementary Table 9. NMR data for **5** (400 MHz for ^{1}H and 100 MHz for ^{13}C, in CD_3OD) 86

Supplementary Table 10. NMR data for **6** (300 MHz for ^{1}H and 75 MHz for ^{13}C, in DMSO-d_6) 87

Supplementary Table 11. NMR data for **7** (400 MHz for ^{1}H and 100 MHz for ^{13}C, in DMSO-d_6) 88

Supplementary Table 12. NMR data for **8** (400 MHz for ^{1}H and 100 MHz for ^{13}C, in DMSO-d_6) 89

Supplementary Table 13. NMR data for **9** (400 MHz for ^{1}H and 100 MHz for ^{13}C, in DMSO-d_6) 90

Supplementary Table 14. NMR data for **10** (600 MHz for ^{1}H and 150 MHz for ^{13}C, in DMSO-d_6) 91

Supplementary Table 15. NMR data for **13** (400 MHz for ^{1}H and 100 MHz for ^{13}C, in DMSO-d_6) 92

Supplementary Table 16. NMR data for **14** (400 MHz for ^{1}H and 100 MHz for ^{13}C, in DMSO-d_6) 93

Supplementary Table 17. NMR data for **15** (400 MHz for ^{1}H and 100 MHz for ^{13}C, in DMSO-d_6) 94

Supplementary Notes.... 95

Supplementary Note 1. Structural characterization of **1** 95

Supplementary Note 2. Structural characterization of **2** 96

Supplementary Note 3. Structural characterization of **3** 97

Supplementary Note 4. Structural characterization of **4** 98

Supplementary Note 5. Structural characterization of **5** 99

Supplementary Note 6. Structural characterization of **8** 100

Supplementary Note 7. Structural characterization of **7** 101

Supplementary Note 8. Structural characterization of **6** 102

Supplementary Note 9. Structural characterization of **9** 103

Supplementary Note 10. Structural characterization of **11**.... 104

Supplementary Note 11. Structural characterization of **10**.... 105

Supplementary Note 12. Structural characterization of **12** 106

Supplementary Note 13. Structural characterization of **13** 107

Supplementary Note 14. Structural characterization of **14** 108

Supplementary Note 15. Structural characterization of **15** 109

Supplementary Note 16. Structural characterization of **21** 110

Supplementary Note 17. Structural characterization of **24** 111

Supplementary Note 18. X-ray crystallographic analysis of **5**. 112

Supplementary Note 19. X-ray crystallographic analysis of **8**. 113

Supplementary Note 20. X-ray crystallographic analysis of **13**. 114

Supplementary Methods 115

Supplementary References.... 117

Supplementary Figures

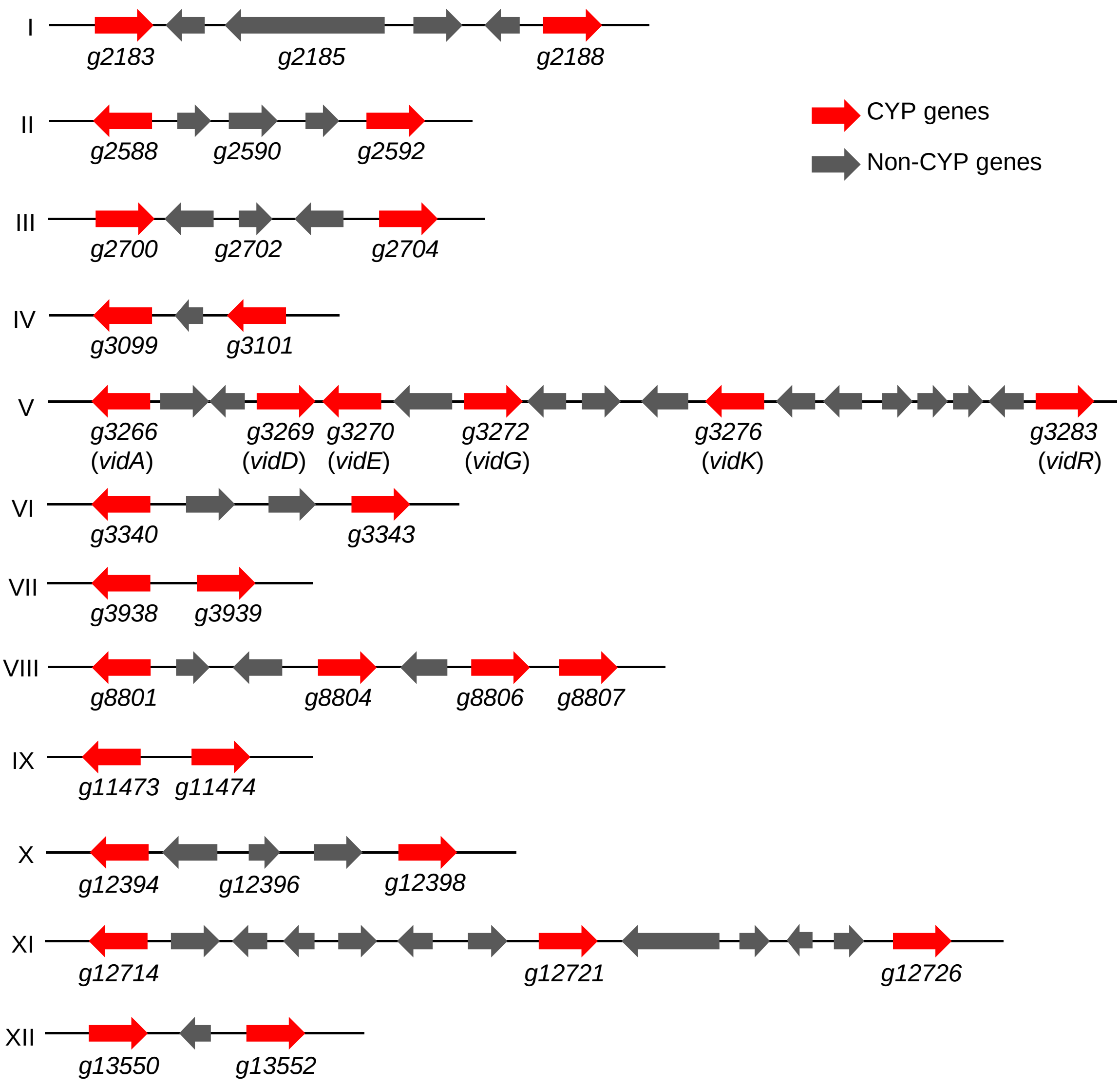

Supplementary Figure 1. Twelve CYP clusters in the genome of *Nodulisporium* sp. (No. 65-12-7-1).

CYP genes are indicated by red arrows, and the non-CYP genes are indicated by the gray arrows.

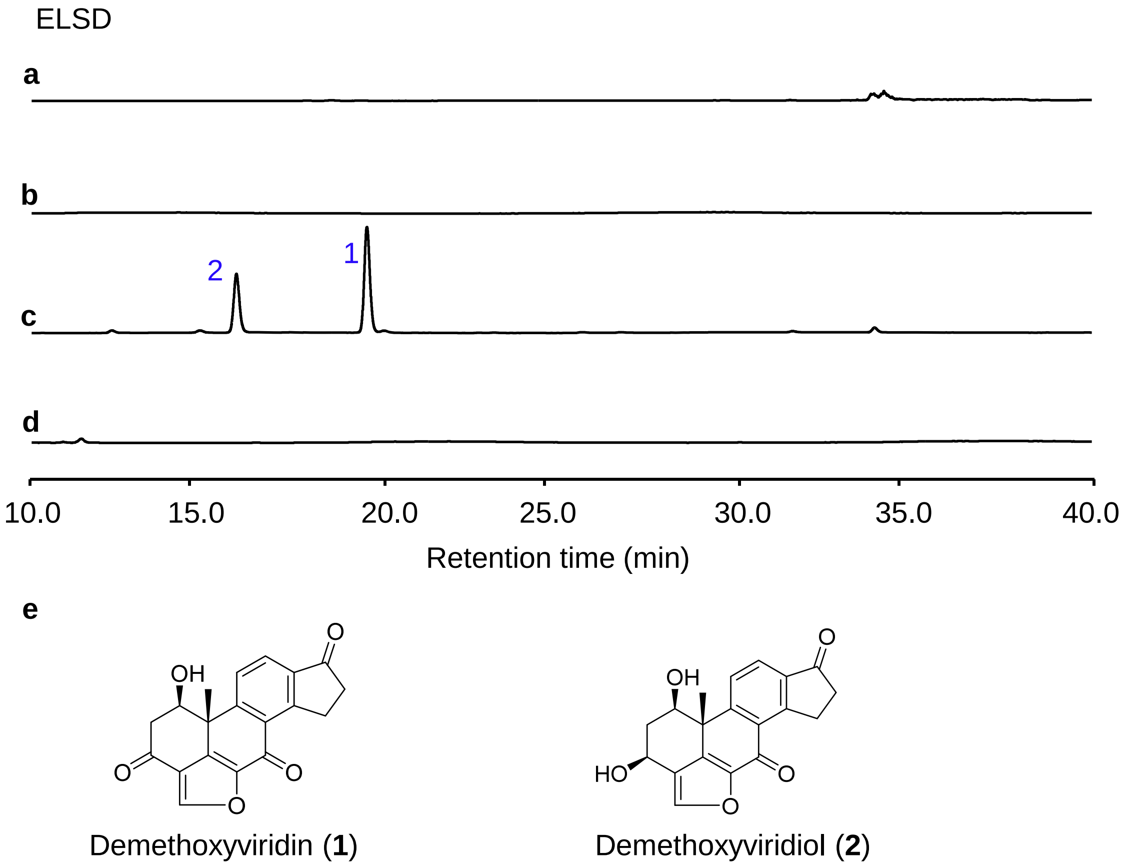

Supplementary Figure 2. **Production of demethoxyviridin and its derivative by *Nodulisporium* sp. (No. 65-12-7-1) under different culture conditions.**

(**a**) Extract of mycelia cultured in Czapek; (**b**) Extract of Czapek medium; (**c**) Extract of mycelia cultured in Maltose; (**d**) Extract of Maltose medium; (**e**) Structures of compounds **1** and **2**. Fermentation was performed with shaking at 180 rpm at 28 °C for 2 days. Maltose: demethoxyviridin productive medium; Czapek: demethoxyviridin non-productive medium.

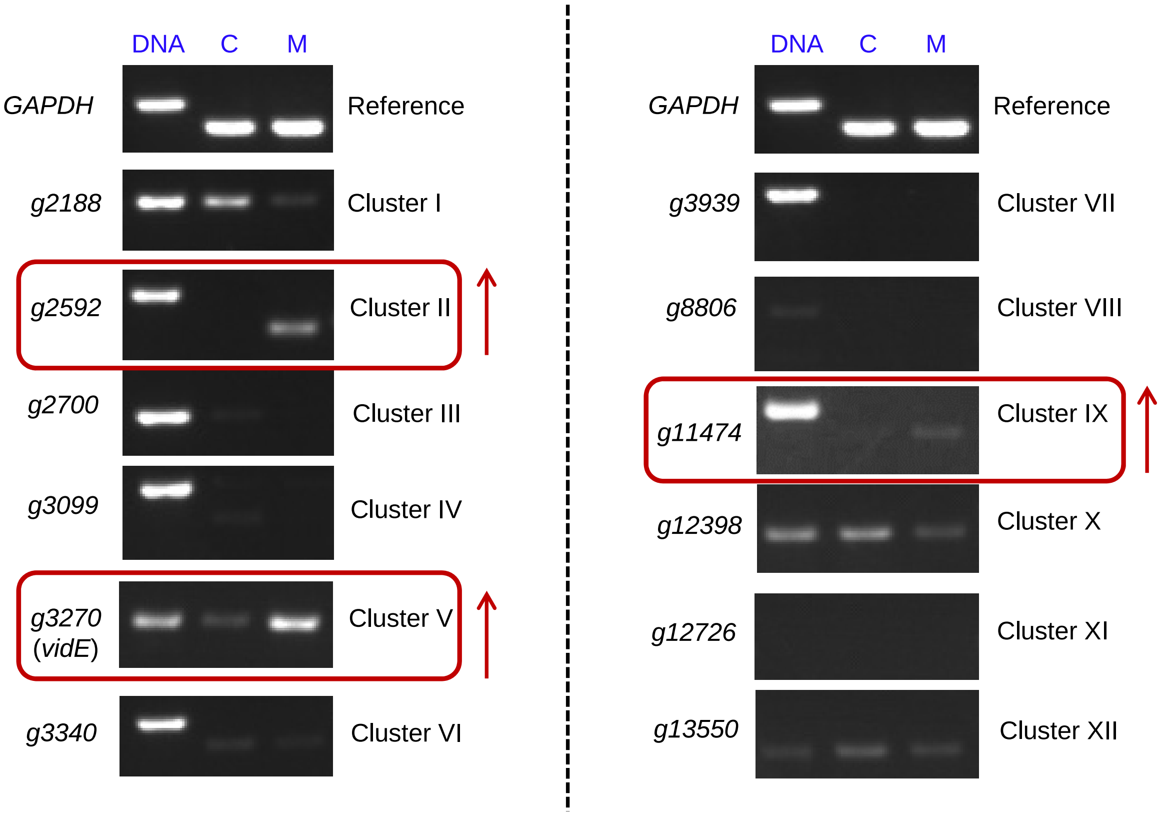

Supplementary Figure 3.Expression analysis of a randomly selected CYP gene from each of the twelve CYP clusters.

One CYP gene from each of the twelve CYP clusters was randomly selected and its expression was analyzed by RT-PCR with GAPDH as the reference gene. **DNA**: Genomic DNA of *Nodulisporium* sp. (No. 65-12-7-1); **C**: Gene expression when cultured in Czapek medium; **M**: Gene expression when cultured in Maltose medium. The genes which were up-regulated in maltose medium were boxed.

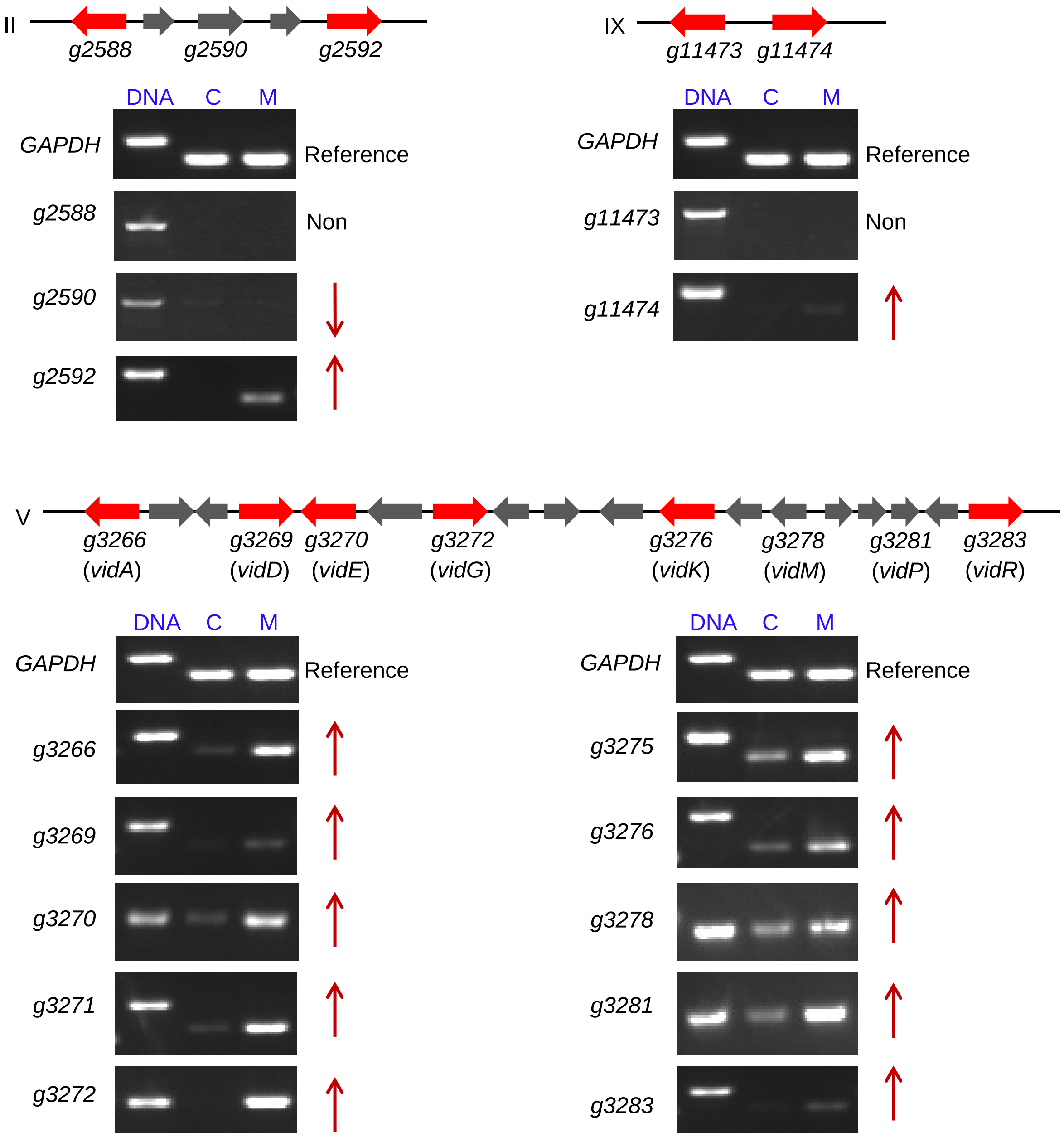

Supplementary Figure 4. Expression analysis of the three candidate CYP clusters.

The expression of all the genes in the three canidate CYP clusters was analyzed by RT-PCR with GAPDH as the reference gene. **DNA**: Genomic DNA of *Nodulisporium* sp. (No. 65-12-7-1); **C**: Gene expression when cultured in Czapek medium; **M**: Gene expression when cultured in Maltose medium. The up and down arrows indicate the up-regulated and down-regulated genes in maltose medium, respectively. Non means that the genes could not be amplified.

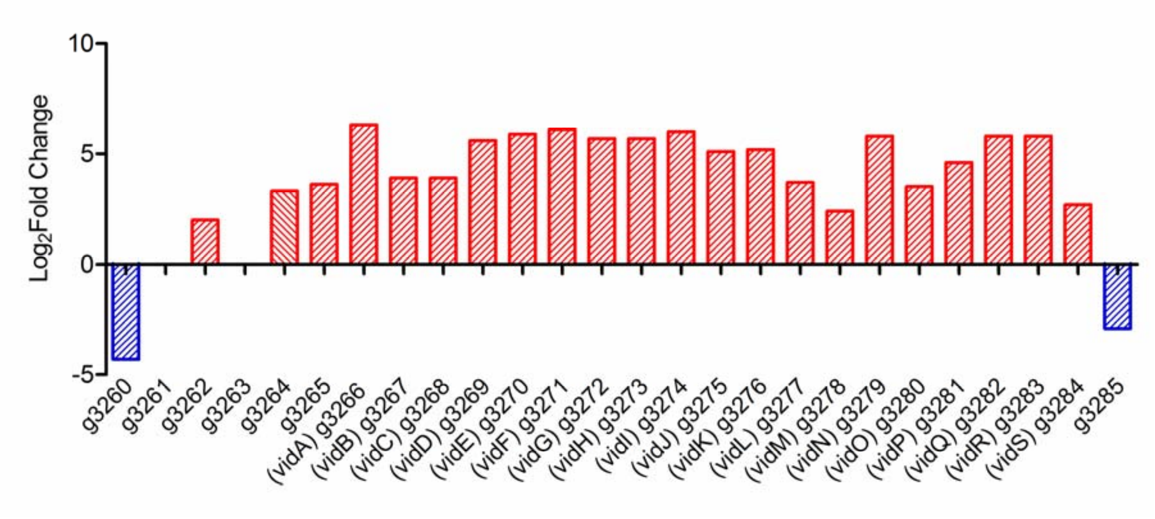

Supplementary Figure 5. Expression analysis of the genes in cluster V and its adjacent genes by RNA-Seq.

The expression level of the genes in cluster V and its adjacent genes in Maltose medium (M) relative to Czapek medium (C) was quantified using the *DESeq* with a FDR < 0.05 and an absolute value of fold-change $\geqslant$ 2. The Log_2Fold Change of each gene in cluster V is shown in **Supplementary Table 5**.

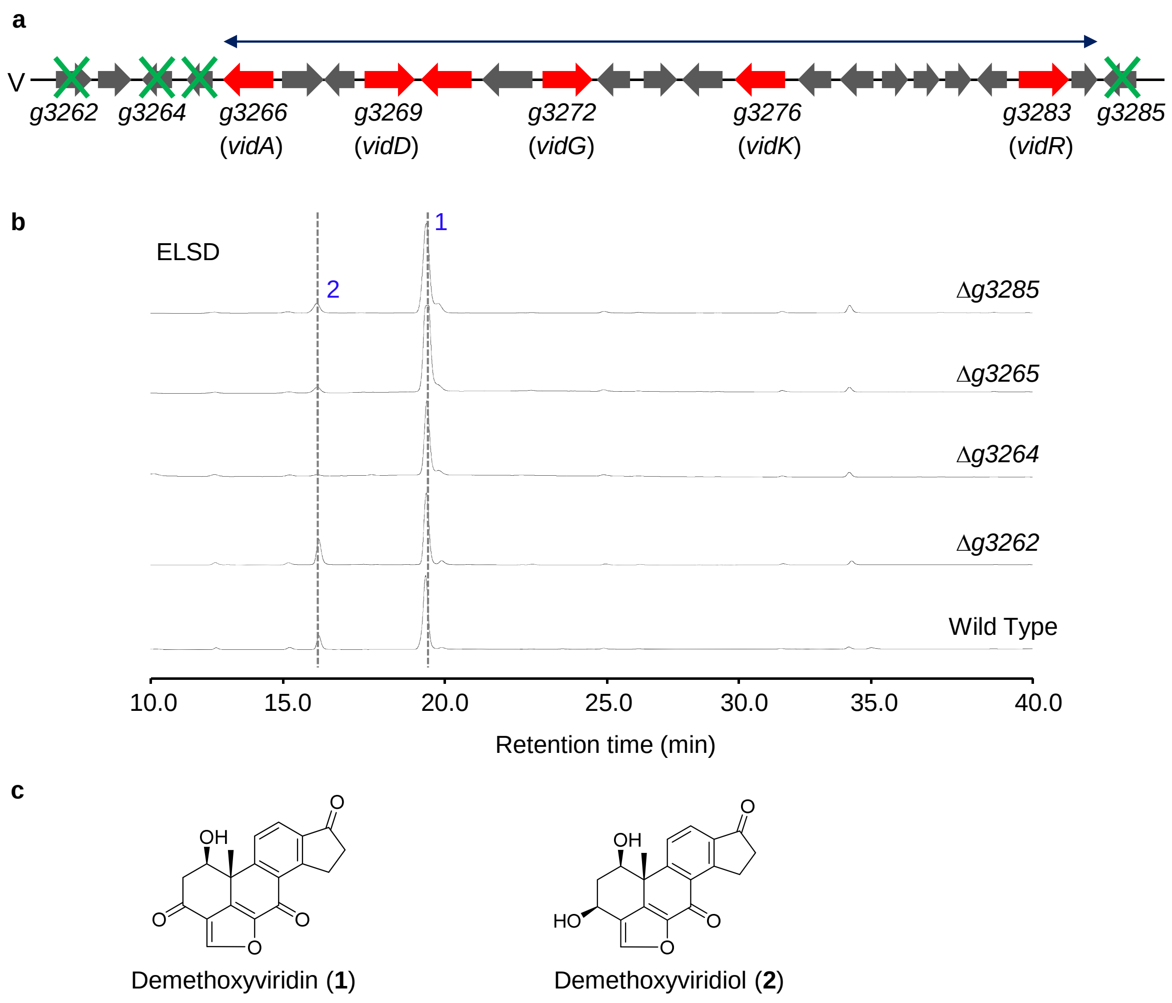

Supplementary Figure 6. Determination of the boundary of the biosynthetic gene cluster for demethoxyviridin.

(**a**) Schematic representation of the boundary of the biosynthetic gene cluster for demethoxyvirdin; (**b**) HPLC profiles of culture extracts from the gene deletion mutants; (**c**) Structures of compounds **1**−**2**.

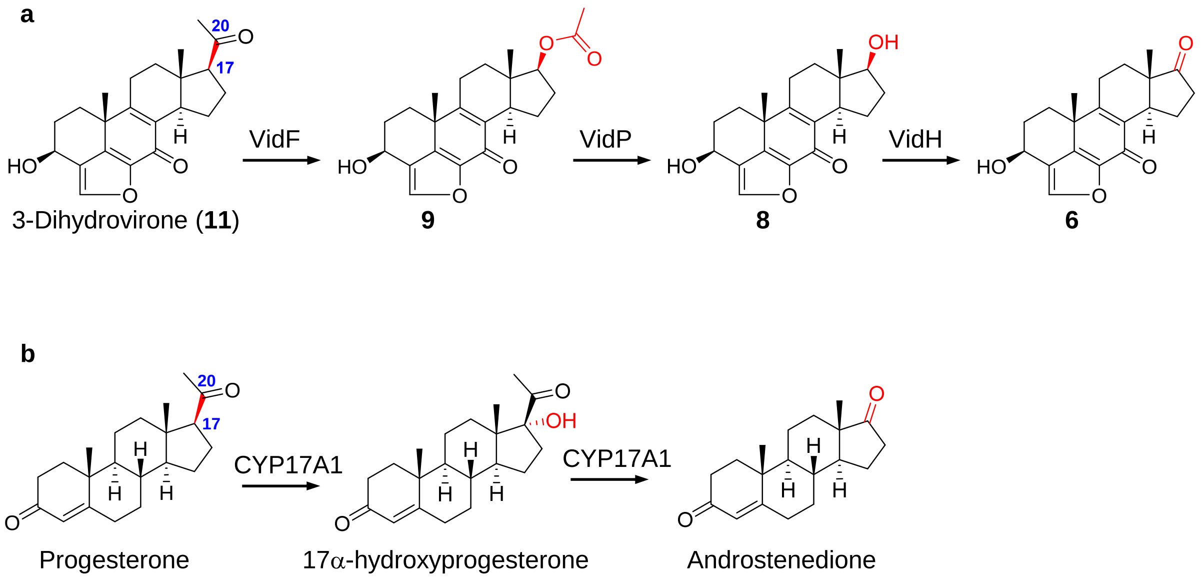

Supplementary Figure 7. **Two different pregnane side-chain cleavage processes.**

(**a**) The pregnane side-chain cleavage mediated by three enzymes VidF, VidP, and VidH during biosynthesis of demethoxyviridin in fungi; (**b**) The pregnane side-chain cleavage mediated by a single CYP enzyme CYP17A1 during biosynthesis of steroid hormones in mammalian cells.

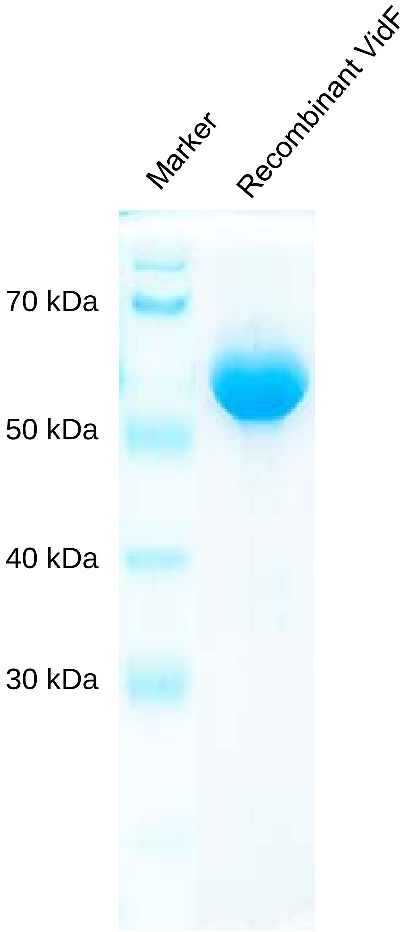

Supplementary Figure 8. SDS-PAGE analysis for purified recombinant VidF.

VidF tagged with *N*-terminal 6 × His was purified using nickel affinity chromatography. The molecular weight of recombinant VidF was about 61 kDa.

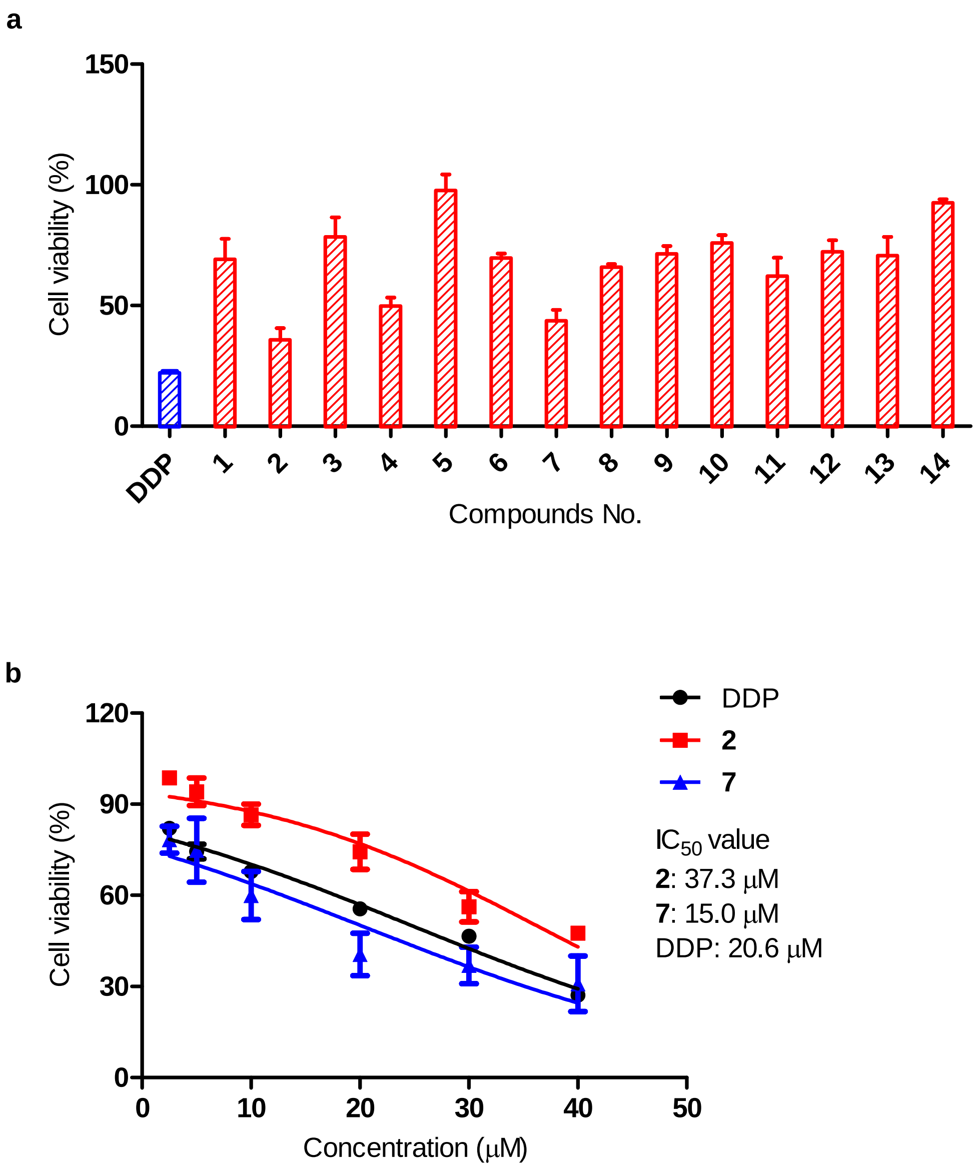

Supplementary Figure 9. The cytotoxicity of compounds 1−14 against breast cancer cell line MCF-7.

(**a**) The cytotoxicity of compounds **1**–**14** against MCF-7 at 40 μM with cisplatin (DDP) as the positive control. The absorbance (Ab) of samples was detected at 450 nm using a microtiter plate reader. The cell viability of each sample was calculated by the following formula: $[(Ab_{sample}–Ab_{blank})/(Ab_{DMSO}–Ab_{blank})] \times 100\%$. Data represent mean ± SD of three replicates; (**b**) The IC_{50} values of compounds **2**, **7**, and DDP. The IC_{50} values were calculated using the "Sigmoidal dose-response (variable slope)" equation in the GraphPad Prism® Version 5.0 software. Data represent mean ± SD of three replicates.

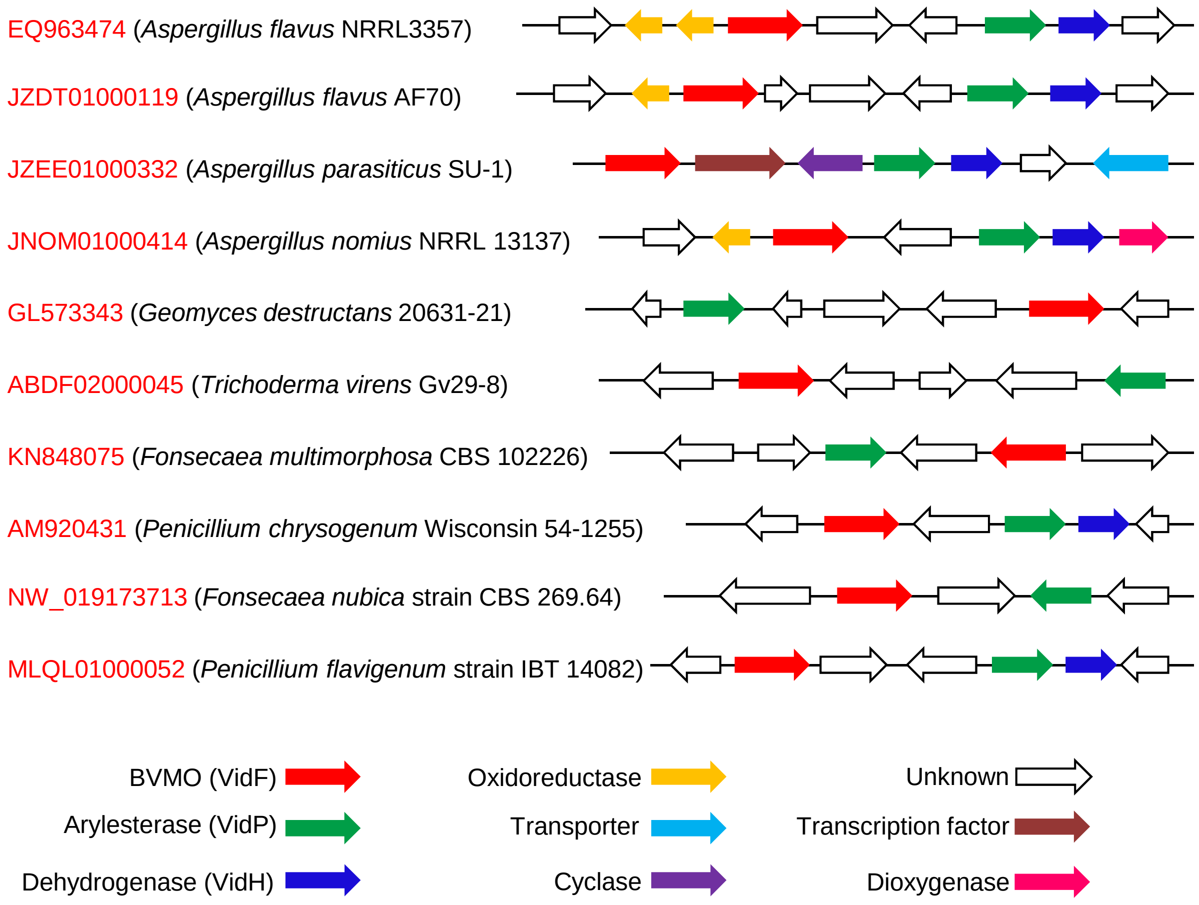

Supplementary Figure 10. Representative gene clusters containing enzymes for pregnane side-chain cleavage from fungi.

MultiGeneBlast, a program that uses a combination of BLAST and synteny, was used to identify the gene clusters for pregenane side-chain cleavage, which are classified by at least containing the homologues of VidF (red color) and VidP (green color). MultiGeneBlast was run with the 0.5 weight of synteny conservation in hit sorting, the minimal 25% sequence coverage of BLAST hits, the minimal 30% identity of BLAST hits, and the 20 kb maximum distance between genes in locus.

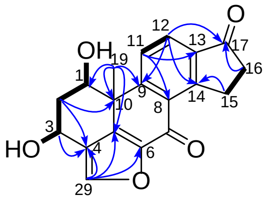

^{1}H-^{1}H COSY

HMBC

2

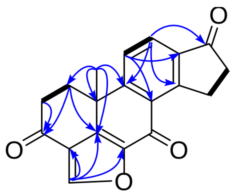

^{1}H-^{1}H COSY

HMBC

3

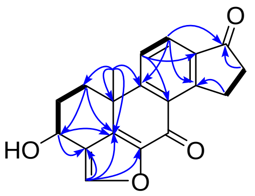

^{1}H-^{1}H COSY

HMBC

4

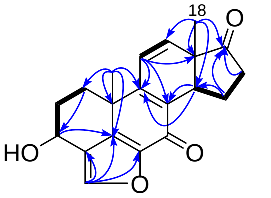

^{1}H-^{1}H COSY

HMBC

5

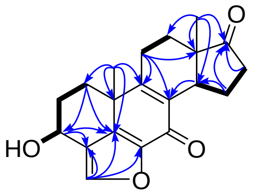

^{1}H-^{1}H COSY

HMBC

6

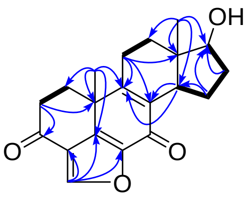

^{1}H-^{1}H COSY

HMBC

7

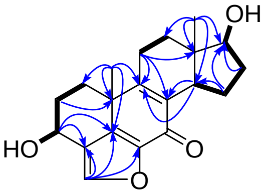

^{1}H-^{1}H COSY

HMBC

8

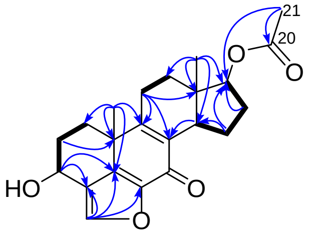

^{1}H-^{1}H COSY

HMBC

9

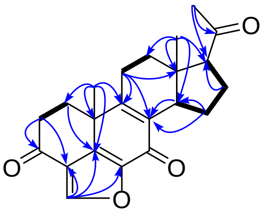

^{1}H-^{1}H COSY

HMBC

10

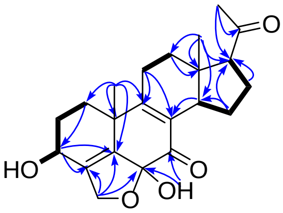

^{1}H-^{1}H COSY

HMBC

13

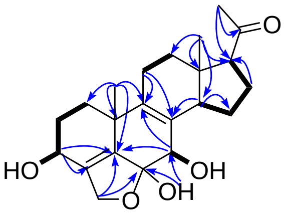

^{1}H-^{1}H COSY

HMBC

14

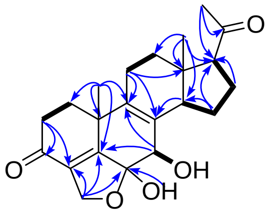

^{1}H-^{1}H COSY

HMBC

15

Supplementary Figure 11. Key ^{1}H–^{1}H COSY and HMBC correlations.

The correlations shown here are key ^{1}H–^{1}H COSY and HMBC for structural determinations of **2–10**, and **13–15**.

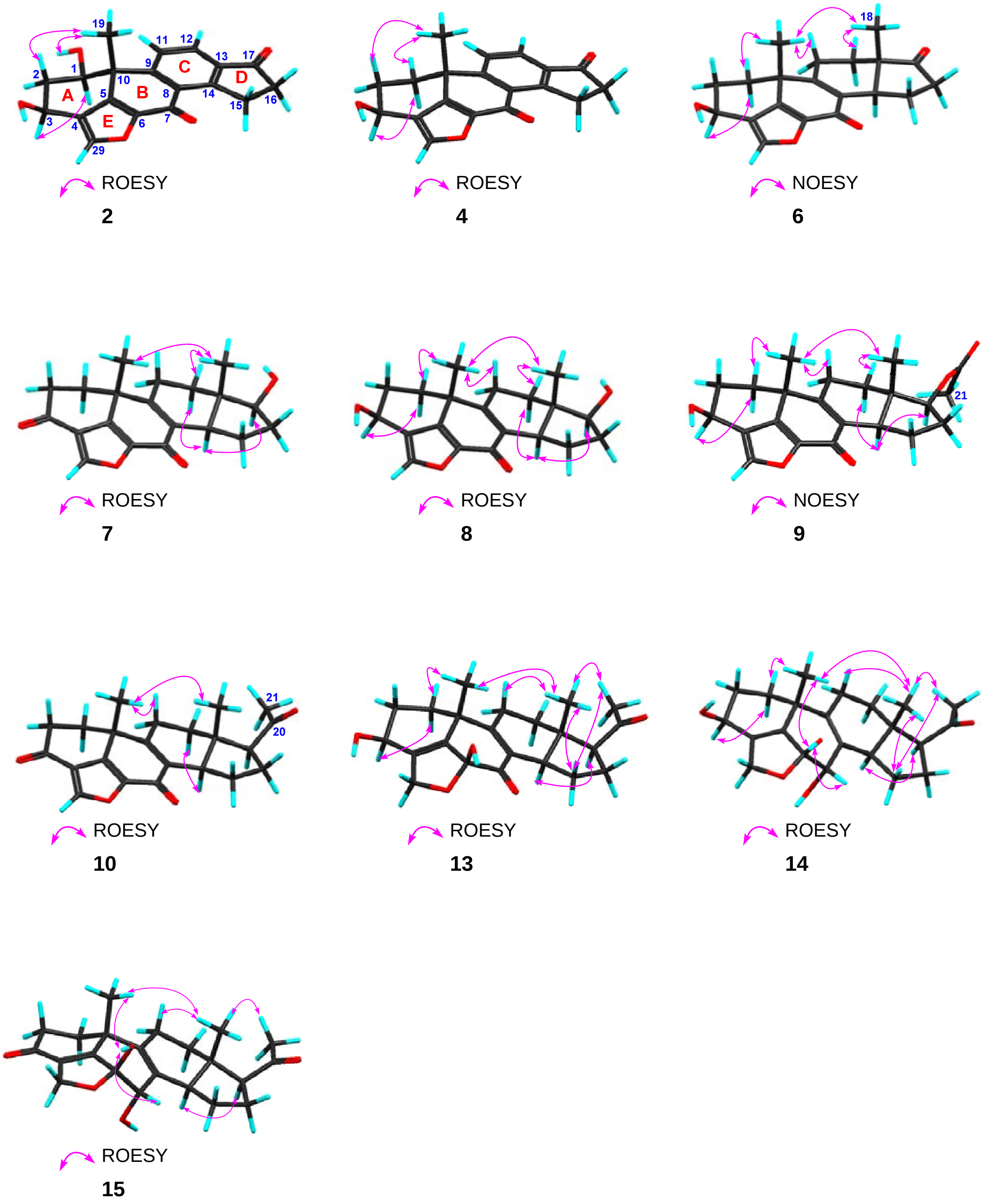

Supplementary Figure 12. Key ROESY or NOESY correlations.

The correlations shown here are key ROESY or NOESY for structural determinations of **2**, **4**, **6**–**10**, and **13**–**15**.

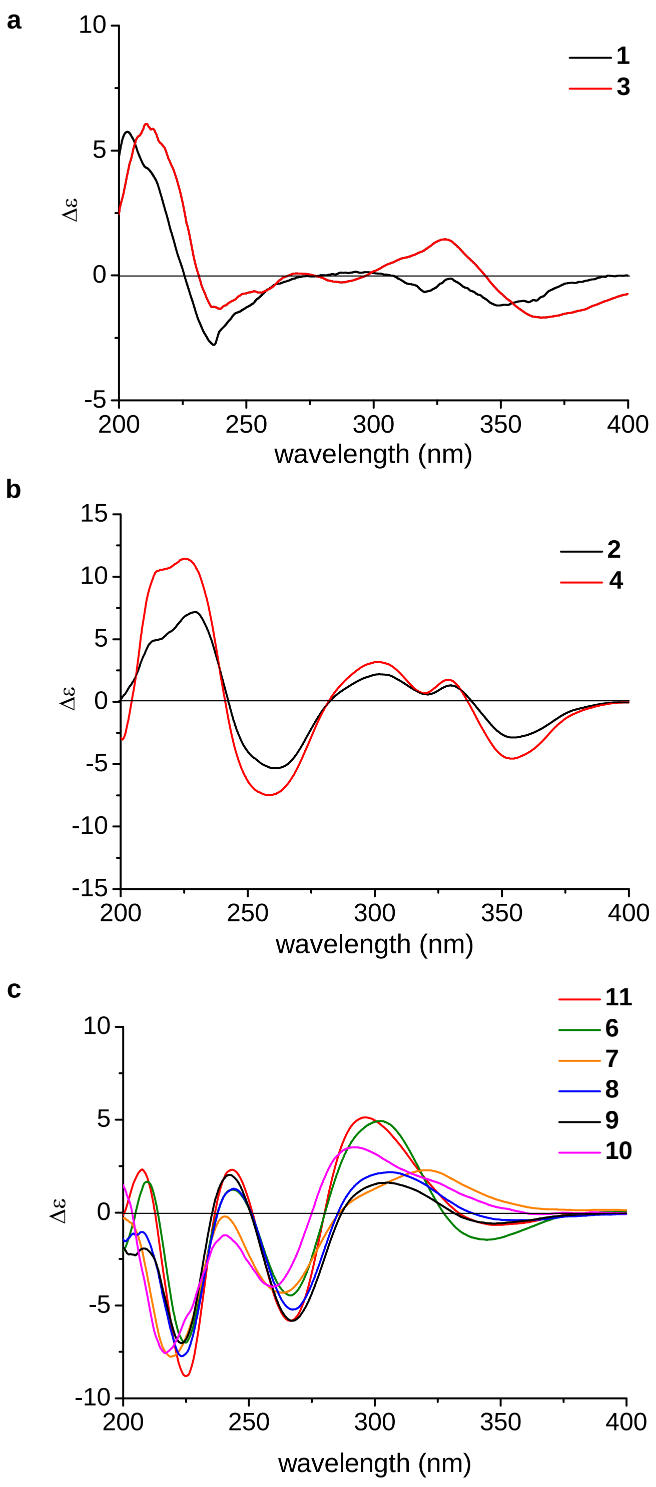

Supplementary Figure 13. Experimental ECD spectra of 1–4 and 6–11.

(**a**) The ECD spectra of **1** and **3**; (**b**) The ECD spectra of **2** and **4**; (**c**) The ECD spectra of **6–11**.

Single Mass Analysis
Tolerance = 10.0 PPM / DBE: min = -1.5, max = 50.0
Element prediction: Off
Number of isotope peaks used for i-FIT = 3

Monoisotopic Mass, Even Electron Ions
64 formula(e) evaluated with 1 results within limits (up to 50 closest results for each mass)
Elements Used:
C: 0-100 H: 0-100 O: 0-50
DMV-1
2017110603 106 (0.860)

1: TOF MS ES+
2.14e+005

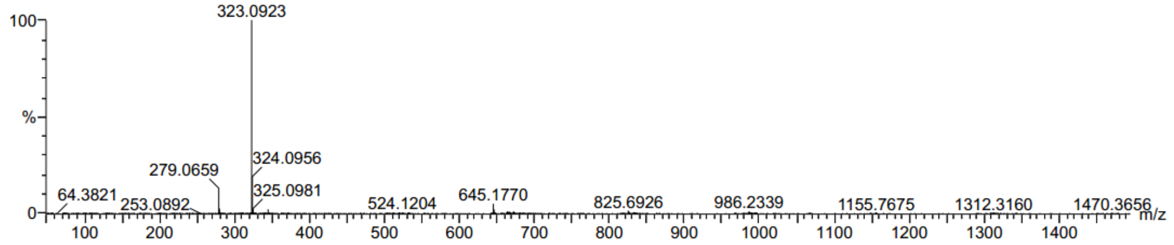

Minimum:				-1.5				
Maximum:		5.0	10.0	50.0				
Mass	Calc. Mass	mDa	PPM	DBE	i-FIT	Norm	Conf(%)	Formula
323.0923	323.0919	0.4	1.2	12.5	269.7	n/a	n/a	C19 H15 O5

Supplementary Figure 14. The HRESIMS spectrum of 1.

The HRESIMS spectrum (positive) showed *m/z* 323.0923 $[M + H]^+$ (calcd. for $C_{19}H_{15}O_5$, 323.0919

a

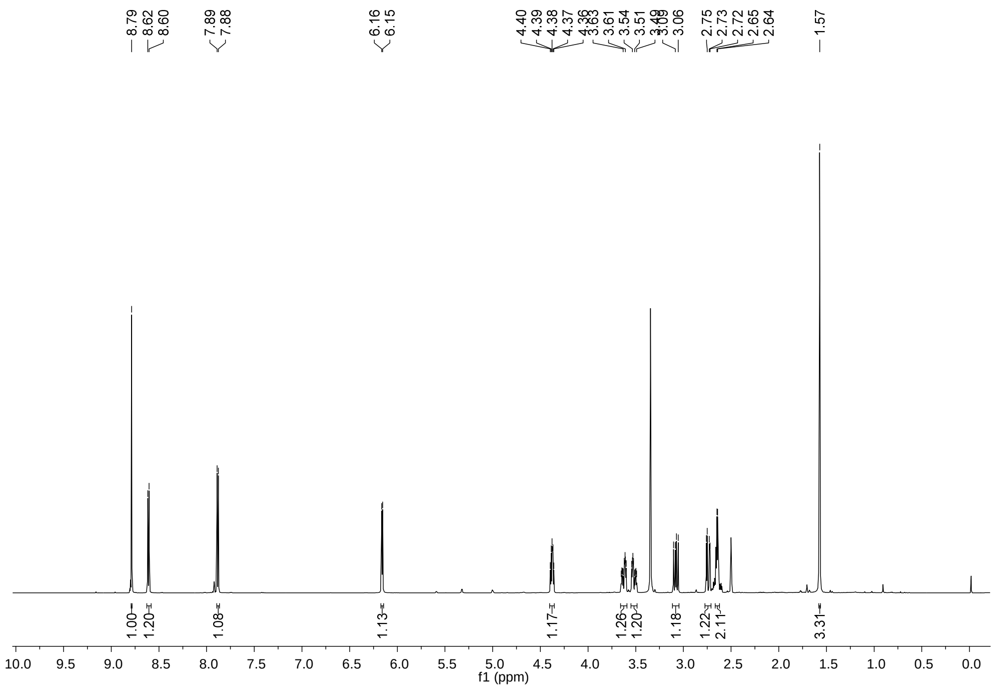

b

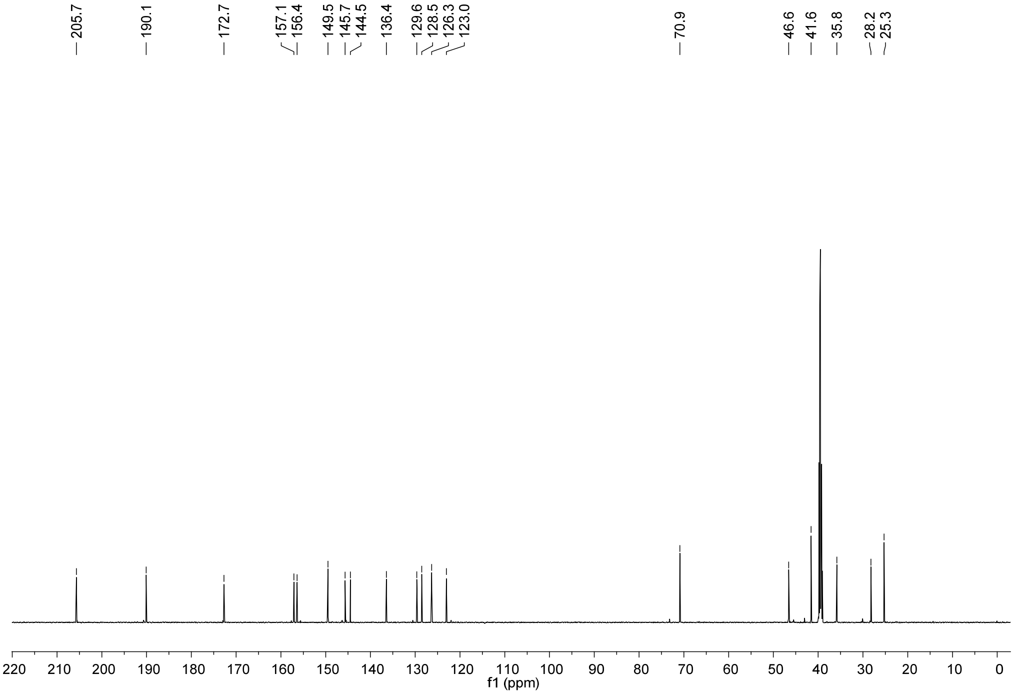

Supplementary Figure 15. The ^{1}H NMR and ^{13}C NMR spectra of 1.

(**a**) ^{1}H NMR spectrum in DMSO-d_6 at 600 MHz; (**b**) ^{13}C NMR spectrum in DMSO-d_6 at 150 MHz.

Single Mass Analysis
Tolerance = 30.0 mDa / DBE: min = -1.5, max = 50.0
Element prediction: Off
Number of isotope peaks used for i-FIT = 3

Monoisotopic Mass, Even Electron Ions
112 formula(e) evaluated with 15 results within limits (up to 50 closest results for each mass)
Elements Used:
C: 0-35 H: 0-110 O: 0-50 Na: 0-1
76-2
20170423-20 67 (0.558)

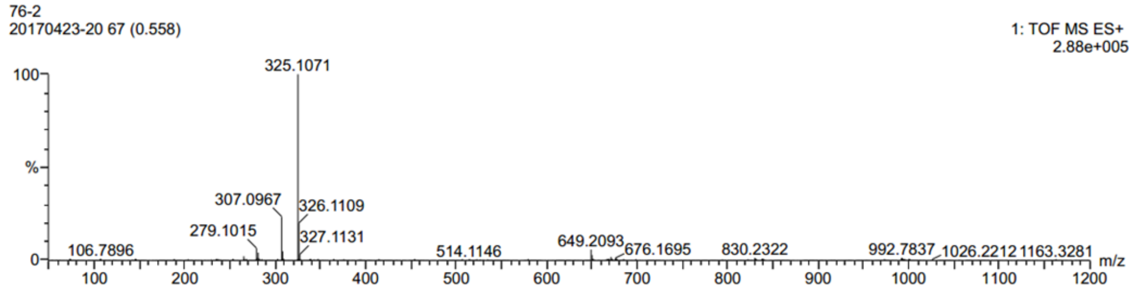

Minimum:				-1.5
Maximum:		30.0	10.0	50.0

Mass	Calc. Mass	mDa	PPM	DBE	i-FIT	Norm	Conf(%)	Formula
325.1071	325.1076	-0.5	-1.5	11.5	342.3	1.228	29.28	C19 H17 O5
	325.1052	1.9	5.8	8.5	341.5	0.366	69.33	C17 H18 O5 Na

Supplementary Figure 16. The HRESIMS spectrum of 2.

The HRESIMS spectrum (positive) showed *m/z* 325.1071 $[M + H]^+$ (calcd. for $C_{19}H_{17}O_5$, 325.1076).

a

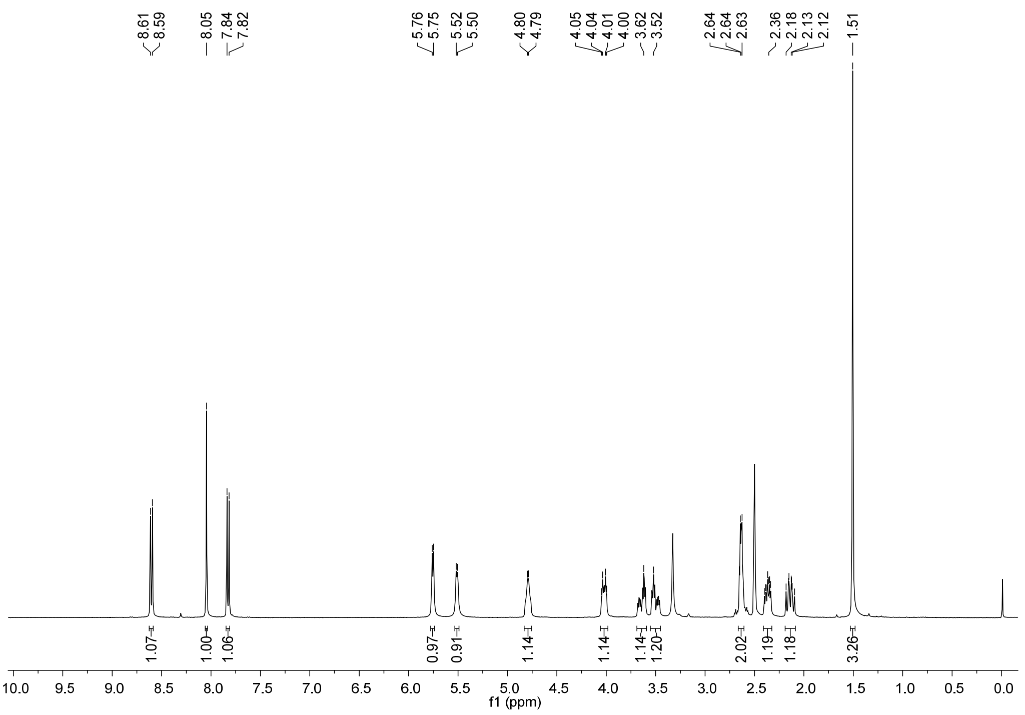

b

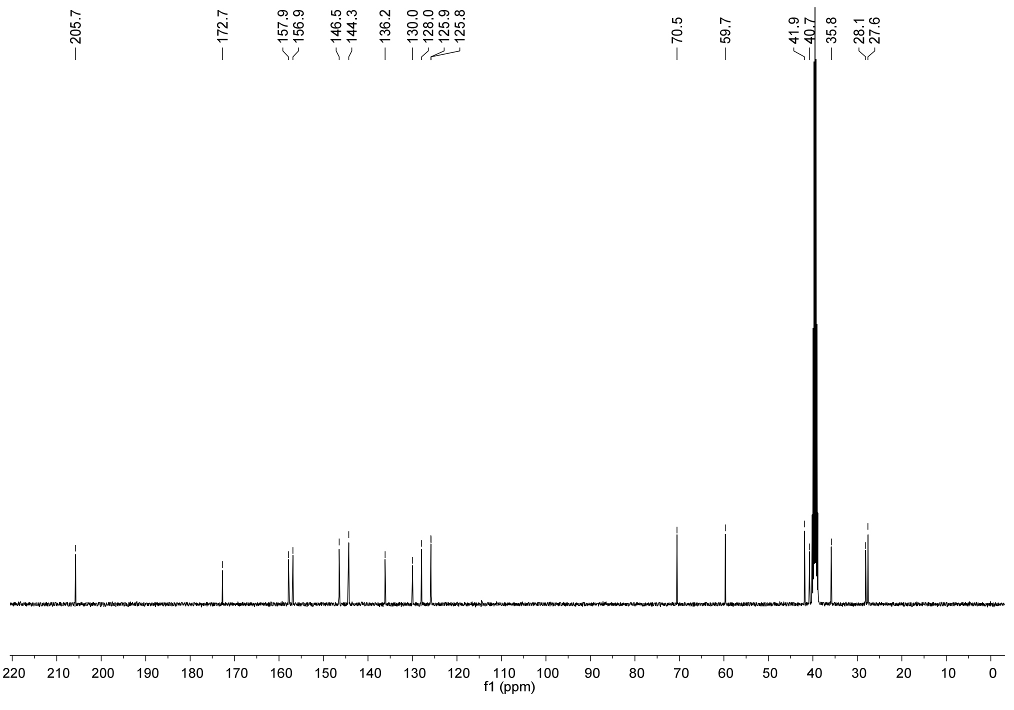

Supplementary Figure 17. The ^{1}H NMR and ^{13}C NMR spectra of 2.

(**a**) ^{1}H NMR spectrum in DMSO-d_6 at 400 MHz; (**b**) ^{13}C NMR spectrum in DMSO-d_6 at 100 MHz.

a

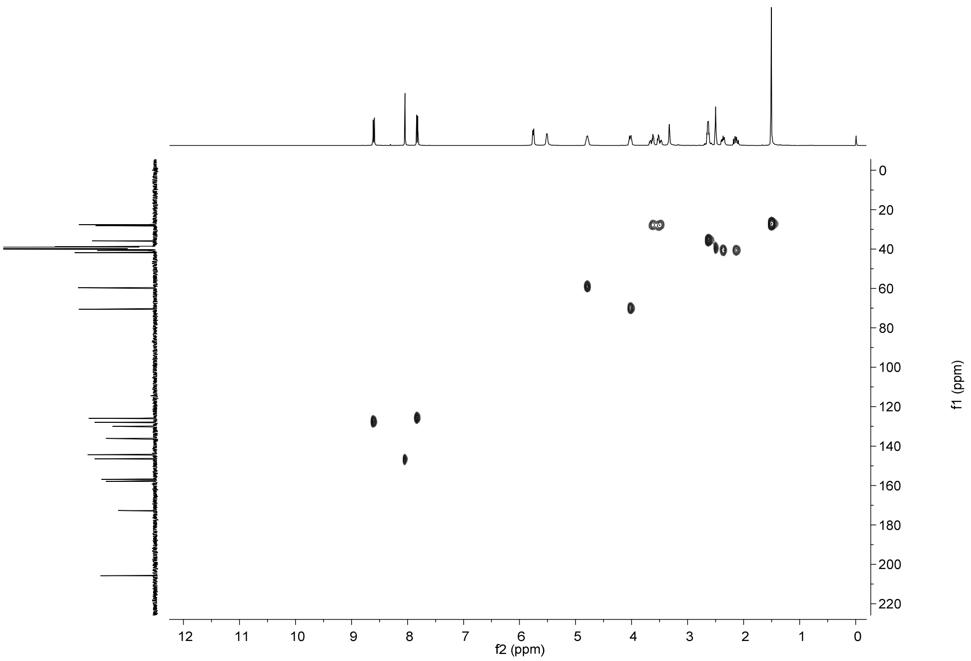

b

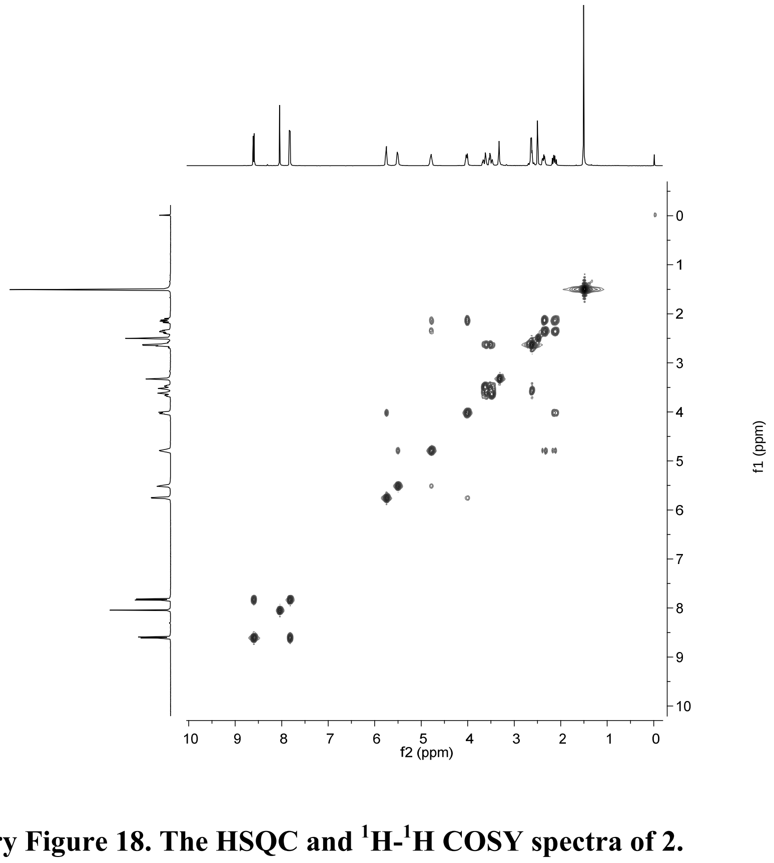

Supplementary Figure 18. The HSQC and ^{1}H-^{1}H COSY spectra of 2.

(**a**) HSQC spectrum in DMSO-d_6 at 400 MHz; (**b**) ^{1}H-^{1}H COSY spectrum in DMSO-d_6 at 400 MHz.

a

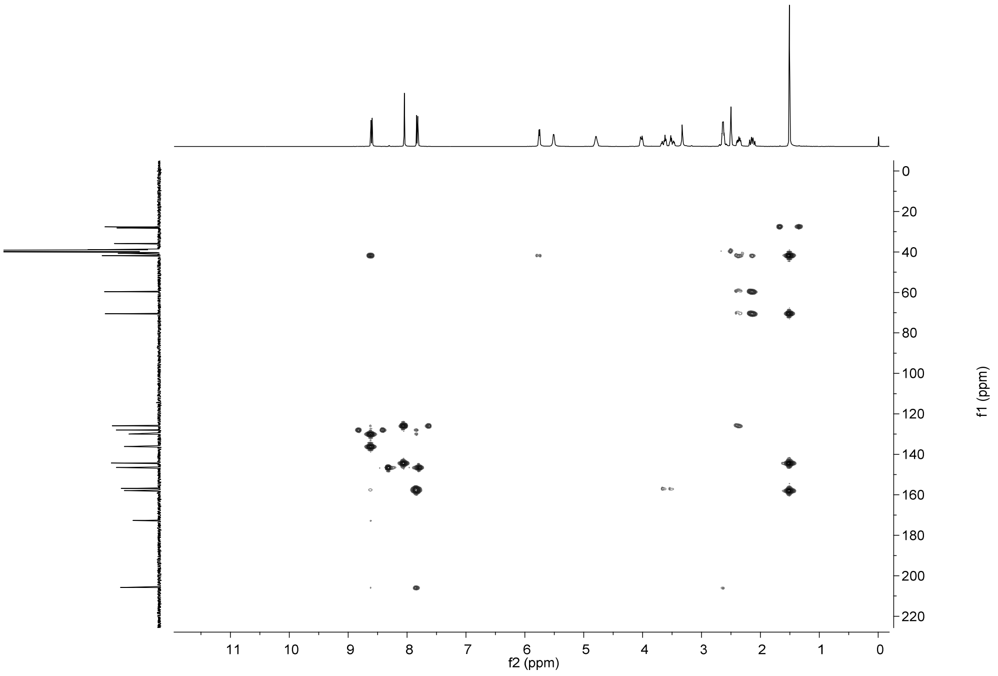

b

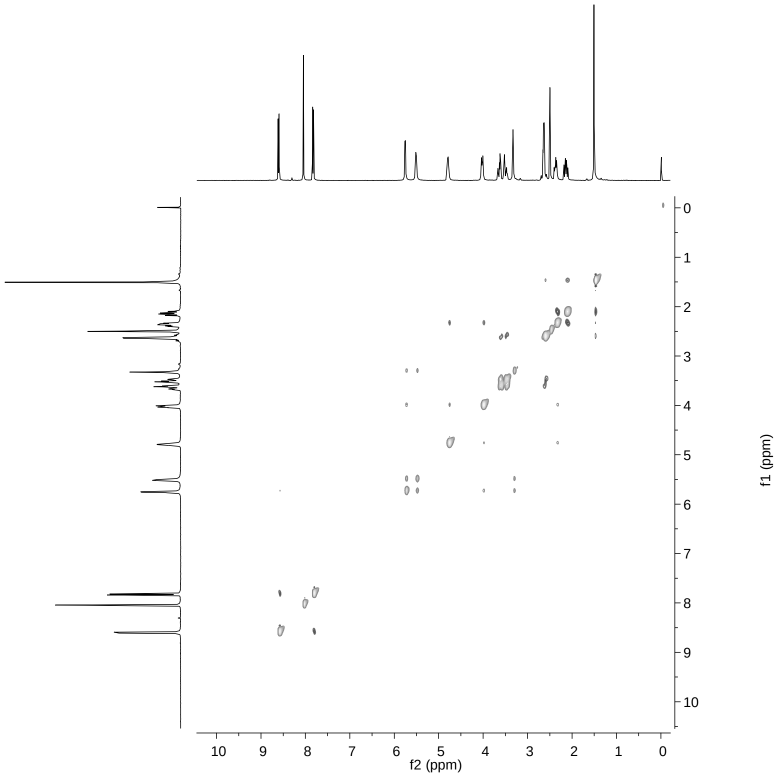

Supplementary Figure 19. The HMBC and ROESY spectra of 2.

(**a**) HMBC spectrum in DMSO-d_6 at 400 MHz; (**b**) ROESY spectrum in DMSO-d_6 at 400 MHz.

Single Mass Analysis
Tolerance = 10.0 PPM / DBE: min = -1.5, max = 50.0
Element prediction: Off
Number of isotope peaks used for i-FIT = 3

Monoisotopic Mass, Even Electron Ions
167 formula(e) evaluated with 2 results within limits (up to 20 best isotopic matches for each mass)
Elements Used:
C: 0-100 H: 0-200 N: 0-2 O: 0-100

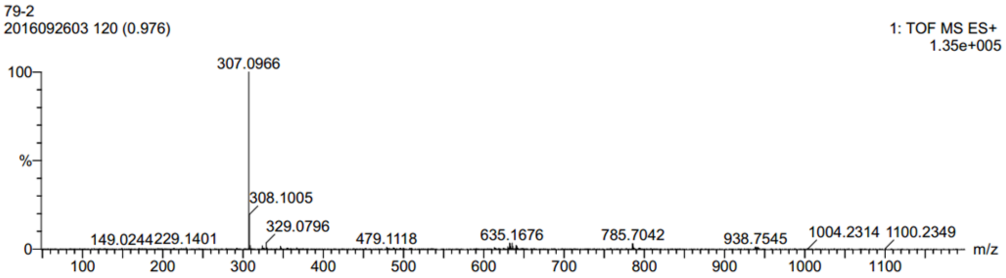

Minimum:				-1.5				
Maximum:		5.0	10.0	50.0				

Mass	Calc. Mass	mDa	PPM	DBE	i-FIT	Norm	Conf(%)	Formula
307.0966	307.0970	-0.4	-1.3	12.5	173.7	0.000	99.98	C19 H15 O4

Supplementary Figure 20. The HRESIMS spectrum of 3.

The HRESIMS spectrum (positive) showed *m/z* 307.0966 $[M + H]^+$ (calcd. for $C_{19}H_{15}O_4$, 307.0970

a

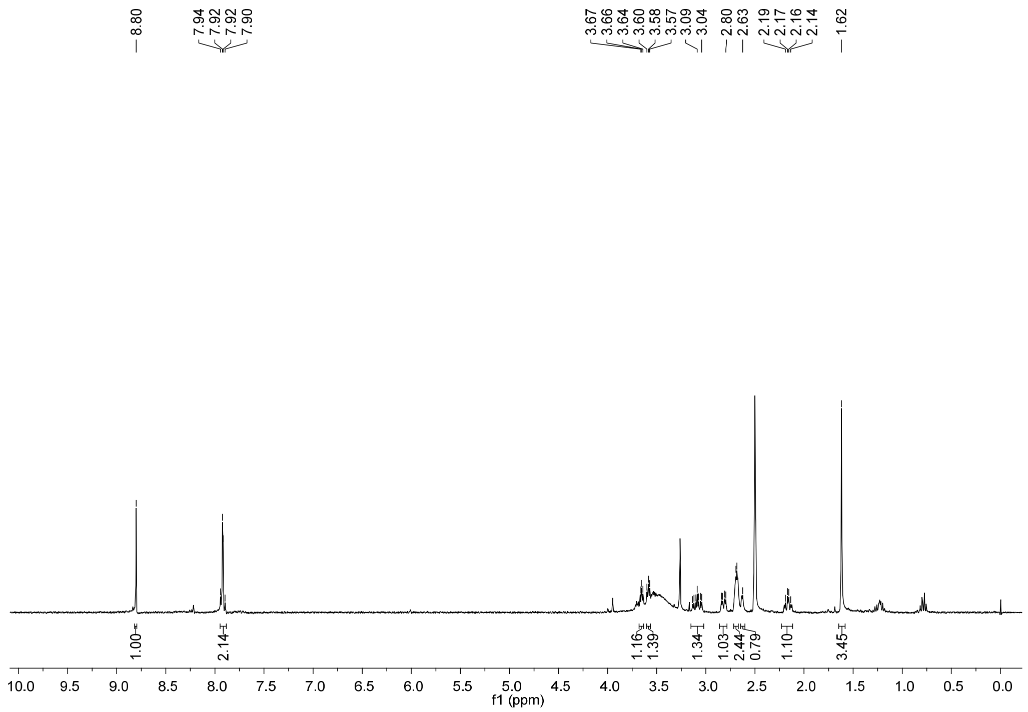

b

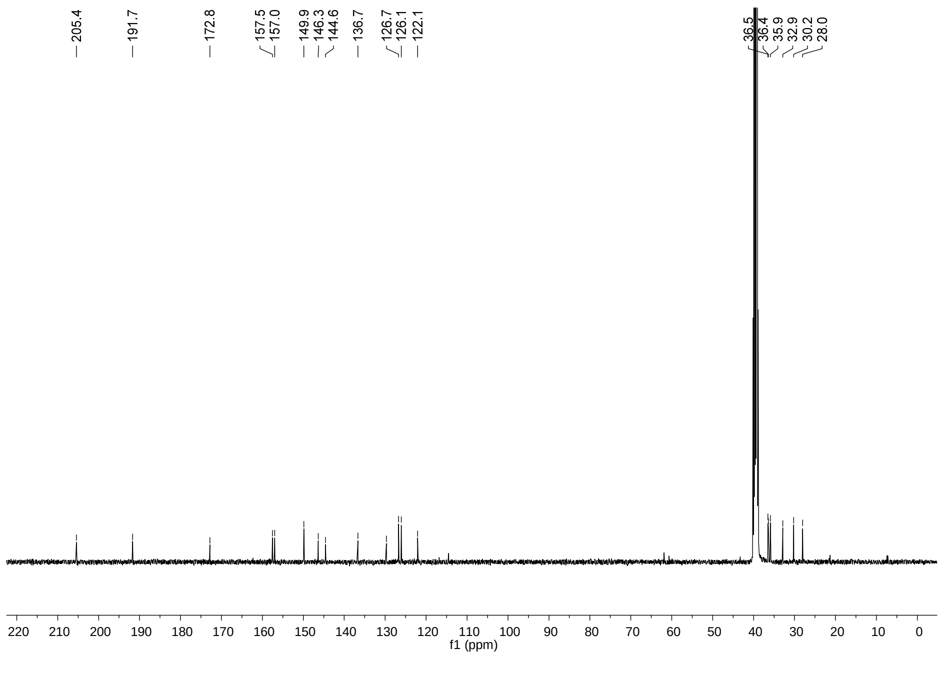

Supplementary Figure 21. The ^{1}H NMR and ^{13}C NMR spectra of 3.

(**a**) ^{1}H NMR spectrum in DMSO-d_6 at 400 MHz; (**b**) ^{13}C NMR spectrum in DMSO-d_6 at 100 MHz.

a

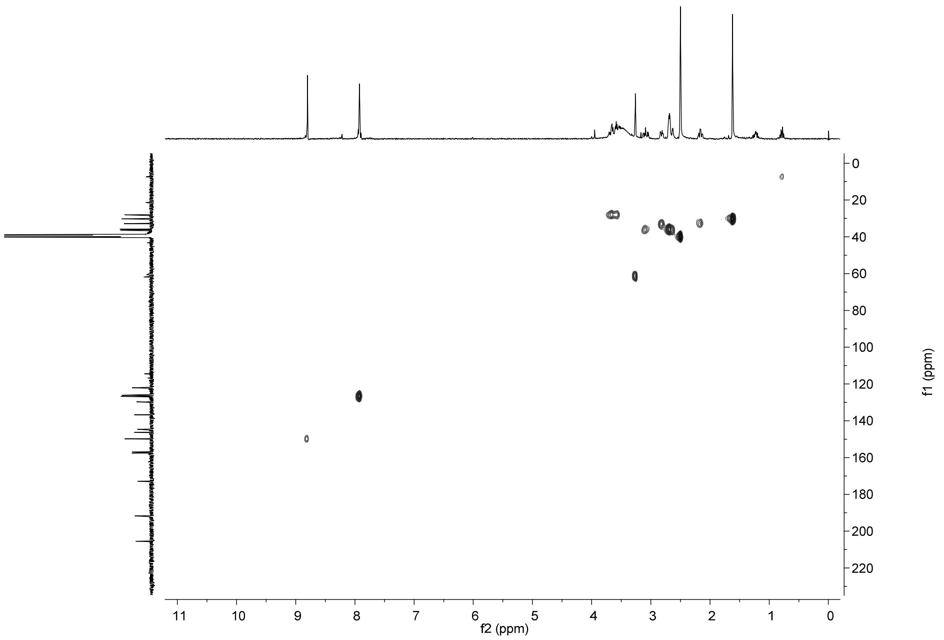

b

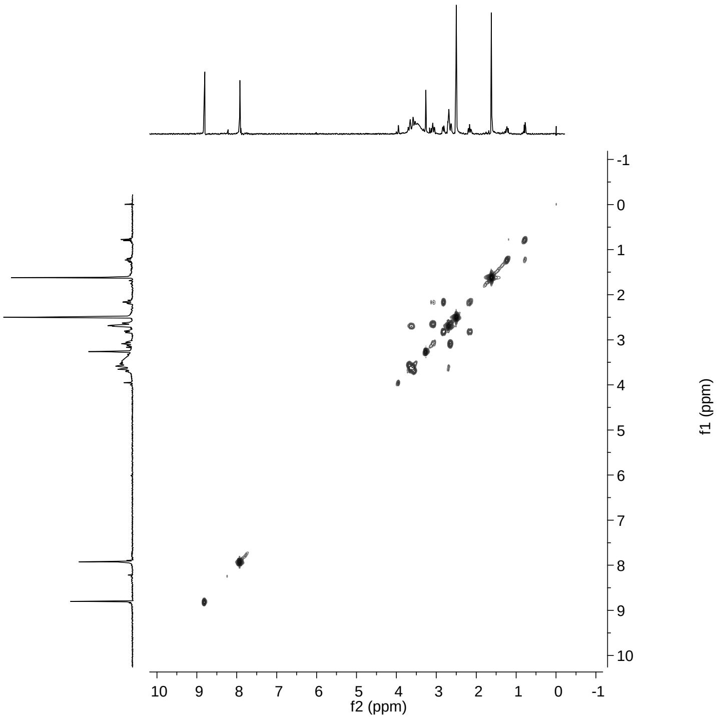

Supplementary Figure 22. The HSQC and ^{1}H-^{1}H COSY spectra of 3.

(**a**) HSQC spectrum in DMSO-d_6 at 400 MHz; (**b**) ^{1}H-^{1}H COSY spectrum in DMSO-d_6 at 400 MHz.

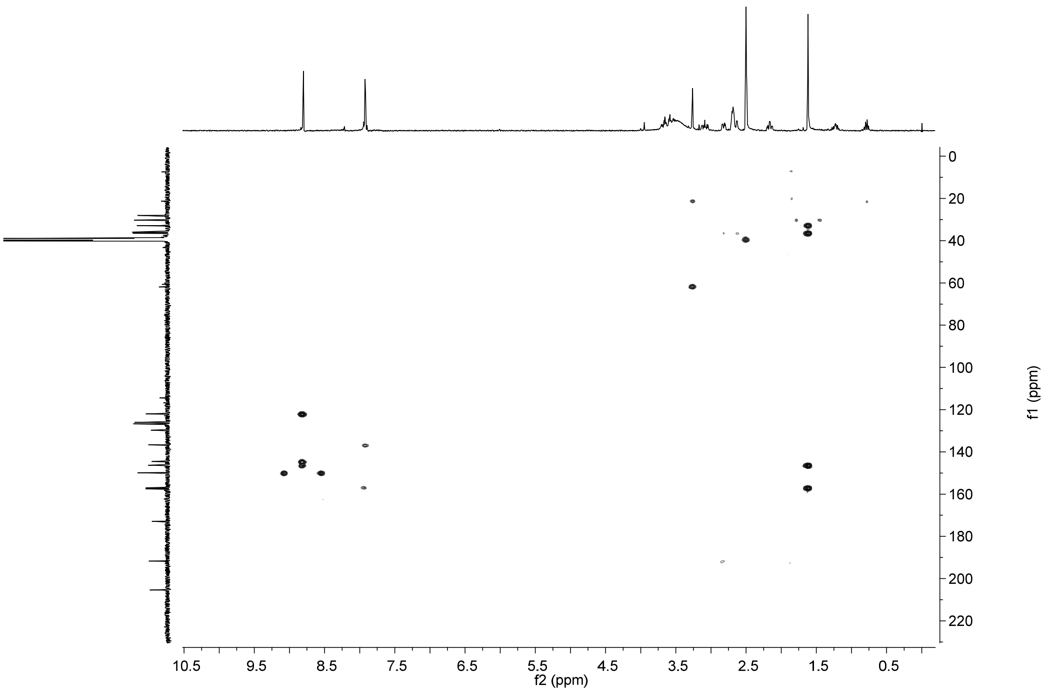

Supplementary Figure 23. The HMBC spectrum of 3.

The HMBC spectrum in DMSO-d_6 at 400 MHz.

Single Mass Analysis
Tolerance = 10.0 PPM / DBE: min = -1.5, max = 50.0
Element prediction: Off
Number of isotope peaks used for i-FIT = 3

Monoisotopic Mass, Even Electron Ions
164 formula(e) evaluated with 2 results within limits (up to 20 best isotopic matches for each mass)
Elements Used:
C: 0-100 H: 0-200 N: 0-2 O: 0-100
79-1
2016092602 77 (0.632)
1: TOF MS ES+
5.25e+005

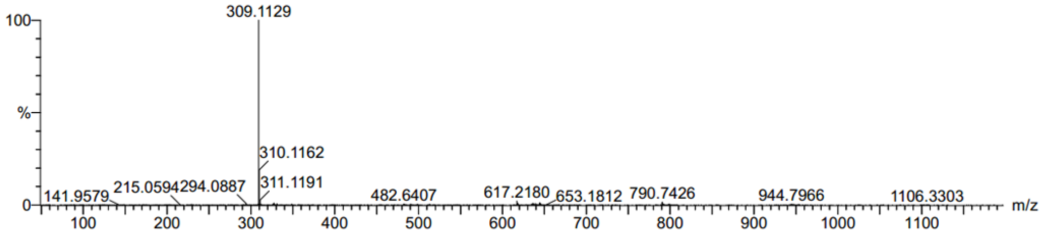

Minimum:				-1.5				
Maximum:		5.0	10.0	50.0				
Mass	Calc. Mass	mDa	PPM	DBE	i-FIT	Norm	Conf(%)	Formula
309.1129	309.1127	0.2	0.6	11.5	353.7	0.000	100.00	C19 H17 O4

Supplementary Figure 24. The HRESIMS spectrum of 4.

The HRESIMS spectrum (positive) showed *m/z* 309.1129 $[M + H]^+$ (calcd. for $C_{19}H_{17}O_4$, 309.1127).

a

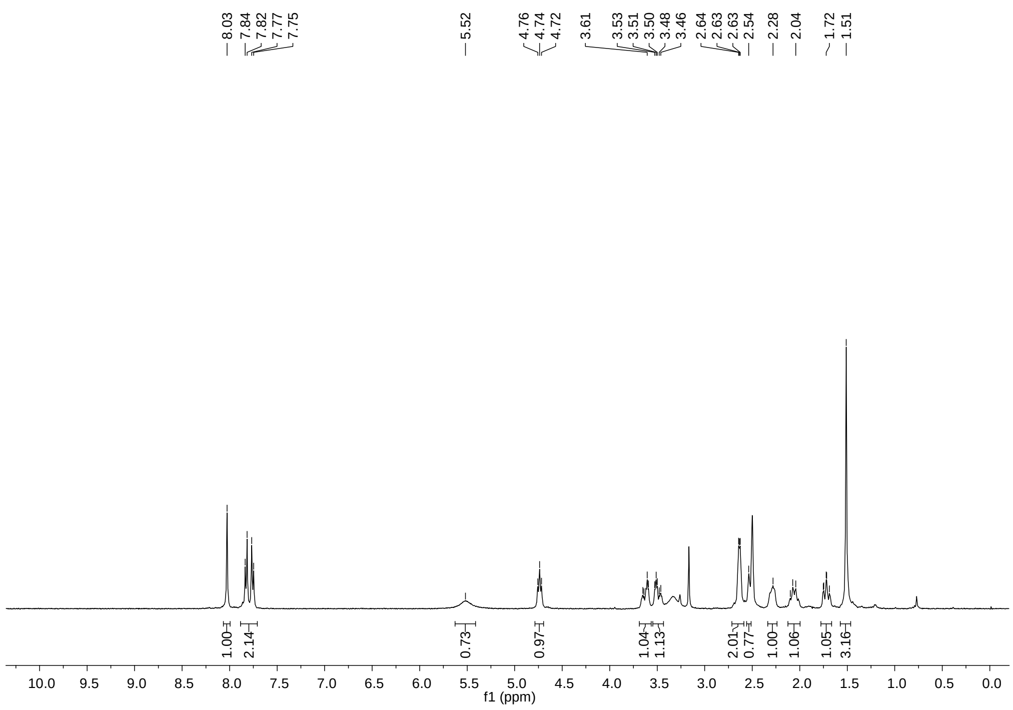

b

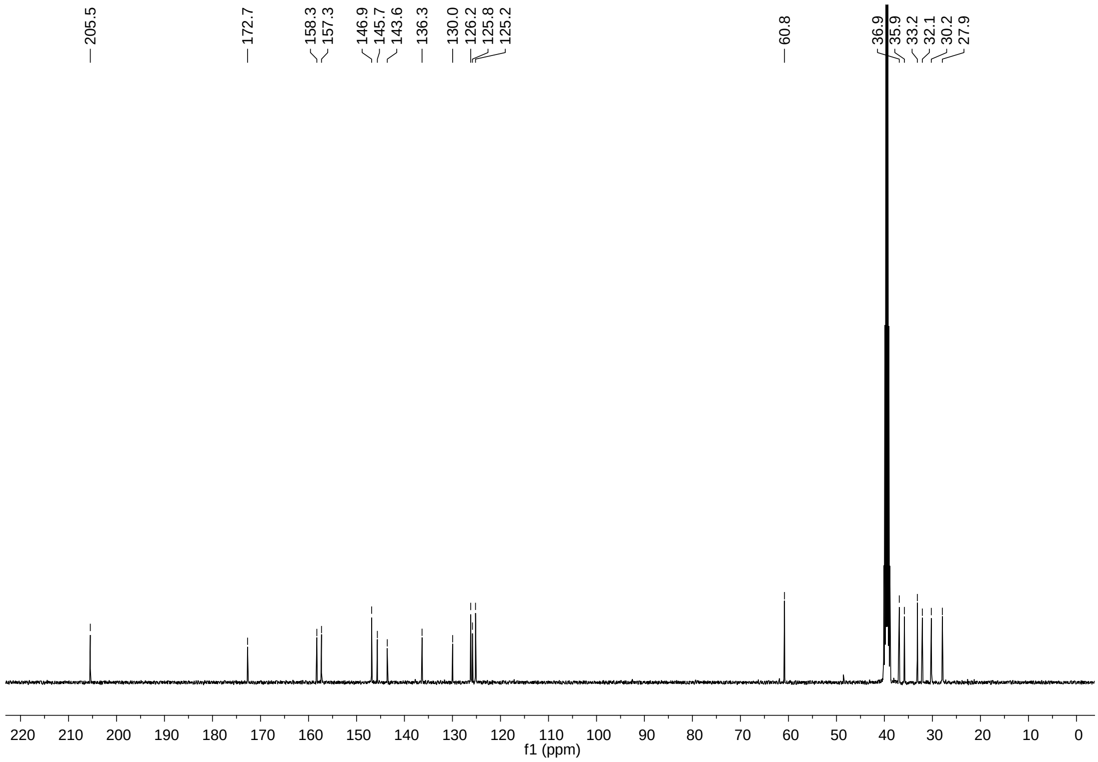

Supplementary Figure 25. The ^{1}H NMR and ^{13}C NMR spectra of 4.

(**a**) ^{1}H NMR spectrum in DMSO-d_6 at 400 MHz; (**b**) ^{13}C NMR spectrum in DMSO-d_6 at 100 MHz.

a

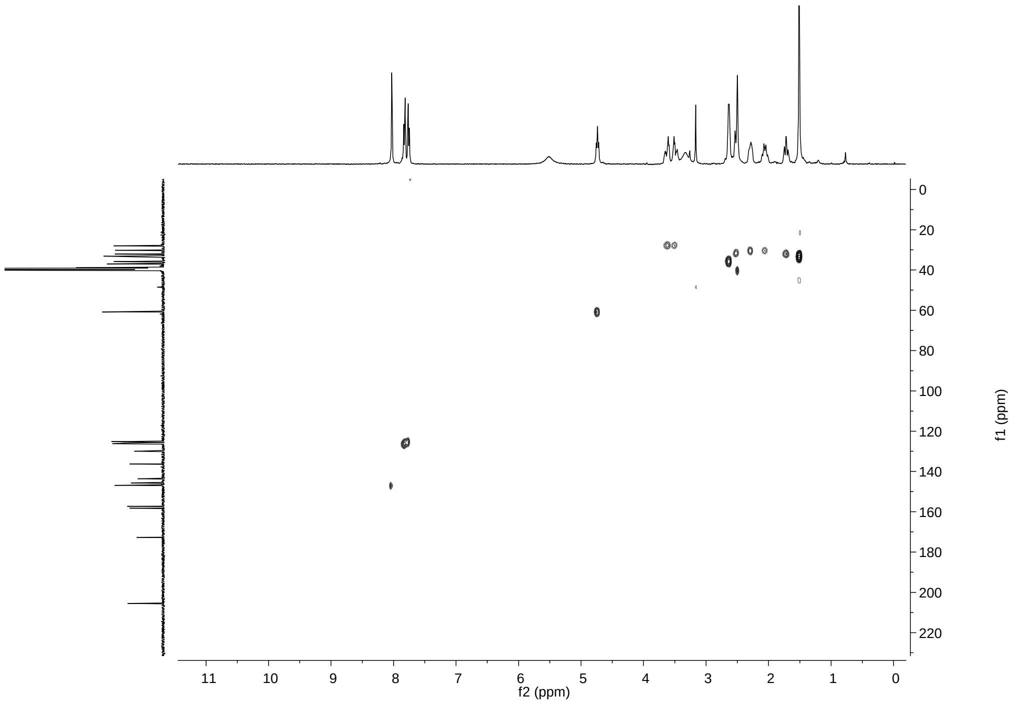

b

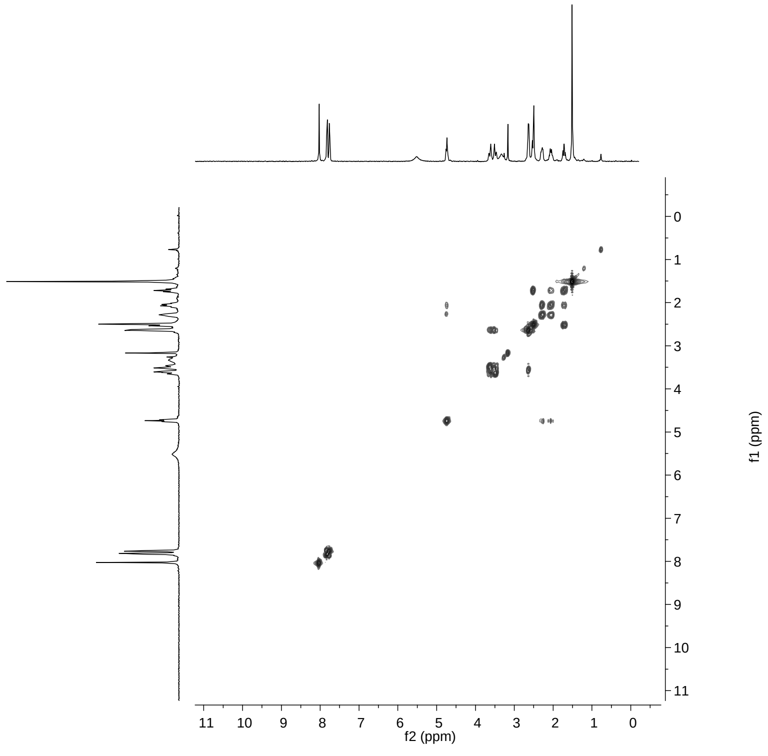

Supplementary Figure 26. The HSQC and ^{1}H-^{1}H COSY spectra of 4.

(**a**) HSQC spectrum in DMSO-d_6 at 400 MHz; (**b**) ^{1}H-^{1}H COSY spectrum in DMSO-d_6 at 400 MHz.

a

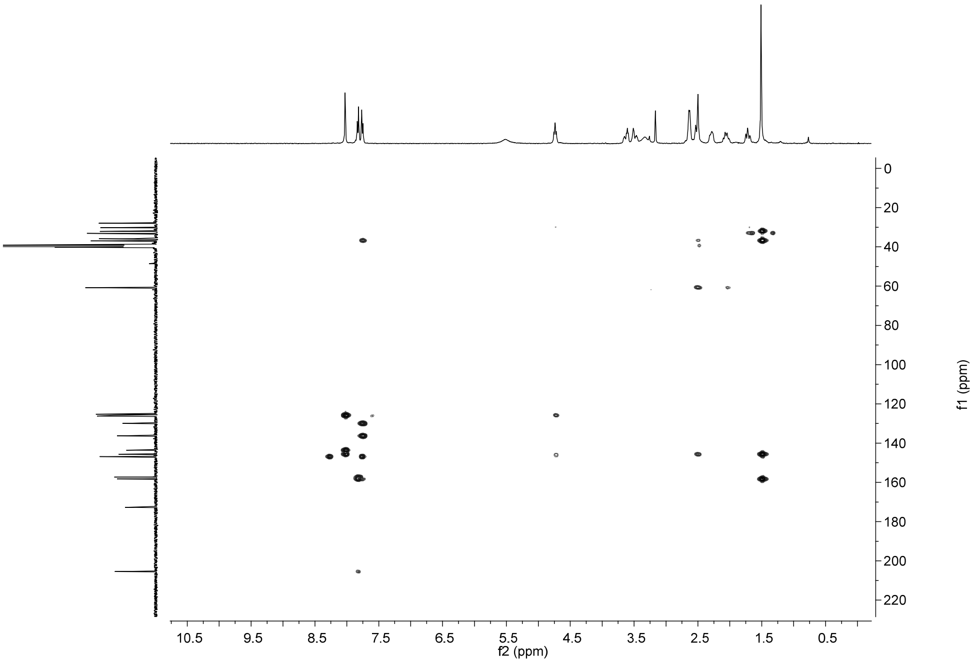

b

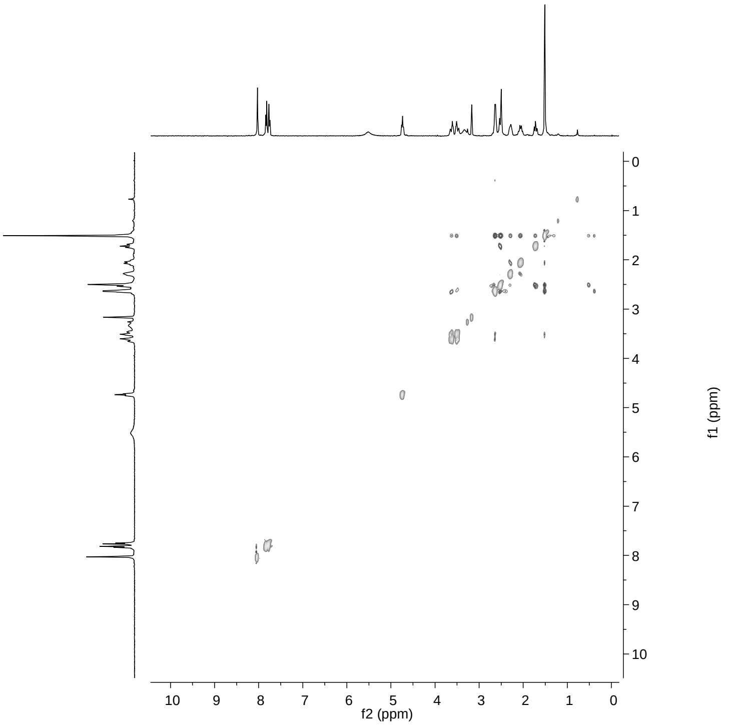

Supplementary Figure 27. The HMBC and ROESY spectra of 4.

(**a**) HMBC spectrum in DMSO-d_6 at 400 MHz; (**b**) ROESY spectrum in DMSO-d_6 at 400 MHz.

Single Mass Analysis
Tolerance = 10.0 PPM / DBE: min = -1.5, max = 50.0
Element prediction: Off
Number of isotope peaks used for i-FIT = 3

Monoisotopic Mass, Even Electron Ions
61 formula(e) evaluated with 1 results within limits (up to 20 best isotopic matches for each mass)
Elements Used:
C: 0-800 H: 0-200 O: 0-100
66-4-3
2016091210 98 (0.801)

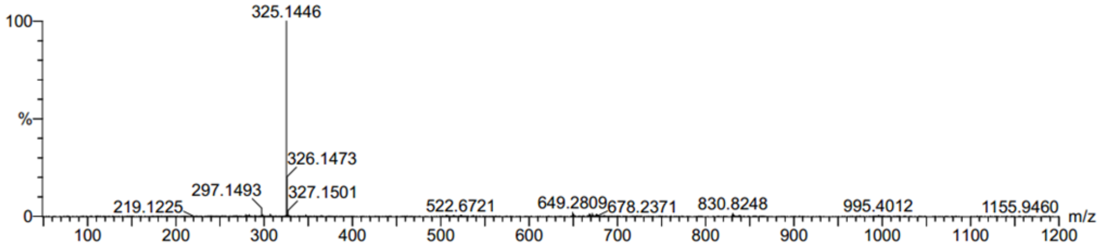

Minimum:				-1.5				
Maximum:		5.0	10.0	50.0				
Mass	Calc. Mass	mDa	PPM	DBE	i-FIT	Norm	Conf(%)	Formula
325.1446	325.1440	0.6	1.8	10.5	351.5	n/a	n/a	C20 H21 O4

Supplementary Figure 28. The HRESIMS spectrum of 5.

The HRESIMS spectrum (positive) showed *m/z* 325.1446 $[M + H]^+$ (calcd. for $C_{20}H_{21}O_4$, 325.1440).

a

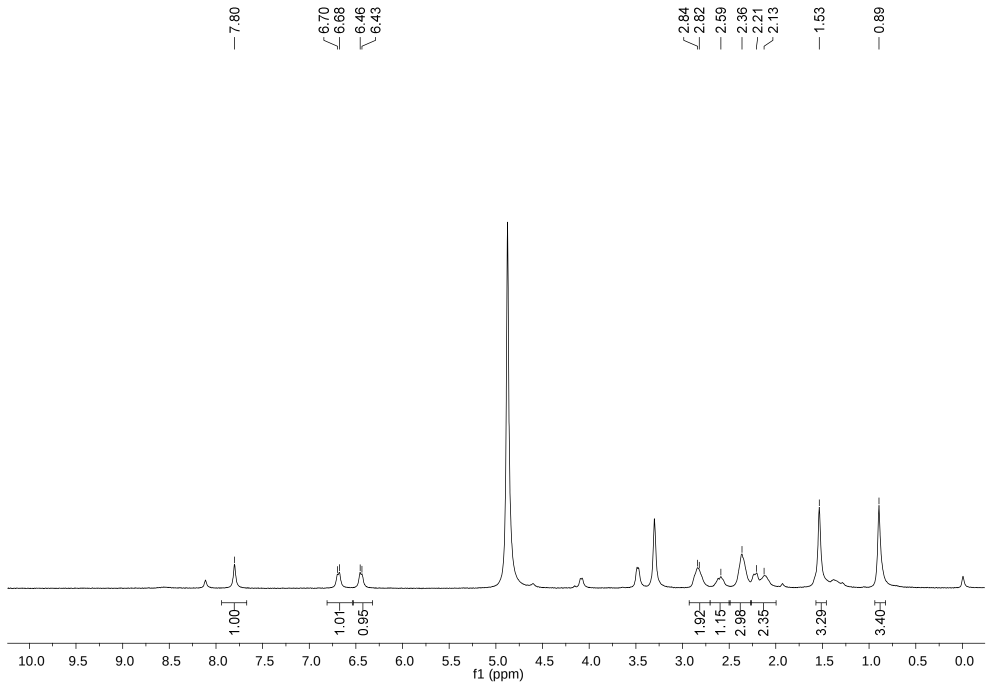

b

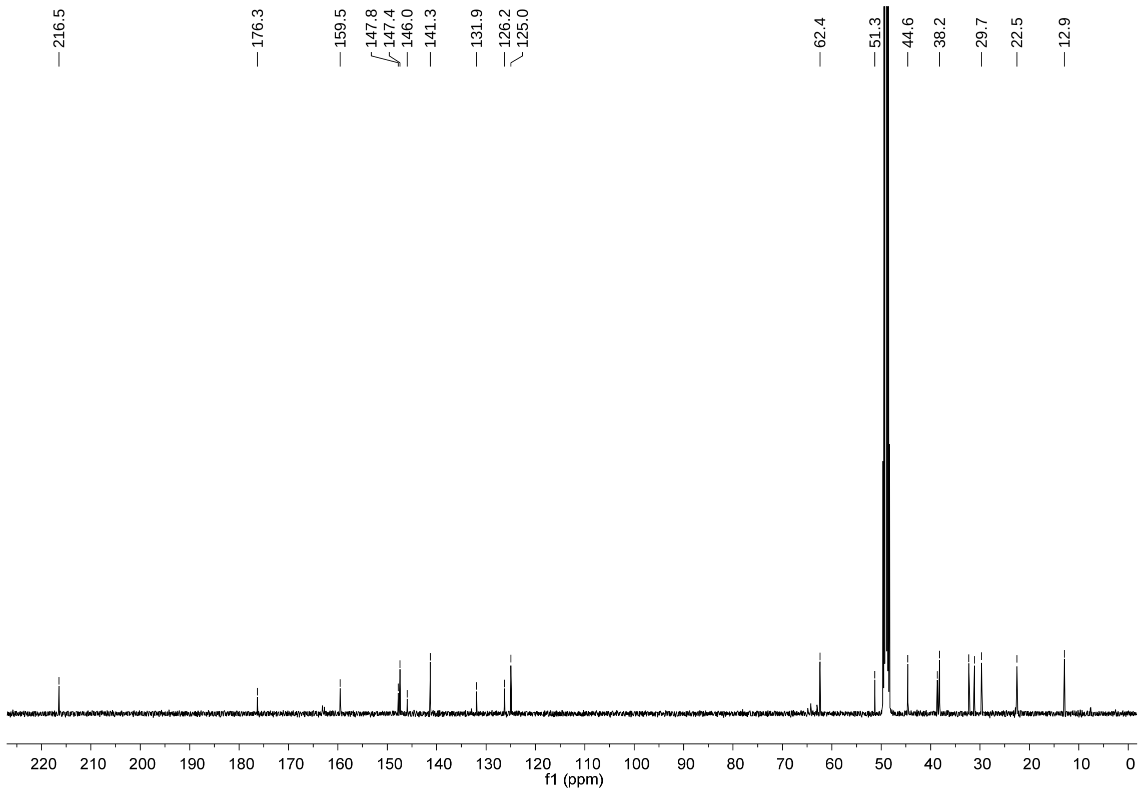

Supplementary Figure 29. The ^{1}H NMR and ^{13}C NMR spectra of 5.

(**a**) ^{1}H NMR spectrum in CD_3OD at 400 MHz; (**b**) ^{13}C NMR spectrum in CD_3OD at 100 MHz.

a

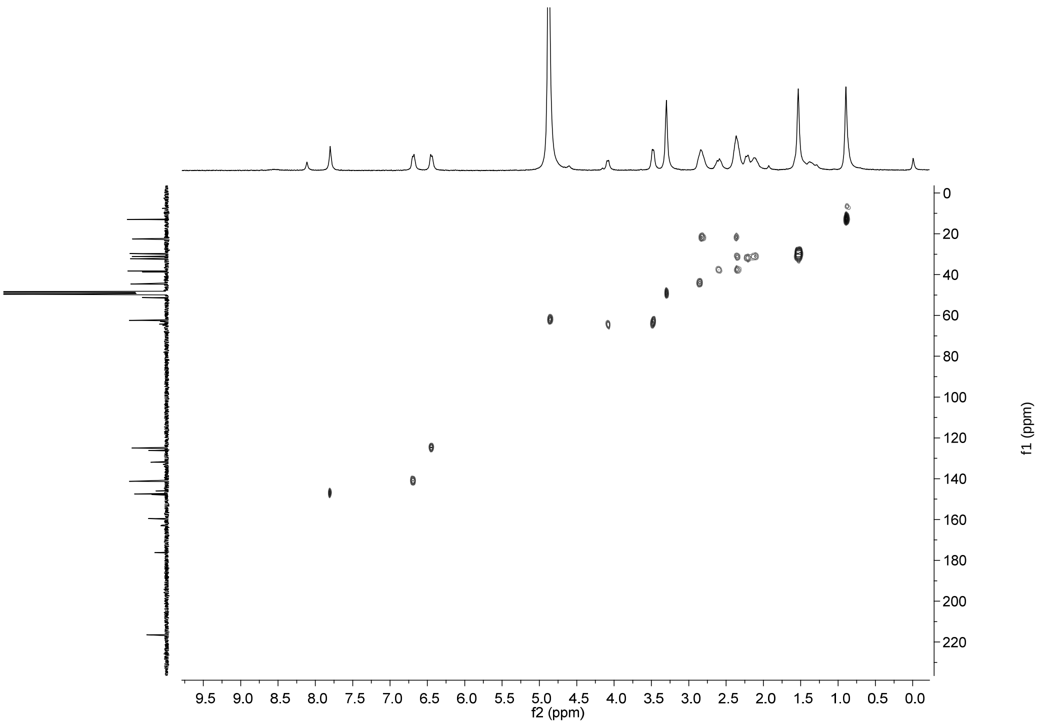

b

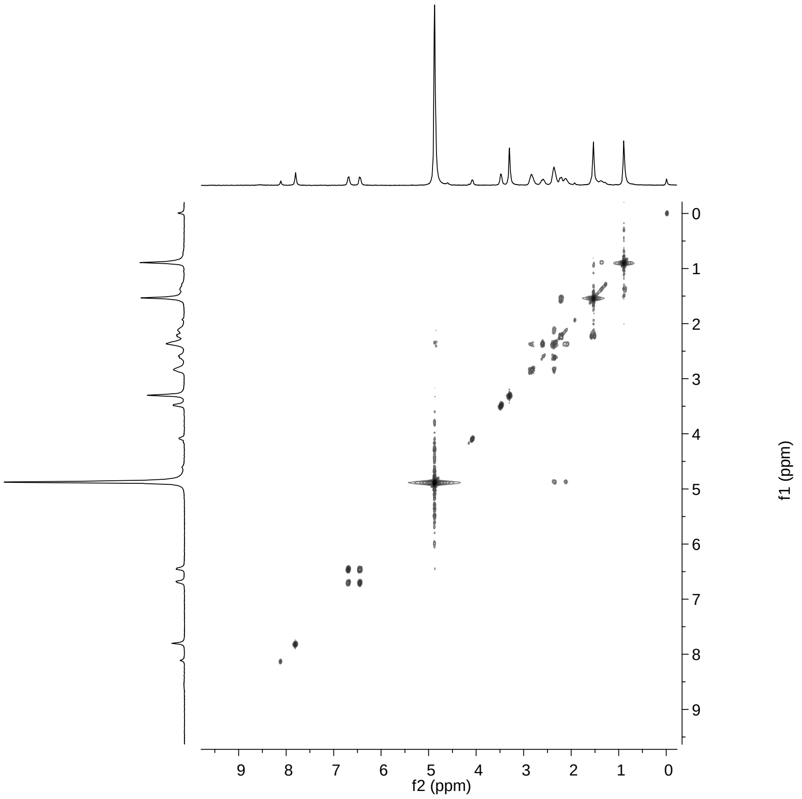

Supplementary Figure 30. The HSQC and ^{1}H-^{1}H COSY spectra of 5.

(**a**) HSQC spectrum in CD_3OD at 400 MHz; (**b**) ^{1}H-^{1}H COSY spectrum in CD_3OD at 400 MHz.

a

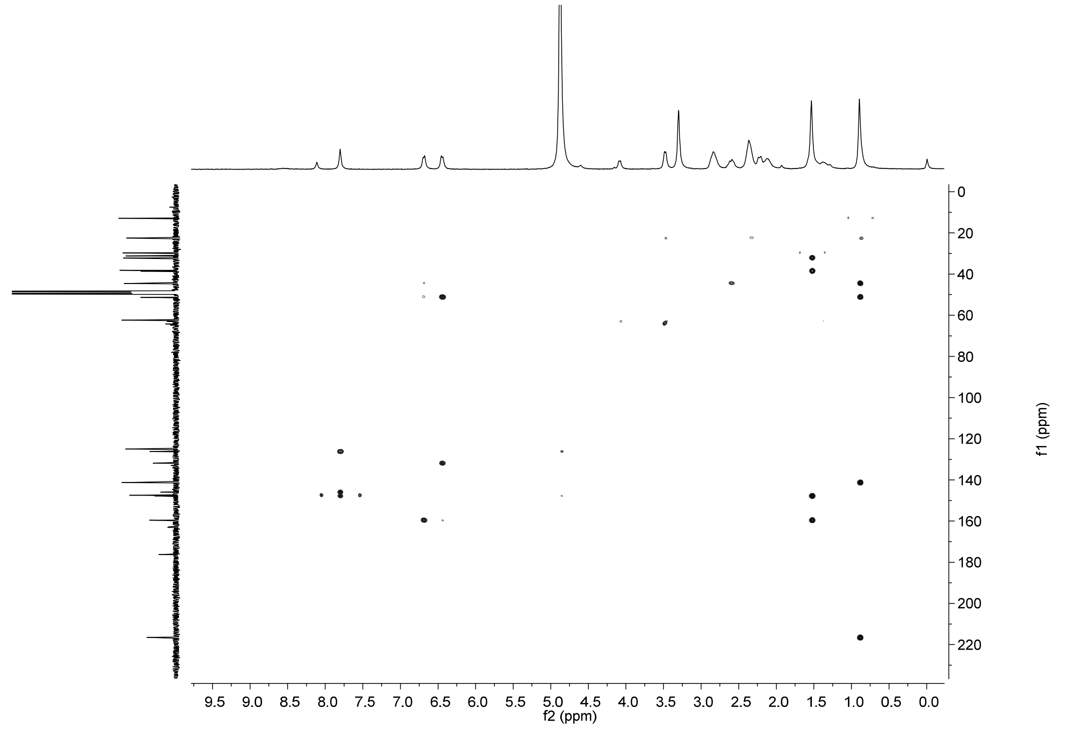

b

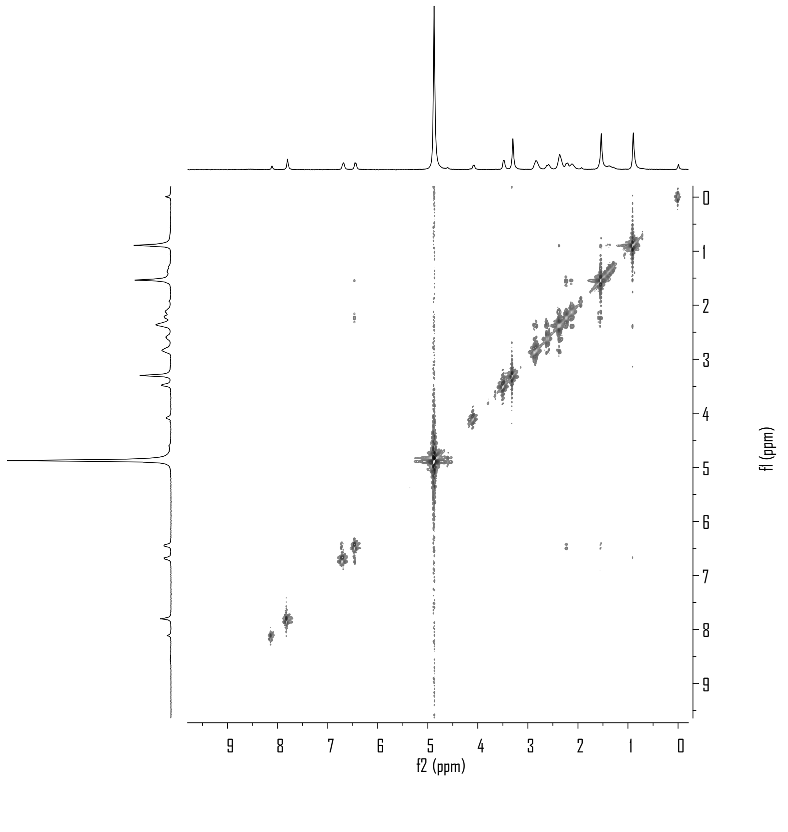

Supplementary Figure 31. The HMBC and NOESY spectra of 5.

(**a**) HMBC spectrum in CD_3OD at 400 MHz; (**b**) NOESY spectrum in CD_3OD at 400 MHz.

Single Mass Analysis
Tolerance = 10.0 PPM / DBE: min = -1.5, max = 50.0
Element prediction: Off
Number of isotope peaks used for i-FIT = 3

Monoisotopic Mass, Even Electron Ions
65 formula(e) evaluated with 1 results within limits (up to 20 best isotopic matches for each mass)
Elements Used:
C: 0-800 H: 0-200 O: 0-100
66-4-1-1
2016091207 76 (0.625) 1: TOF MS ES+
4.50e+005

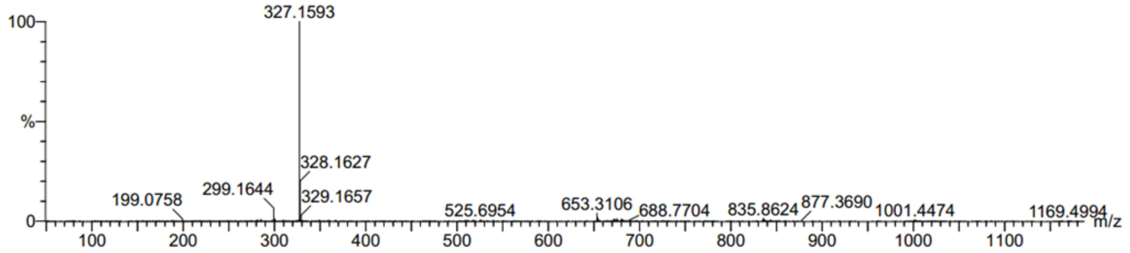

	Mass	Calc. Mass	mDa	PPM	DBE	i-FIT	Norm	Conf(%)	Formula
Minimum:					-1.5				
Maximum:			5.0	10.0	50.0				
	327.1593	327.1596	-0.3	-0.9	9.5	325.5	n/a	n/a	C20 H23 O4

Supplementary Figure 32. The HRESIMS spectrum of 6.

The HRESIMS spectrum (positive) showed *m/z* 327.1593 $[M + H]^+$ (calcd. for $C_{20}H_{23}O_4$, 327.1596).

a

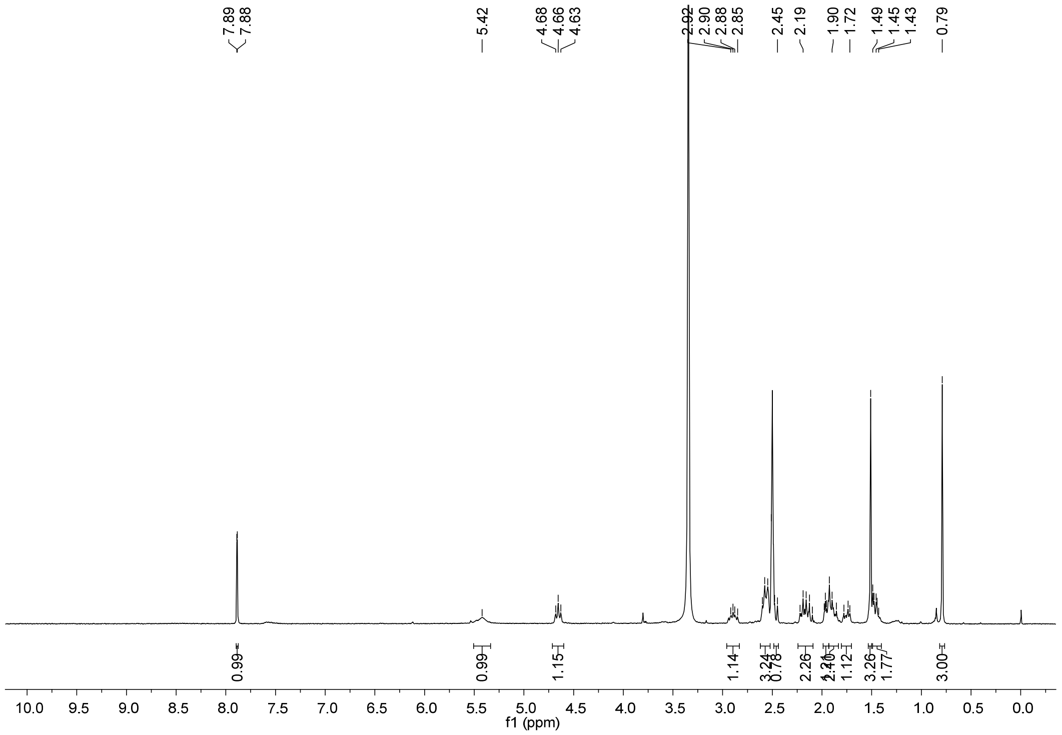

b

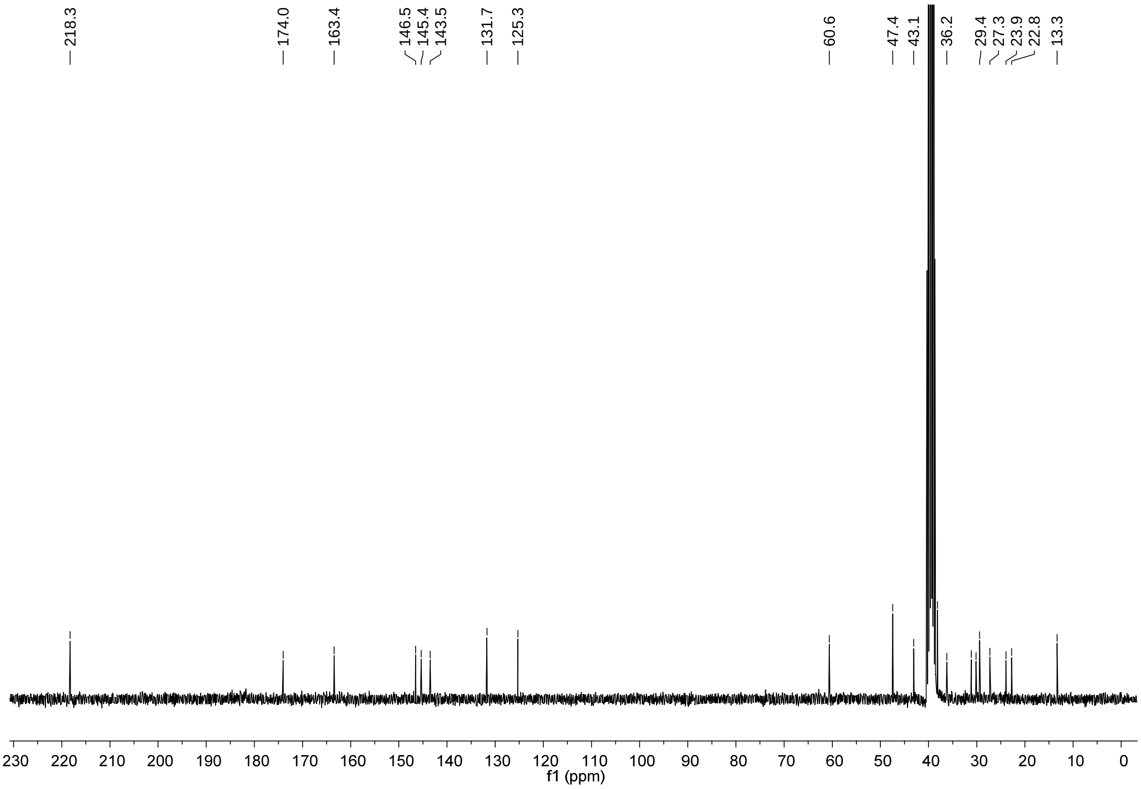

Supplementary Figure 33. The ^{1}H NMR and ^{13}C NMR spectra of 6.

(**a**) ^{1}H NMR spectrum in DMSO-d_6 at 300 MHz; (**b**) ^{13}C NMR spectrum in DMSO-d_6 at 75 MHz.

a

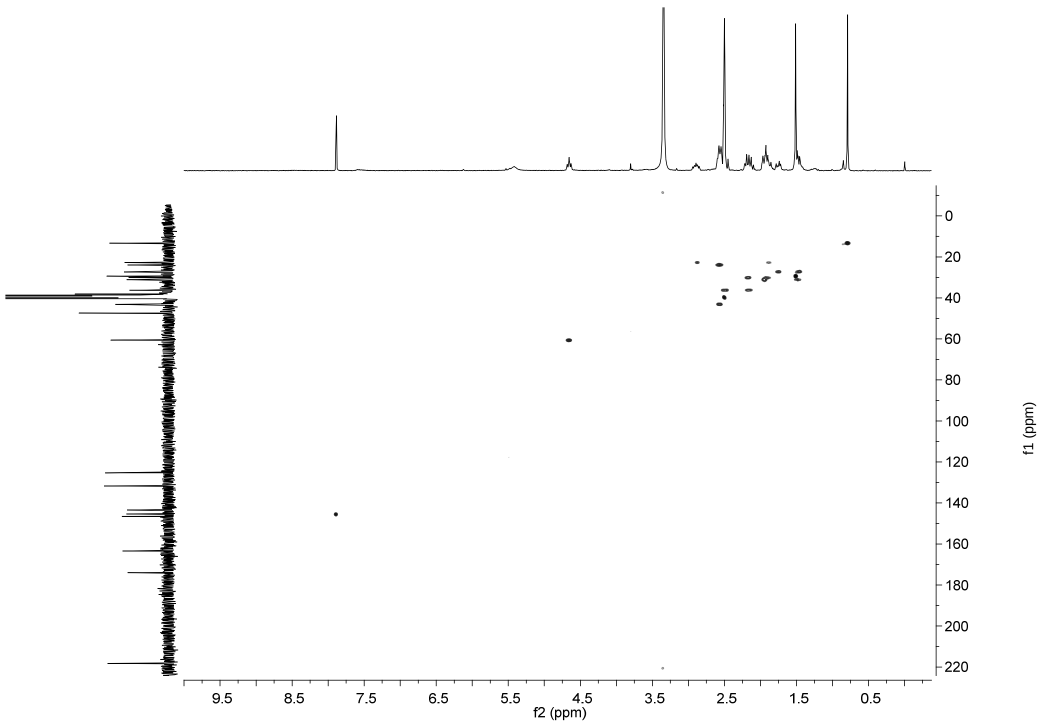

b

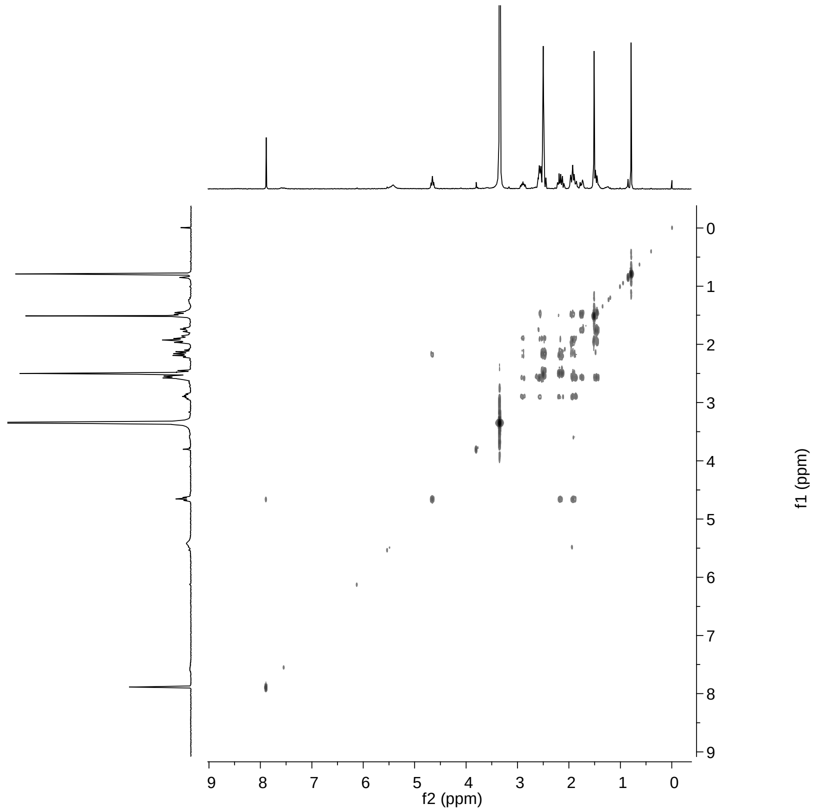

Supplementary Figure 34. The HSQC and ^{1}H-^{1}H COSY spectra of 6.

(**a**) HSQC spectrum in DMSO-d_6 at 400 MHz; (**b**) ^{1}H-^{1}H COSY spectrum in DMSO-d_6 at 400 MHz.

a

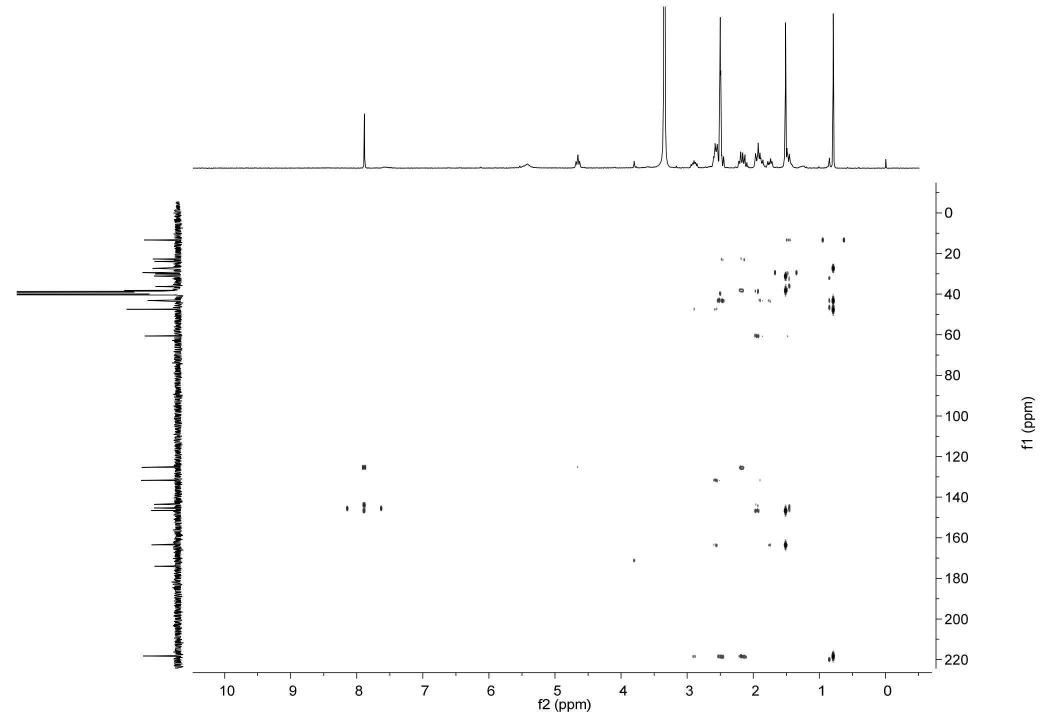

b

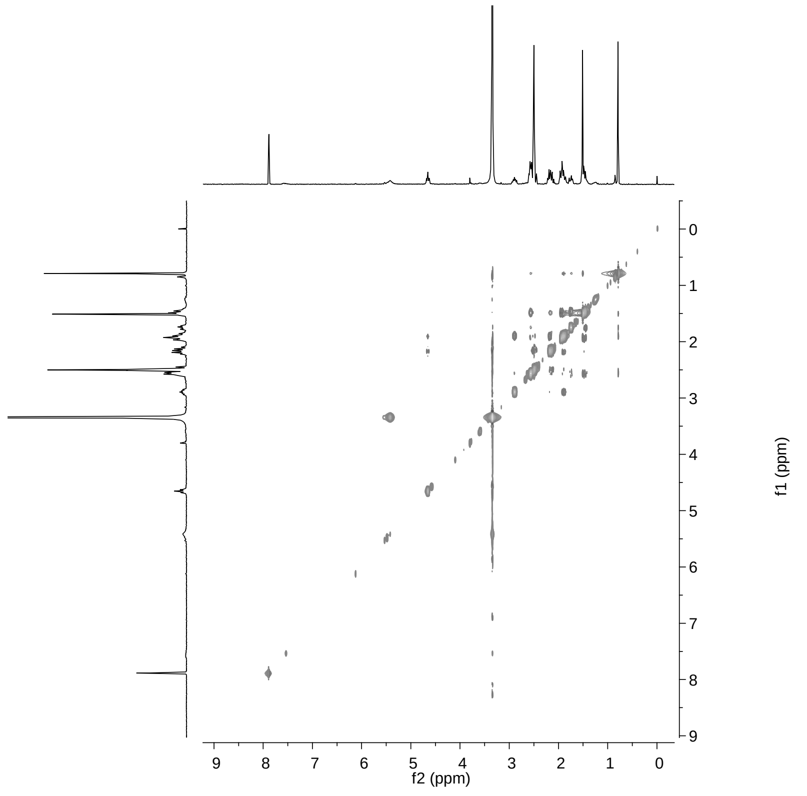

Supplementary Figure 35. The HMBC and ROESY spectra of 6.

(**a**) HMBC spectrum in DMSO-d_6 at 400 MHz; (**b**) ROESY spectrum in DMSO-d_6 at 400 MHz.

Single Mass Analysis
Tolerance = 5.0 mDa / DBE: min = -1.5, max = 50.0
Element prediction: Off
Number of isotope peaks used for i-FIT = 3

Monoisotopic Mass, Even Electron Ions
65 formula(e) evaluated with 1 results within limits (up to 50 best isotopic matches for each mass)
Elements Used:
C: 0-500 H: 0-1000 O: 0-200
3274-3
2016112115 91 (0.748)

1: TOF MS ES+
2.09e+004

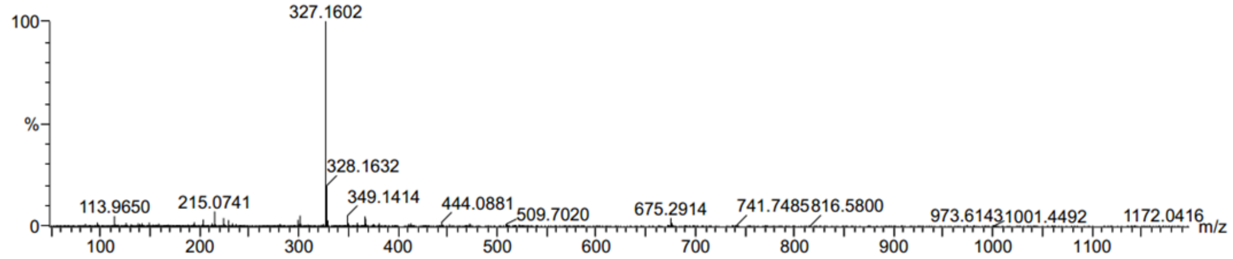

Minimum:				-1.5				
Maximum:		5.0	10.0	50.0				

Mass	Calc. Mass	mDa	PPM	DBE	i-FIT	Norm	Conf(%)	Formula
327.1602	327.1596	0.6	1.8	9.5	36.9	n/a	n/a	C20 H23 O4

Supplementary Figure 36. The HRESIMS spectrum of 7.

The HRESIMS spectrum (positive) showed *m/z* 327.1602 $[M + H]^+$ (calcd. for $C_{20}H_{23}O_4$, 327.1596).

a

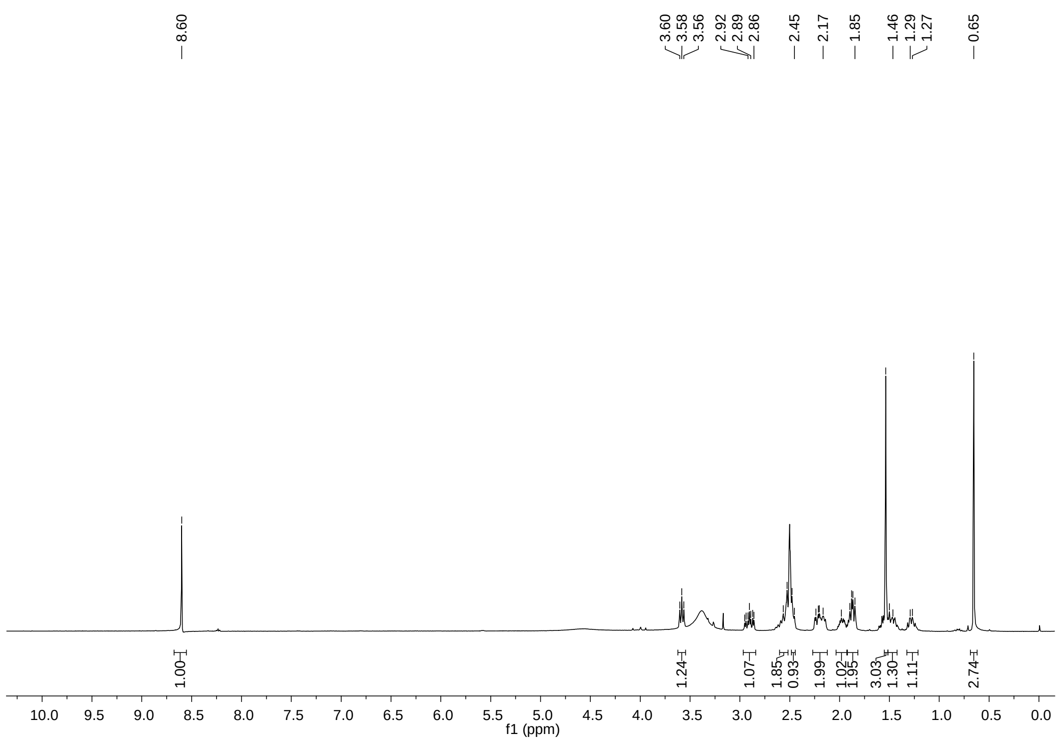

b

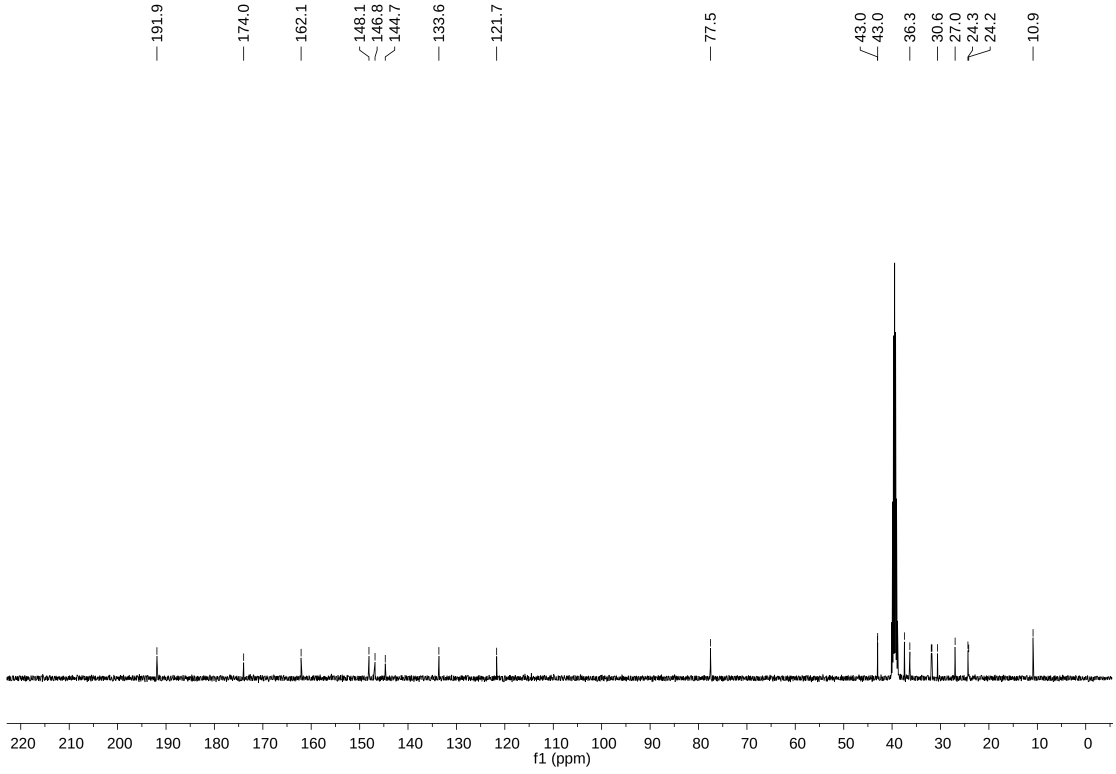

Supplementary Figure 37. The ^{1}H NMR and ^{13}C NMR spectra of 7.

(**a**) ^{1}H NMR spectrum in DMSO-d_6 at 400 MHz; (**b**) ^{13}C NMR spectrum in DMSO-d_6 at 100 MHz.

a

b

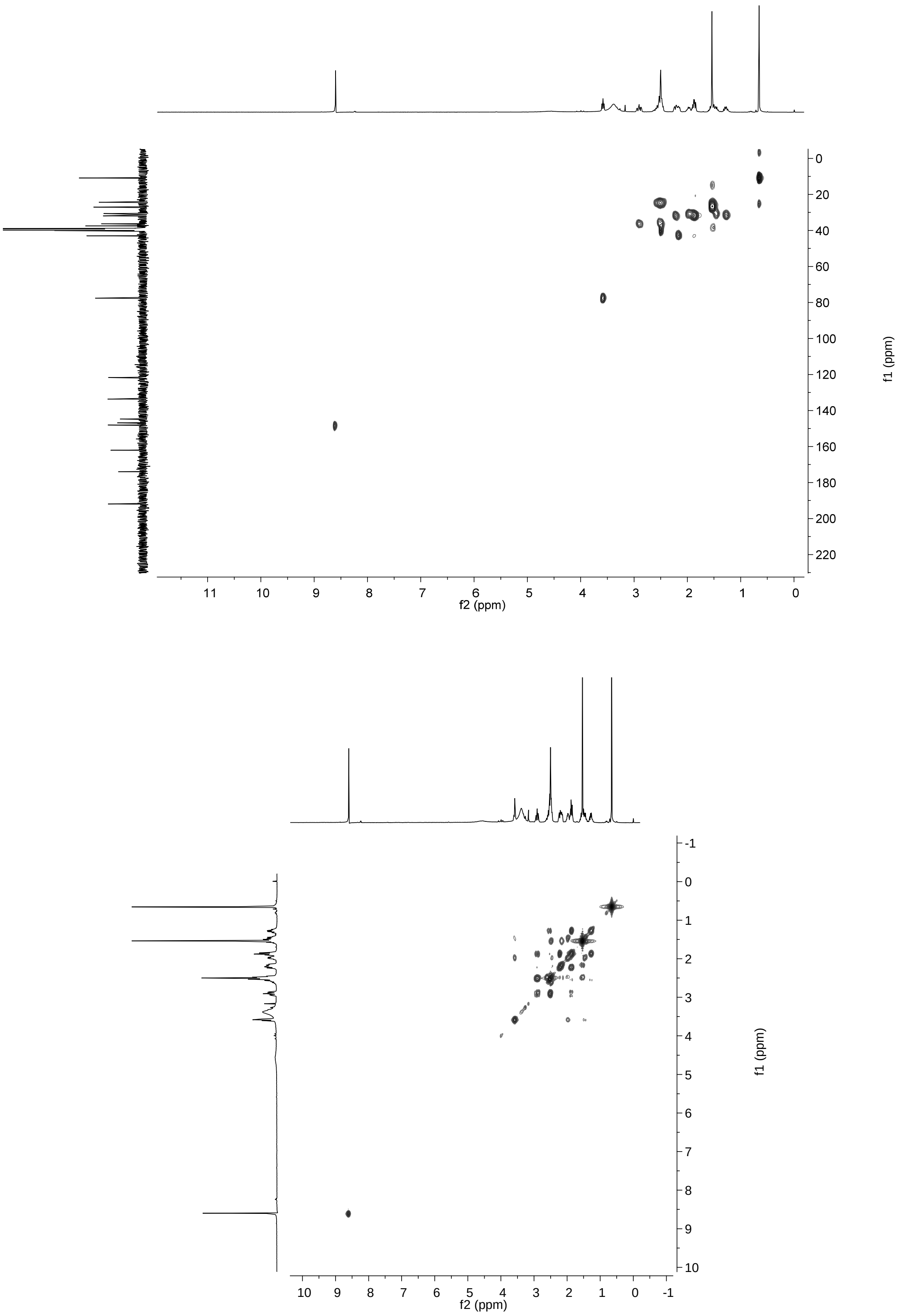

Supplementary Figure 38. The HSQC and ^{1}H-^{1}H COSY spectra of 7.

(**a**) HSQC spectrum in DMSO-d_6 at 400 MHz; (**b**) ^{1}H-^{1}H COSY spectrum in DMSO-d_6 at 400 MHz.

a

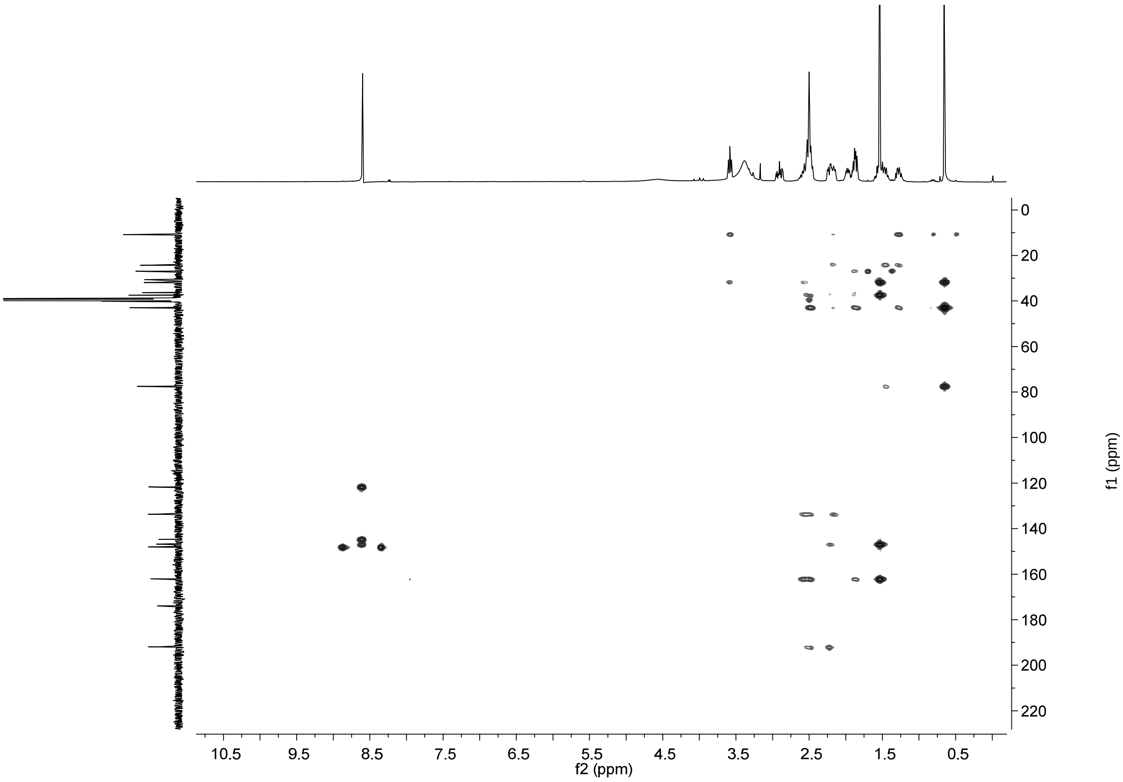

b

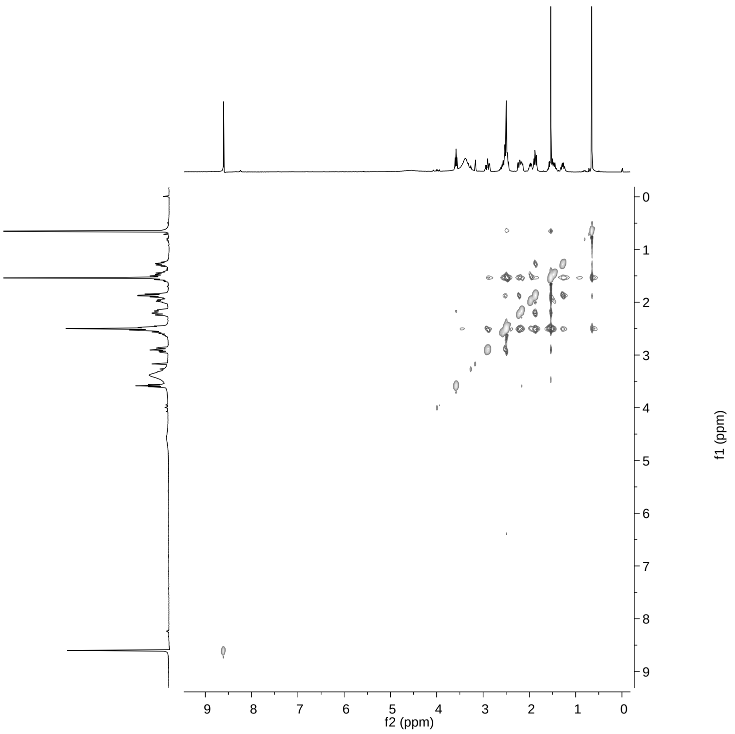

Supplementary Figure 39. The HMBC and ROESY spectra of 7.

(**a**) HMBC spectrum in DMSO-d_6 at 400 MHz; (**b**) ROESY spectrum in DMSO-d_6 at 400 MHz.

Single Mass Analysis
Tolerance = 5.0 mDa / DBE: min = -1.5, max = 50.0
Element prediction: Off
Number of isotope peaks used for i-FIT = 3

Monoisotopic Mass, Even Electron Ions
115 formula(e) evaluated with 2 results within limits (up to 50 best isotopic matches for each mass)
Elements Used:
C: 0-500 H: 0-1000 O: 0-200 Na: 0-1
69-3
2016101010 48 (0.404) Cm (48:49)

1: TOF MS ES+
2.74e+005

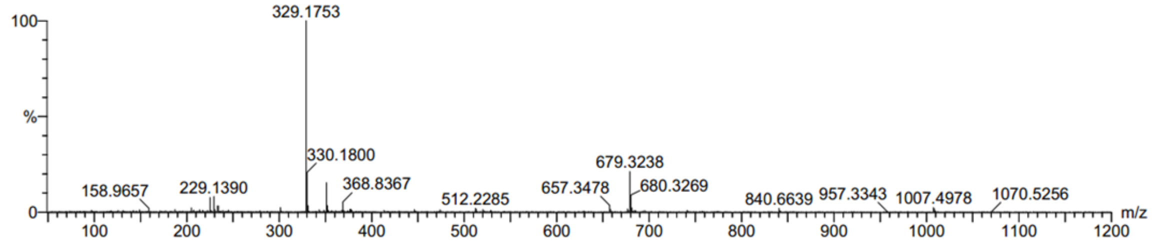

Minimum:				-1.5				
Maximum:		5.0	10.0	50.0				
Mass	Calc. Mass	mDa	PPM	DBE	i-FIT	Norm	Conf(%)	Formula
329.1753	329.1753	0.0	0.0	8.5	19.2	0.286	75.12	C20 H25 O4

Supplementary Figure 40. The HRESIMS spectrum of 8.

The HRESIMS spectrum (positive) showed *m/z* 329.1753 $[M + H]^+$ (calcd. for $C_{20}H_{25}O_4$, 329.1753).

a

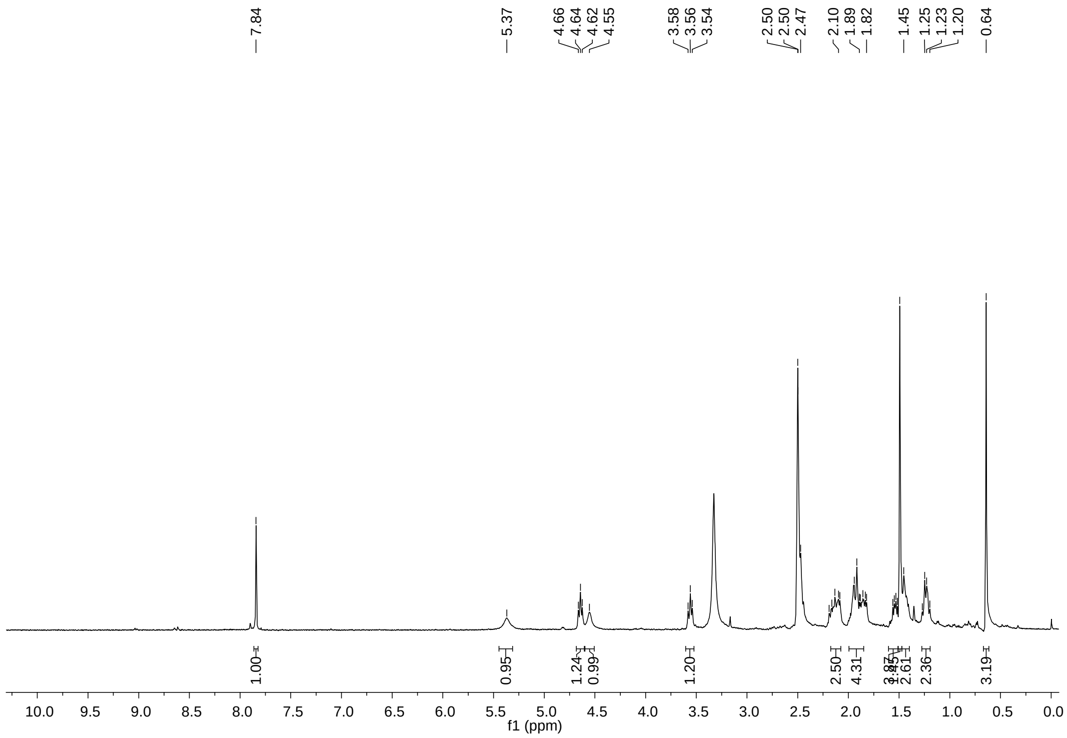

b

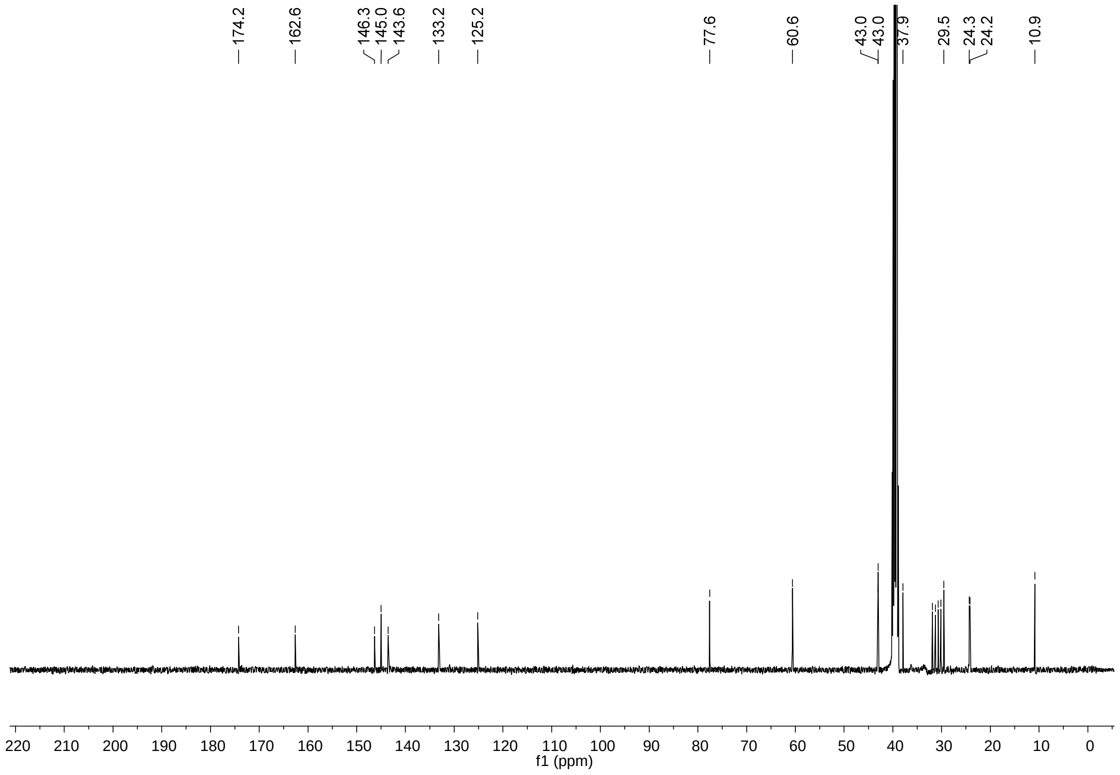

Supplementary Figure 41. The ^{1}H NMR and ^{13}C NMR spectra of 8.

(**a**) ^{1}H NMR spectrum in DMSO-d_6 at 400 MHz; (**b**) ^{13}C NMR spectrum in DMSO-d_6 at 100 MHz.

a

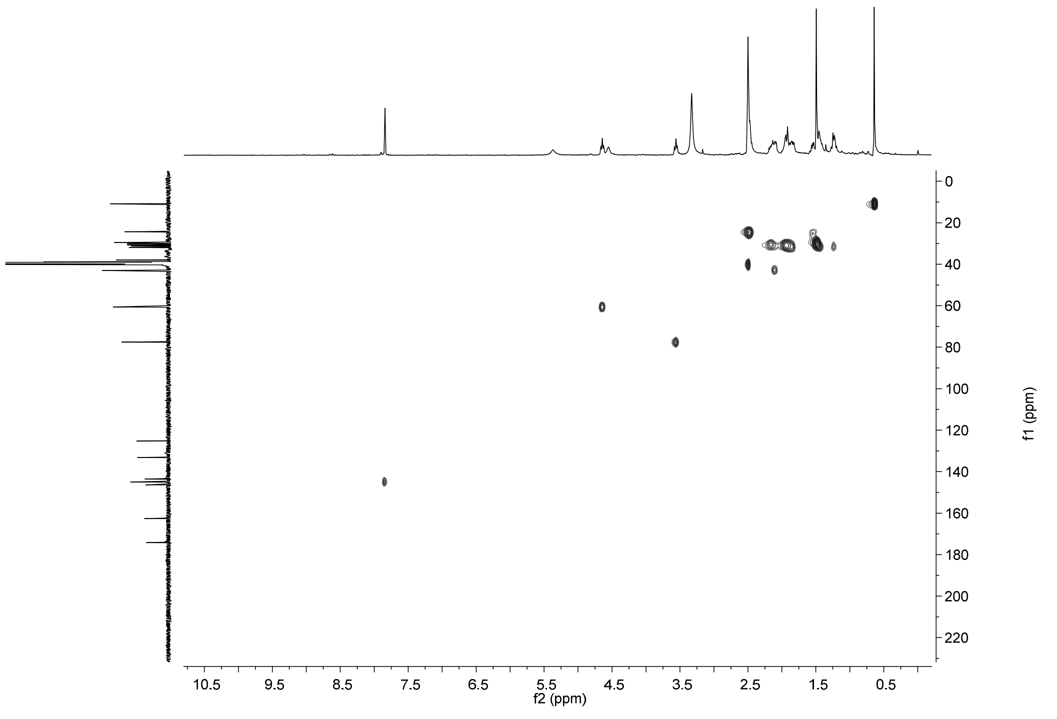

b

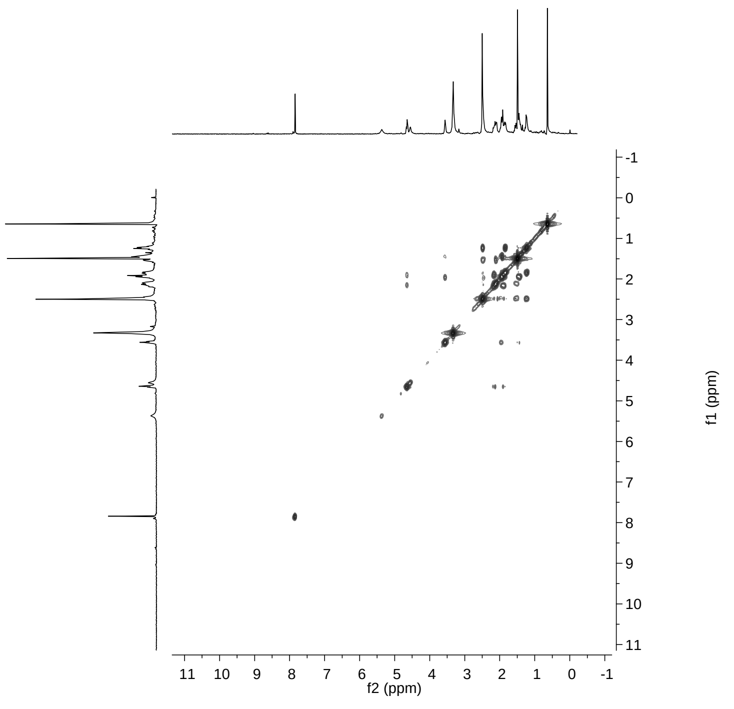

Supplementary Figure 42. The HSQC and ^{1}H-^{1}H COSY spectra of 8.

(**a**) HSQC spectrum in DMSO-d_6 at 400 MHz; (**b**) ^{1}H-^{1}H COSY spectrum in DMSO-d_6 at 400 MHz.

a

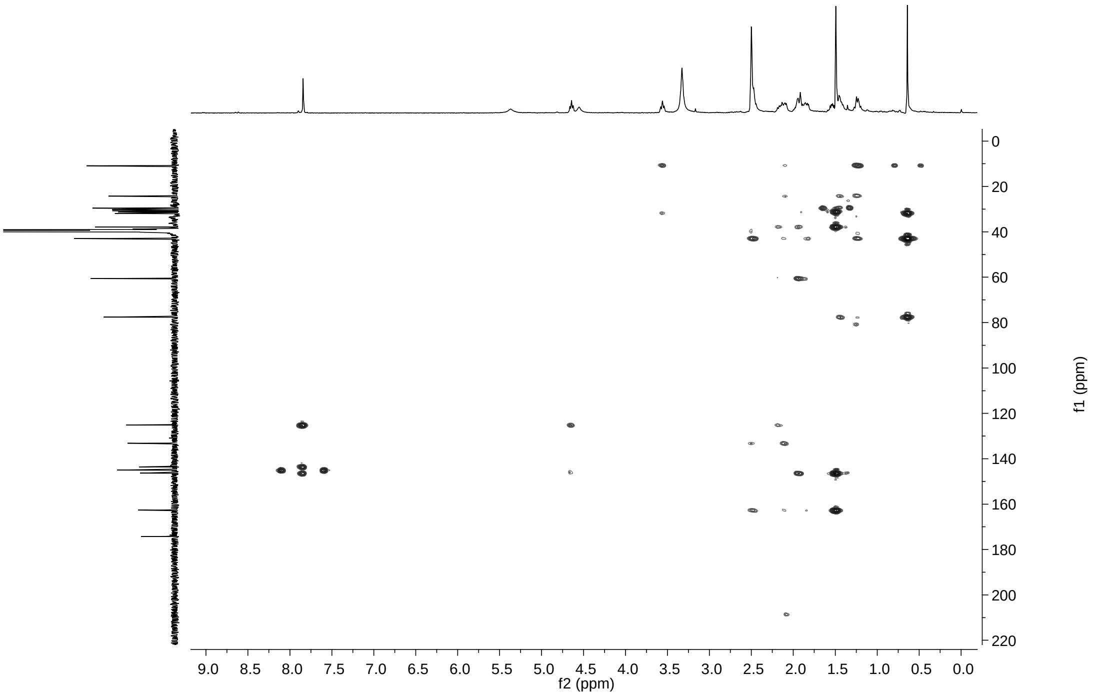

b

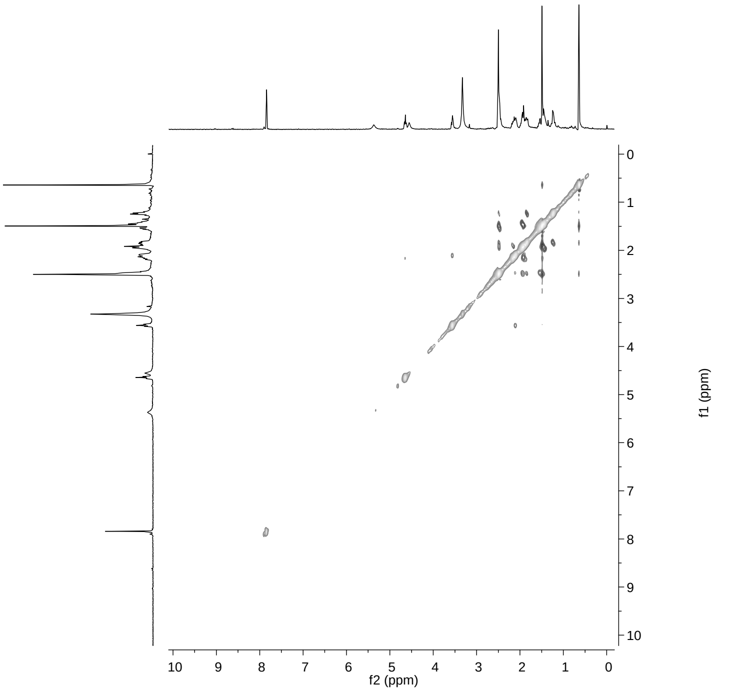

Supplementary Figure 43. The HMBC and ROESY spectra of 8.

(**a**) HMBC spectrum in DMSO-d_6 at 400 MHz; (**b**) ROESY spectrum in DMSO-d_6 at 400 MHz.

Single Mass Analysis
Tolerance = 10.0 PPM / DBE: min = -1.5, max = 50.0
Element prediction: Off
Number of isotope peaks used for i-FIT = 3

Monoisotopic Mass, Even Electron Ions
148 formula(e) evaluated with 2 results within limits (up to 50 closest results for each mass)
Elements Used:
C: 0-80 H: 0-200 O: 0-50 23Na: 0-1
85-4-1
2017010202 157 (1.276)

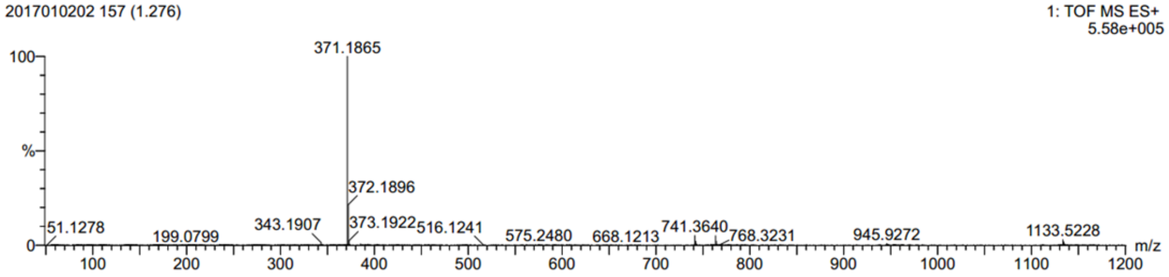

Minimum:				-1.5				
Maximum:		5.0	10.0	50.0				
Mass	Calc. Mass	mDa	PPM	DBE	i-FIT	Norm	Conf(%)	Formula
371.1865	371.1858	0.7	1.9	9.5	260.6	0.612	54.20	C22 H27 O5

Supplementary Figure 44. The HRESIMS spectrum of 9.

The HRESIMS spectrum (positive) showed *m/z* 371.1865 $[M + H]^+$ (calcd. for $C_{22}H_{27}O_5$, 371.1858).

a

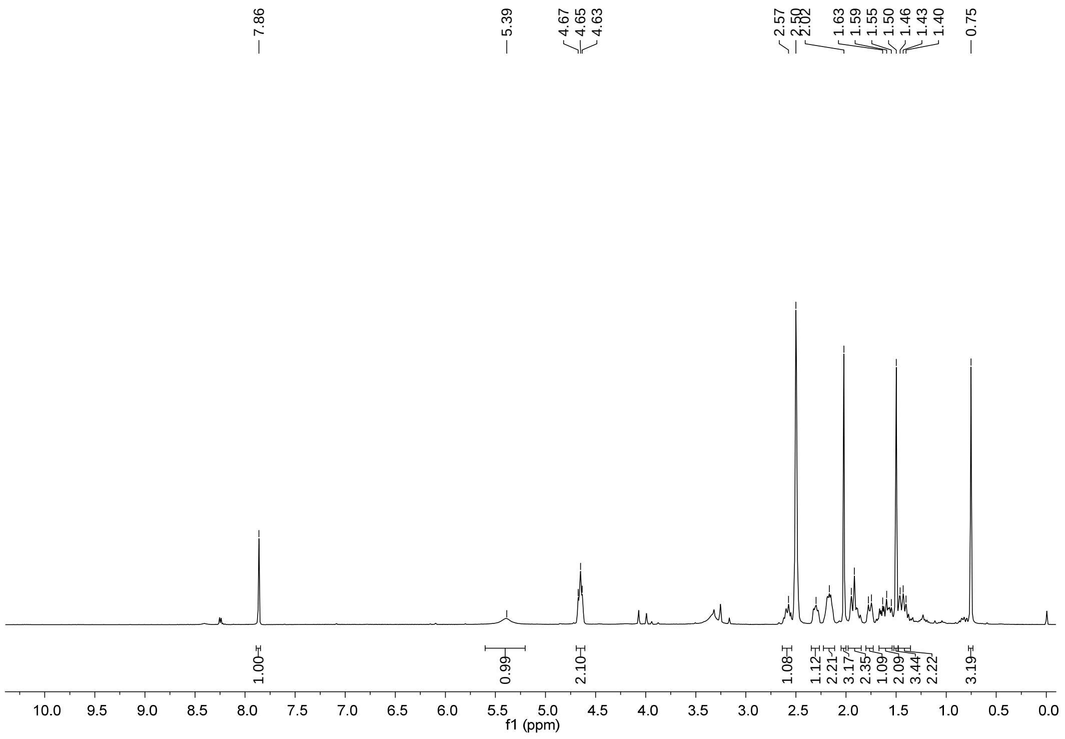

b

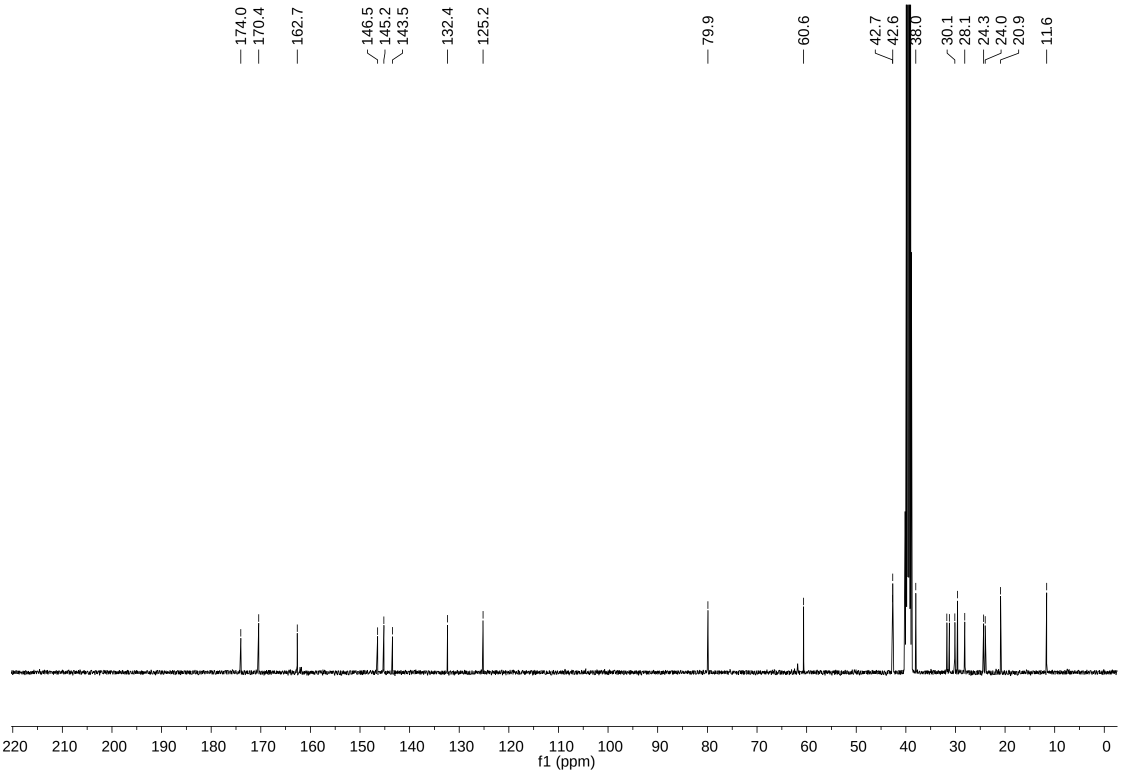

Supplementary Figure 45. The ^{1}H NMR and ^{13}C NMR spectra of 9.

(**a**) ^{1}H NMR spectrum in DMSO-d_6 at 400 MHz; (**b**) ^{13}C NMR spectrum in DMSO-d_6 at 100 MHz.

a

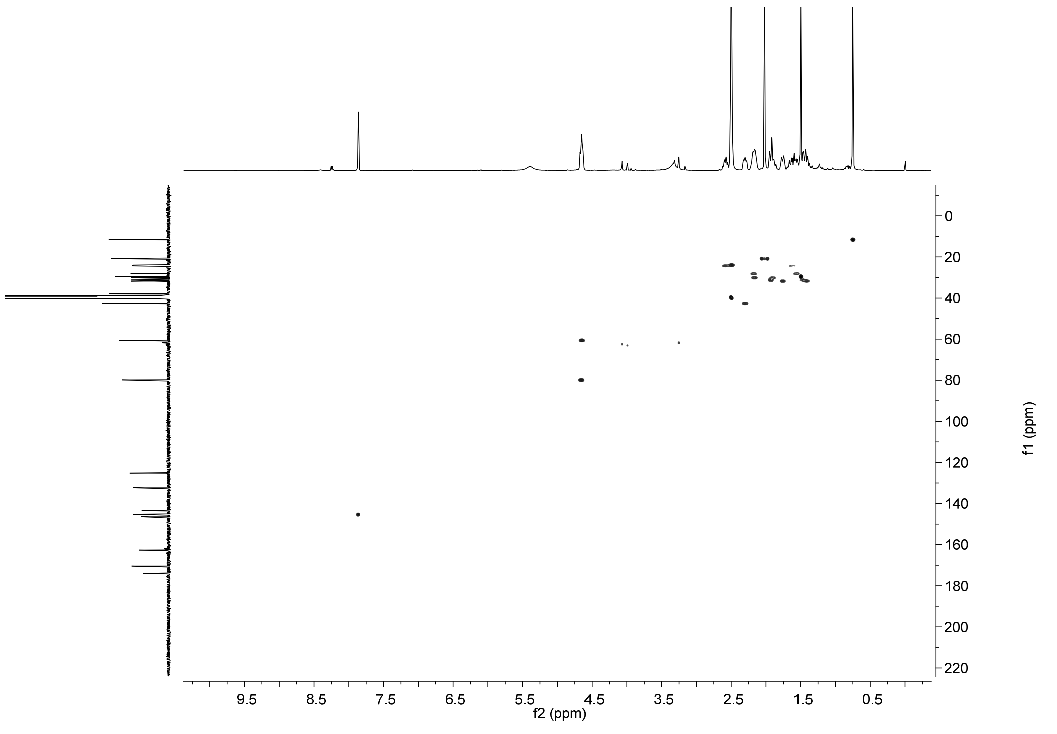

b

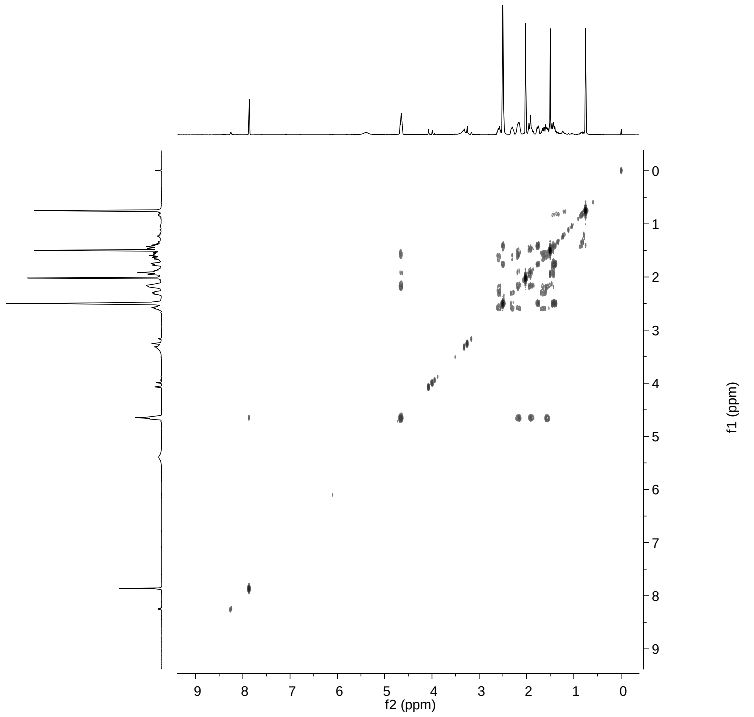

Supplementary Figure 46. The HSQC and ^{1}H-^{1}H COSY spectra of 9.

(**a**) HSQC spectrum in DMSO-d_6 at 400 MHz; (**b**) ^{1}H-^{1}H COSY spectrum in DMSO-d_6 at 400 MHz.

a

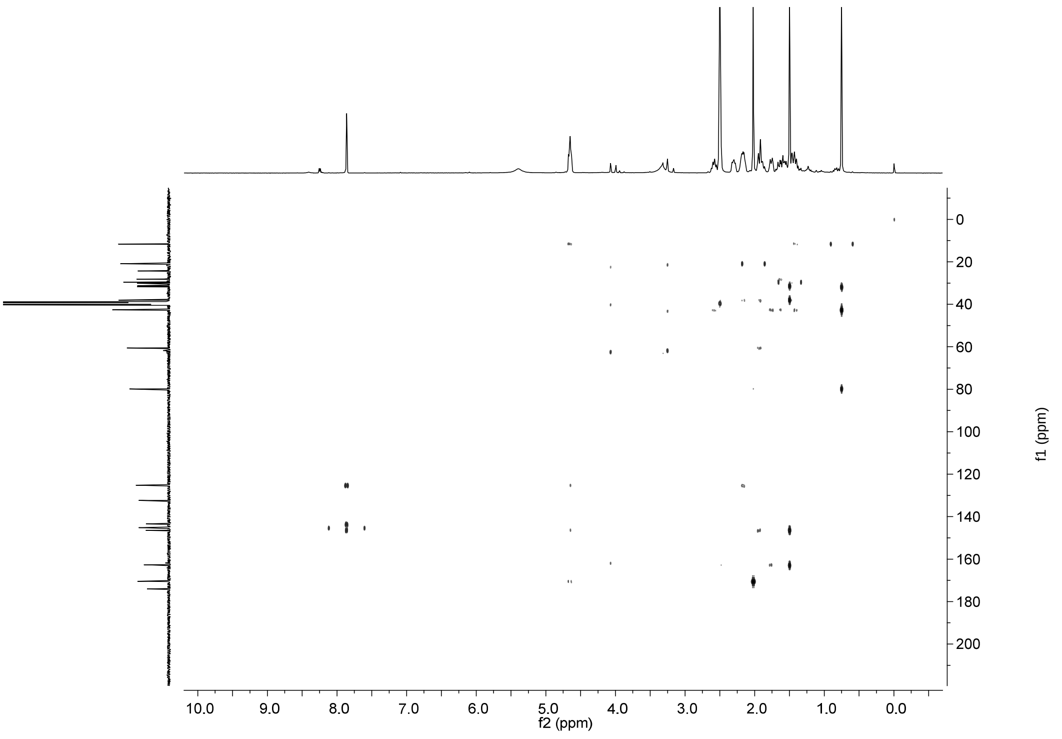

b

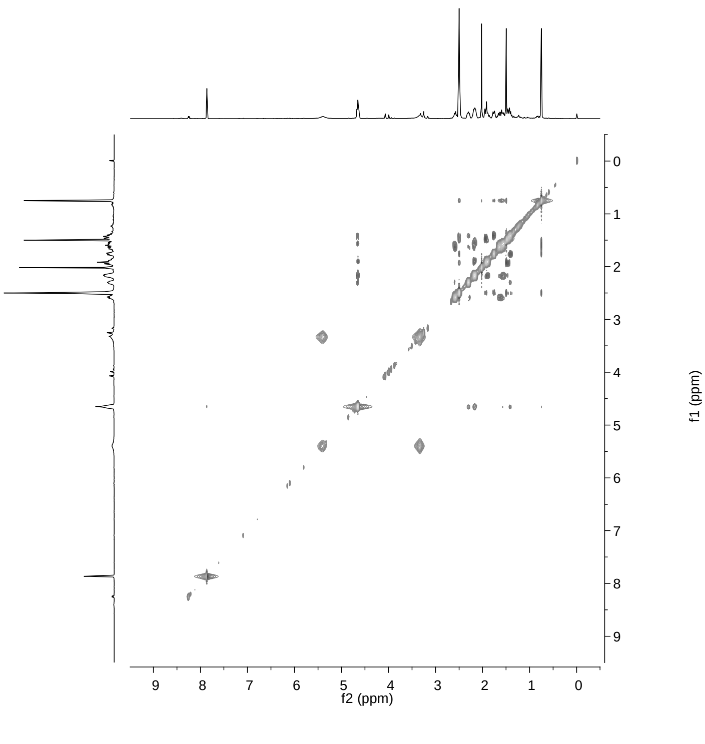

Supplementary Figure 47. The HMBC and NOESY spectra of 9.

(**a**) HMBC spectrum in DMSO-d_6 at 400 MHz; (**b**) NOESY spectrum in DMSO-d_6 at 400 MHz.

Single Mass Analysis
Tolerance = 5.0 mDa / DBE: min = -1.5, max = 50.0
Element prediction: Off
Number of isotope peaks used for i-FIT = 3

Monoisotopic Mass, Even Electron Ions
129 formula(e) evaluated with 3 results within limits (up to 50 closest results for each mass)
Elements Used:
C: 0-70 H: 0-120 O: 0-50 Na: 0-1
71-4-2
2016120508 162 (1.313) 1: TOF MS ES+
1.85e+006

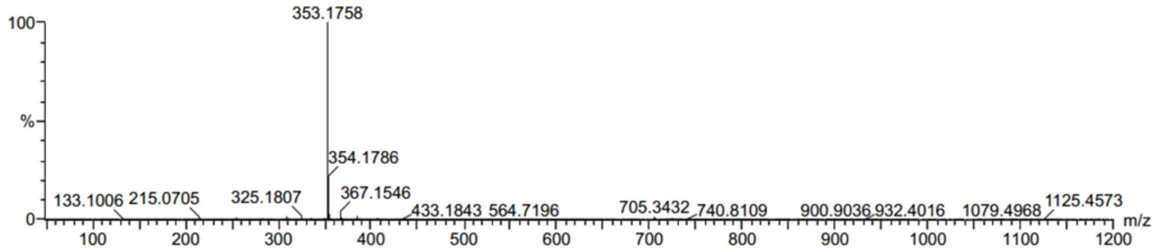

Minimum:				-1.5		
Maximum:		5.0	10.0	50.0		
Mass	Calc. Mass	mDa	PPM	DBE	i-FIT	Formula
353.1758	353.1753	0.5	1.4	10.5	6196.2	C22 H25 O4

Supplementary Figure 48. The HRESIMS spectrum of 10.

The HRESIMS spectrum (positive) showed *m/z* 353.1758 $[M + H]^+$ (calcd. for $C_{22}H_{25}O_4$, 353.1753).

a

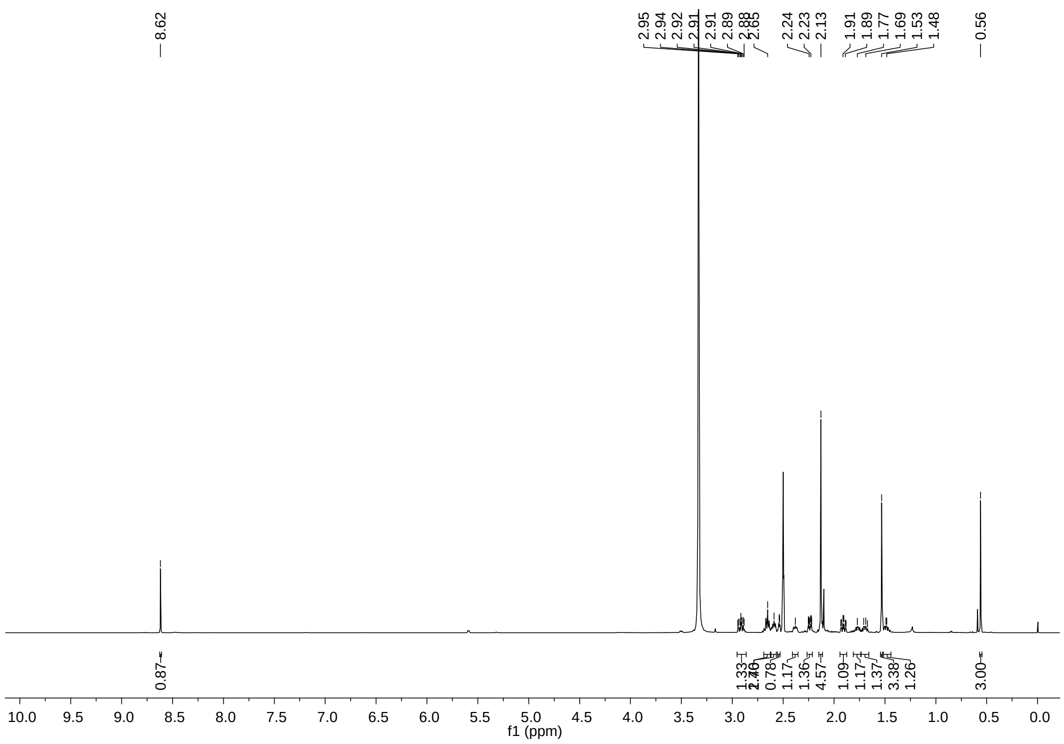

b

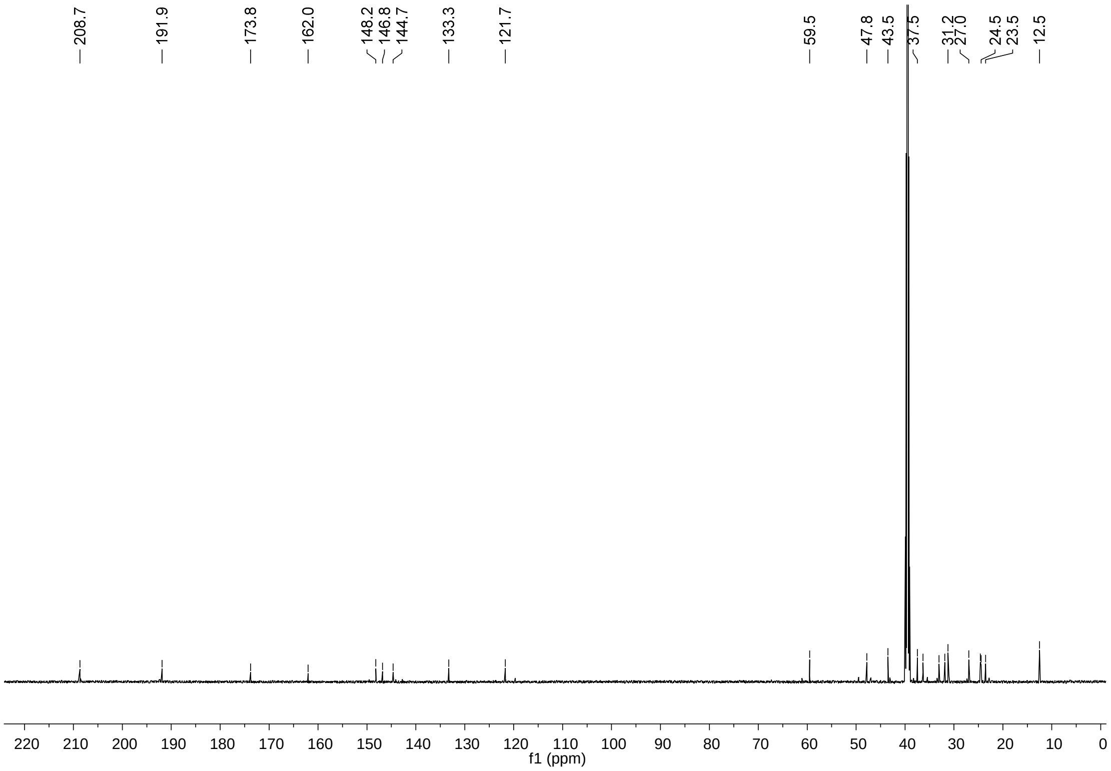

Supplementary Figure 49. The ^{1}H NMR and ^{13}C NMR spectra of 10.

(**a**) ^{1}H NMR spectrum in DMSO-d_6 at 600 MHz; (**b**) ^{13}C NMR spectrum in DMSO-d_6 at 150 MHz.

a

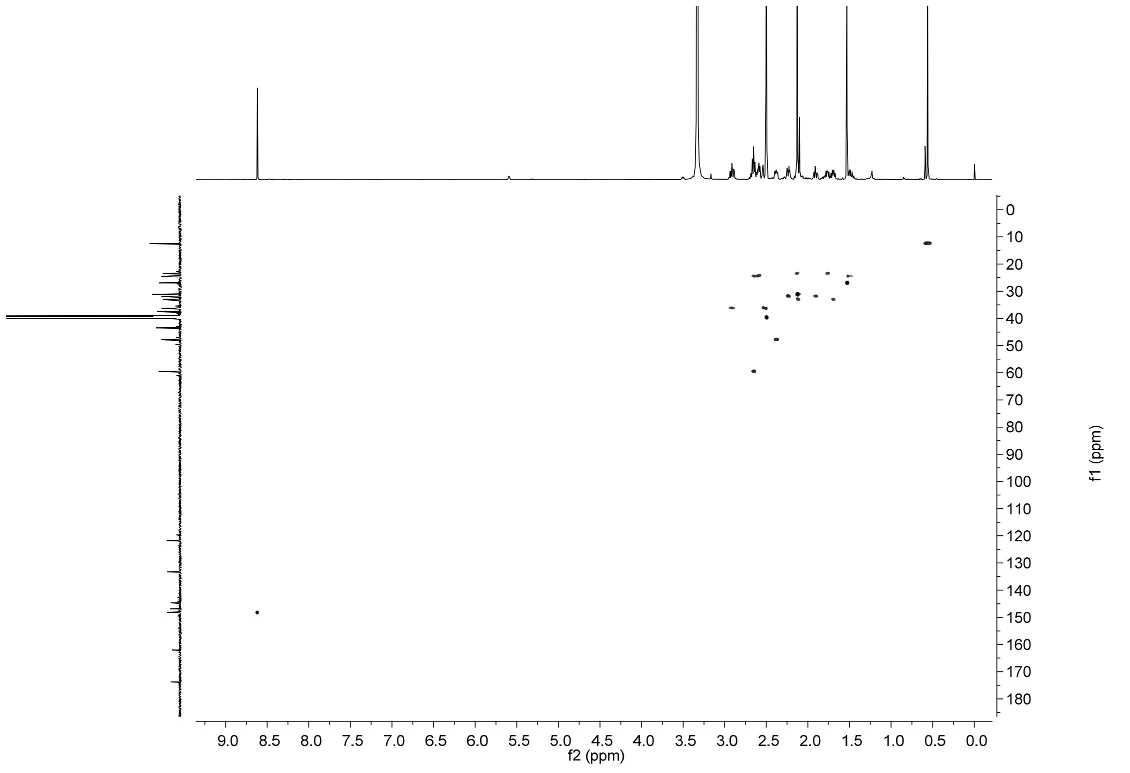

b

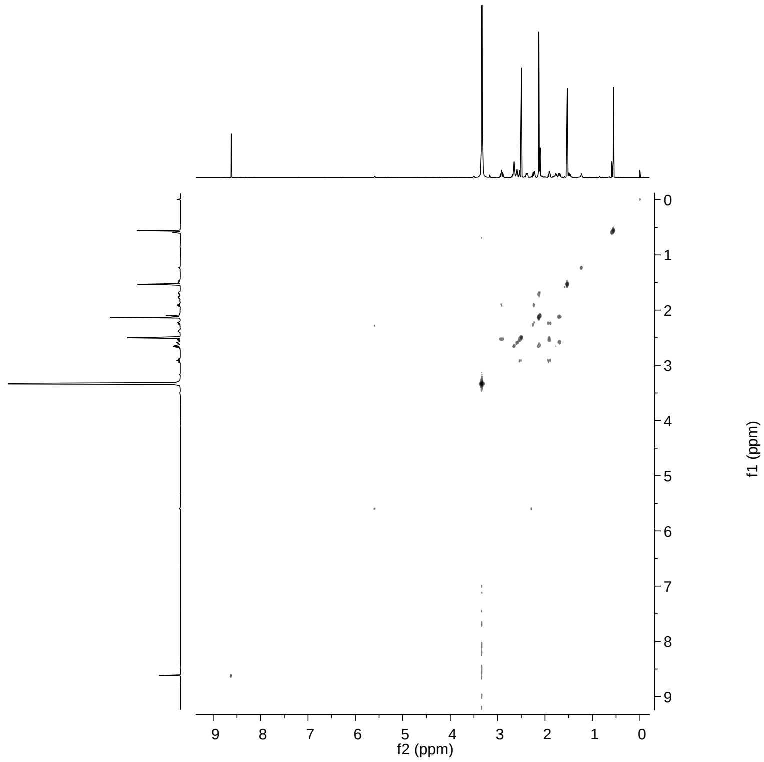

Supplementary Figure 50. The HSQC and ^{1}H-^{1}H COSY spectra of 10.

(**a**) HSQC spectrum in DMSO-d_6 at 600 MHz; (**b**) ^{1}H-^{1}H COSY spectrum in DMSO-d_6 at 600 MHz.

a

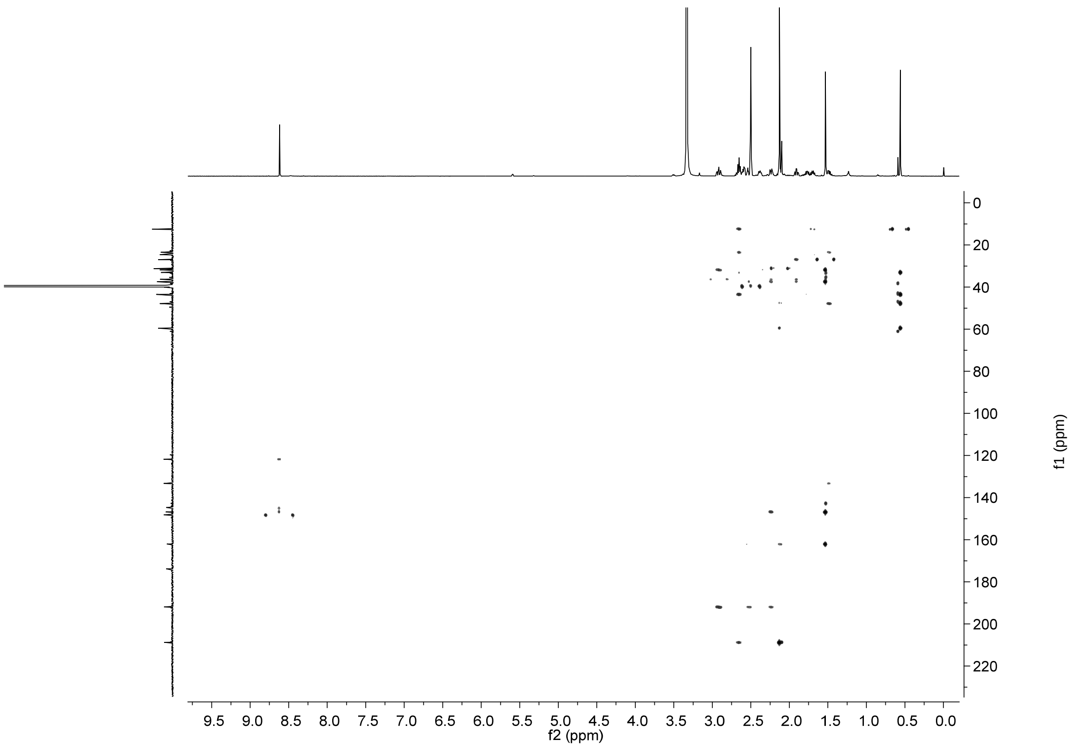

b

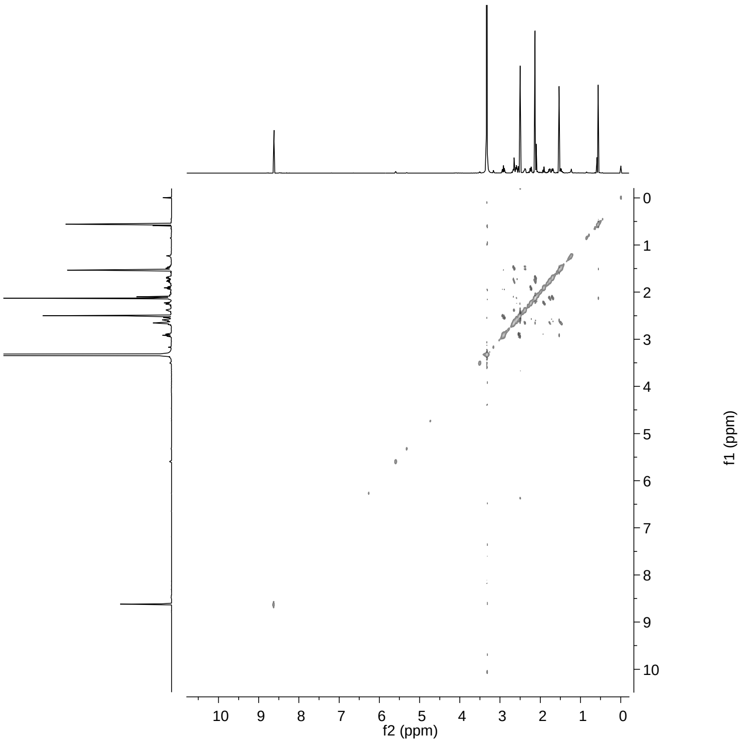

Supplementary Figure 51. The HMBC and ROESY spectra of 10.

(**a**) HMBC spectrum in DMSO-d_6 at 600 MHz; (**b**) ROESY spectrum in DMSO-d_6 at 600 MHz.

Single Mass Analysis
Tolerance = 5.0 mDa / DBE: min = -1.5, max = 50.0
Element prediction: Off
Number of isotope peaks used for i-FIT = 3

Monoisotopic Mass, Even Electron Ions
137 formula(e) evaluated with 2 results within limits (up to 50 closest results for each mass)
Elements Used:
C: 0-70 H: 0-120 O: 0-50 Na: 0-1
71-4-1
2016120507 141 (1.144)
1: TOF MS ES+
2.25e+006

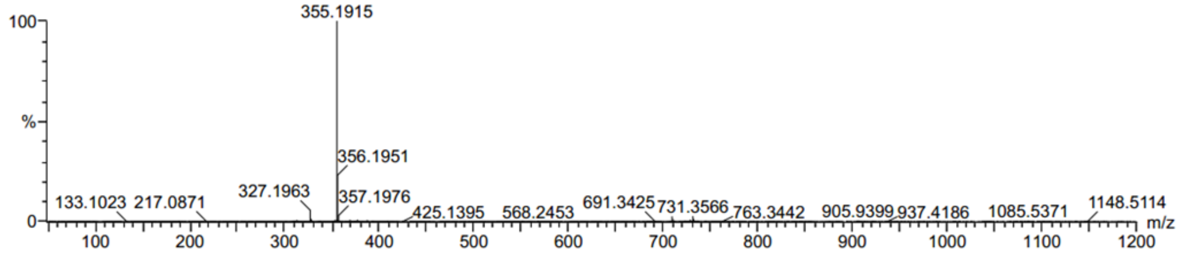

Minimum:				-1.5		
Maximum:		5.0	10.0	50.0		
Mass	Calc. Mass	mDa	PPM	DBE	i-FIT	Formula
355.1915	355.1909	0.6	1.7	9.5	3490.8	C22 H27 O4

Supplementary Figure 52. The HRESIMS spectrum of 11.

The HRESIMS spectrum (positive) showed *m/z* 355.1915 $[M + H]^+$ (calcd. for $C_{22}H_{27}O_4$, 355.1909).

a

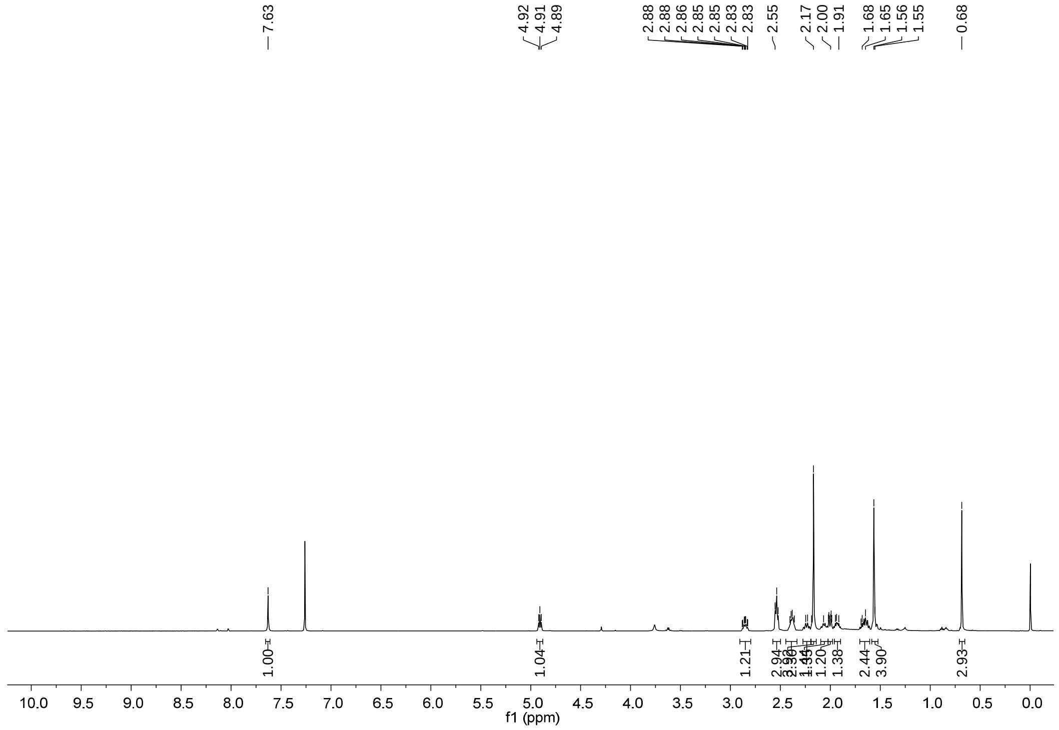

b

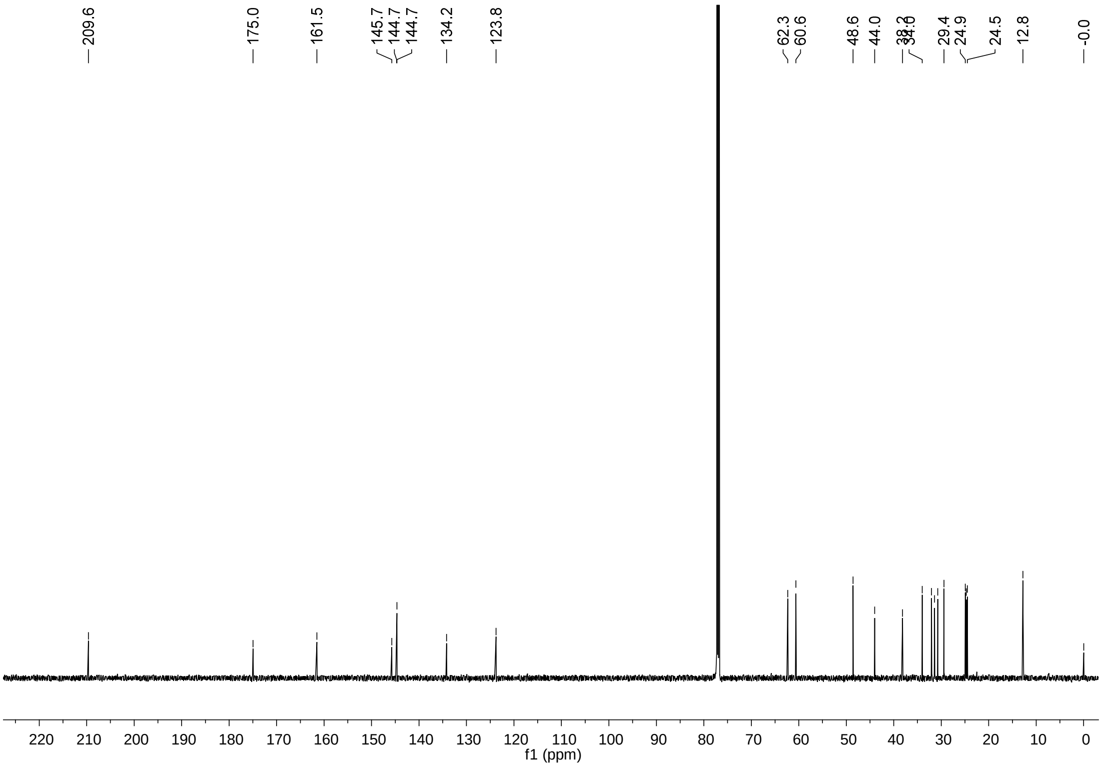

Supplementary Figure 53. The ^{1}H NMR and ^{13}C NMR spectra of 11.

(**a**) ^{1}H NMR spectrum in $CDCl_3$ at 600 MHz; (**b**) ^{13}C NMR spectrum in $CDCl_3$ at 150 MHz.

Single Mass Analysis
Tolerance = 5.0 mDa / DBE: min = -1.5, max = 50.0
Element prediction: Off
Number of isotope peaks used for i-FIT = 3

Monoisotopic Mass, Even Electron Ions
74 formula(e) evaluated with 1 results within limits (up to 50 best isotopic matches for each mass)
Elements Used:
C: 0-80 H: 0-100 O: 0-20

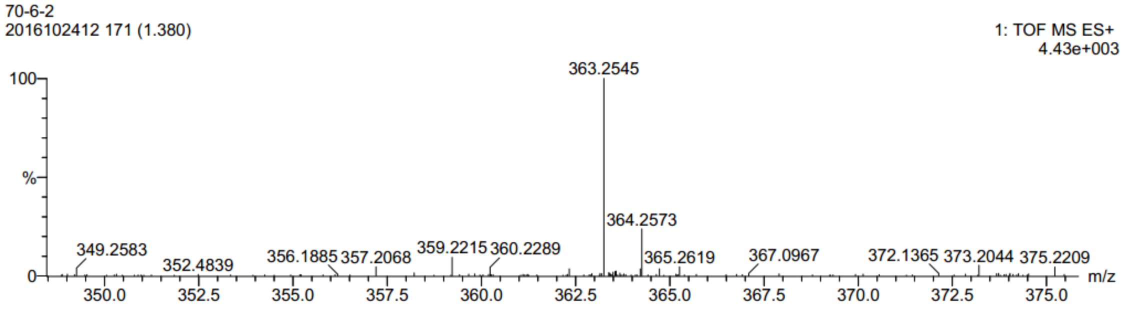

Minimum:				-1.5				
Maximum:		5.0	10.0	50.0				

Mass	Calc. Mass	mDa	PPM	DBE	i-FIT	Norm	Conf(%)	Formula
363.2545	363.2535	1.0	2.8	5.5	190.4	n/a	n/a	C22 H35 O4

Supplementary Figure 54. The HRESIMS spectrum of 12.

The HRESIMS spectrum (positive) showed *m/z* 363.2545 [M + H]$^+$ (calcd. for $C_{22}H_{35}O_4$, 363.2535).

a

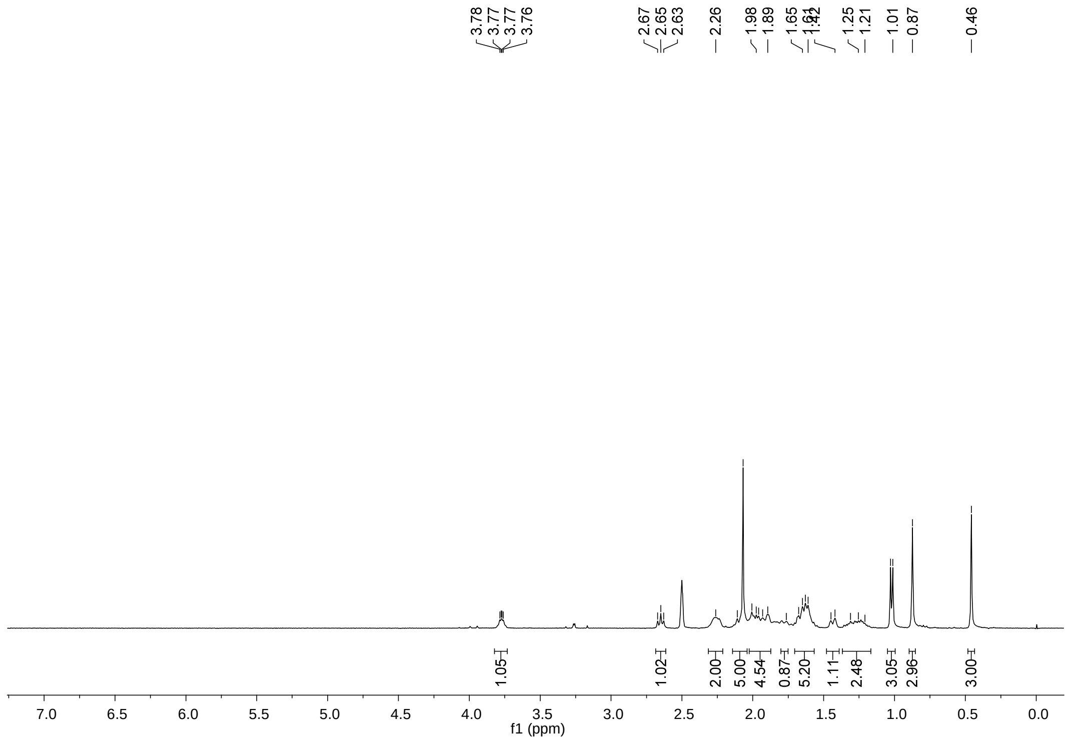

b

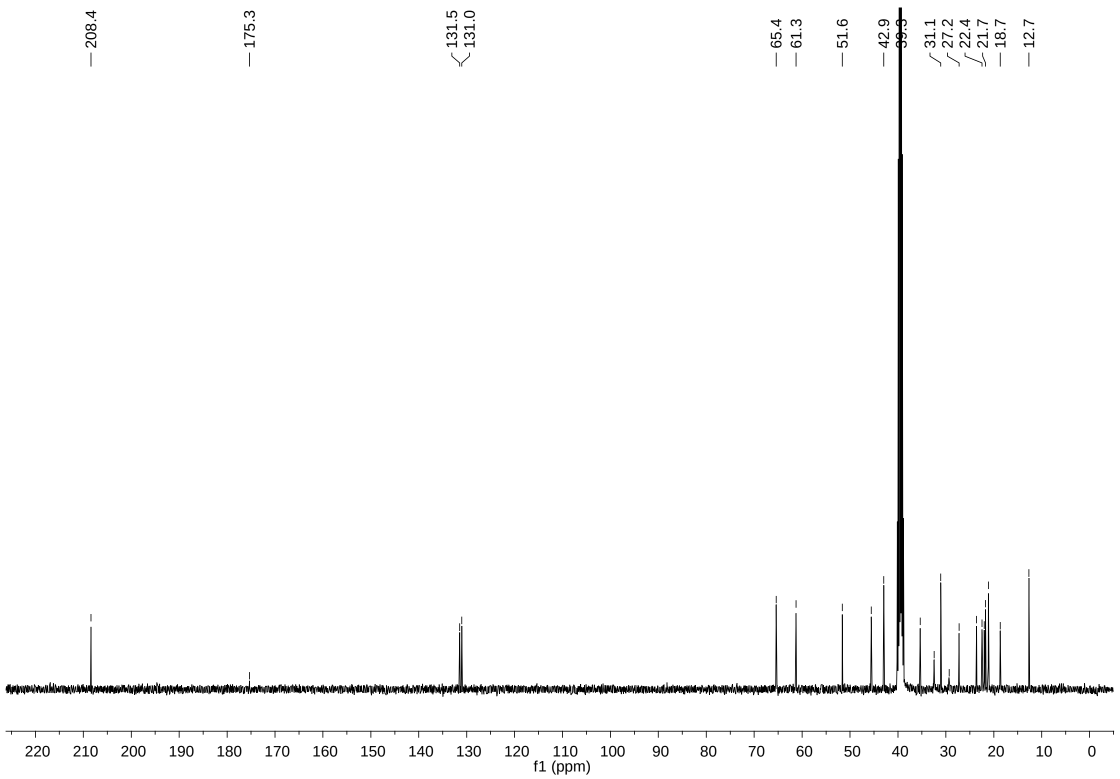

Supplementary Figure 55. The ^{1}H NMR and ^{13}C NMR spectra of 12.

(**a**) ^{1}H NMR spectrum in DMSO-d_6 at 400 MHz; (**b**) ^{13}C NMR spectrum in DMSO-d_6 at 100 MHz.

Tolerance = 5.0 PPM / DBE: min = -1.5, max = 50.0
Element prediction: Off
Number of isotope peaks used for i-FIT = 3

Monoisotopic Mass, Even Electron Ions
1902 formula(e) evaluated with 8 results within limits (up to 50 best isotopic matches for each mass)
Elements Used:
C: 0-500 H: 0-1000 N: 0-200 O: 0-200 Na: 0-1

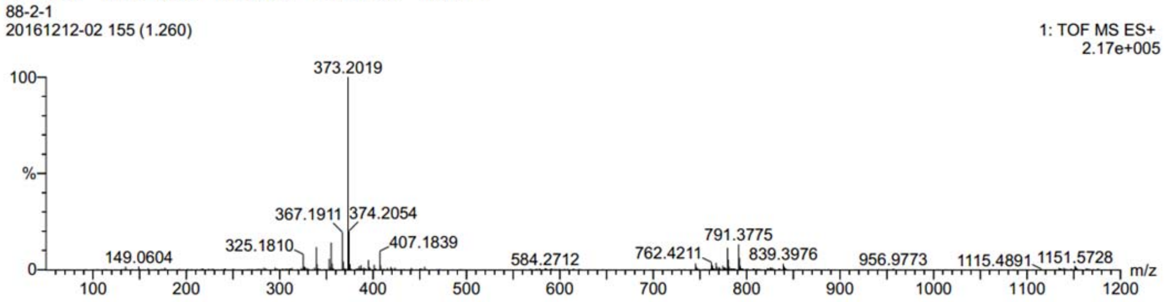

Minimum:	80.00				-1.5				
Maximum:	100.00		5.0	5.0	50.0				
Mass	RA	Calc. Mass	mDa	PPM	DBE	i-FIT	Norm	Conf(%)	Formula
373.2019	100.00	373.2015	0.4	1.1	8.5	242.0	0.228	79.64	C22 H29 O5

Supplementary Figure 56. The HRESIMS spectrum of 13.

The HRESIMS spectrum (positive) showed *m/z* 373.2019 $[M + H]^+$ (calcd. for $C_{22}H_{29}O_5$, 373.2015).

a

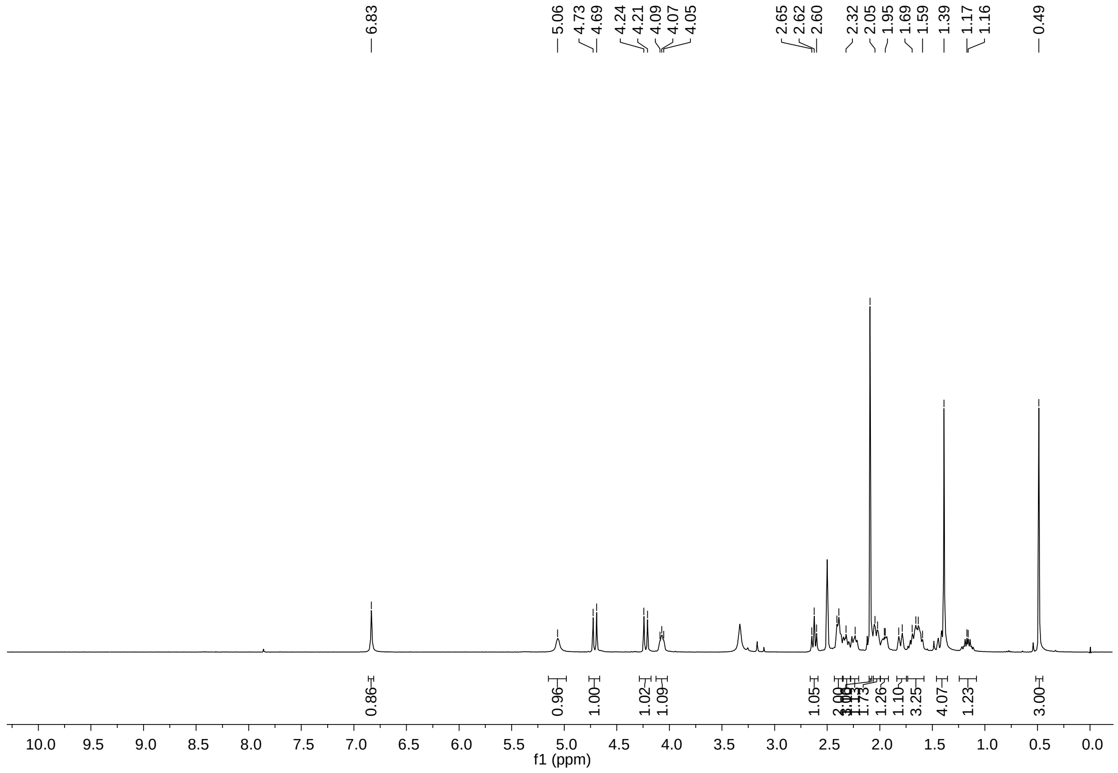

b

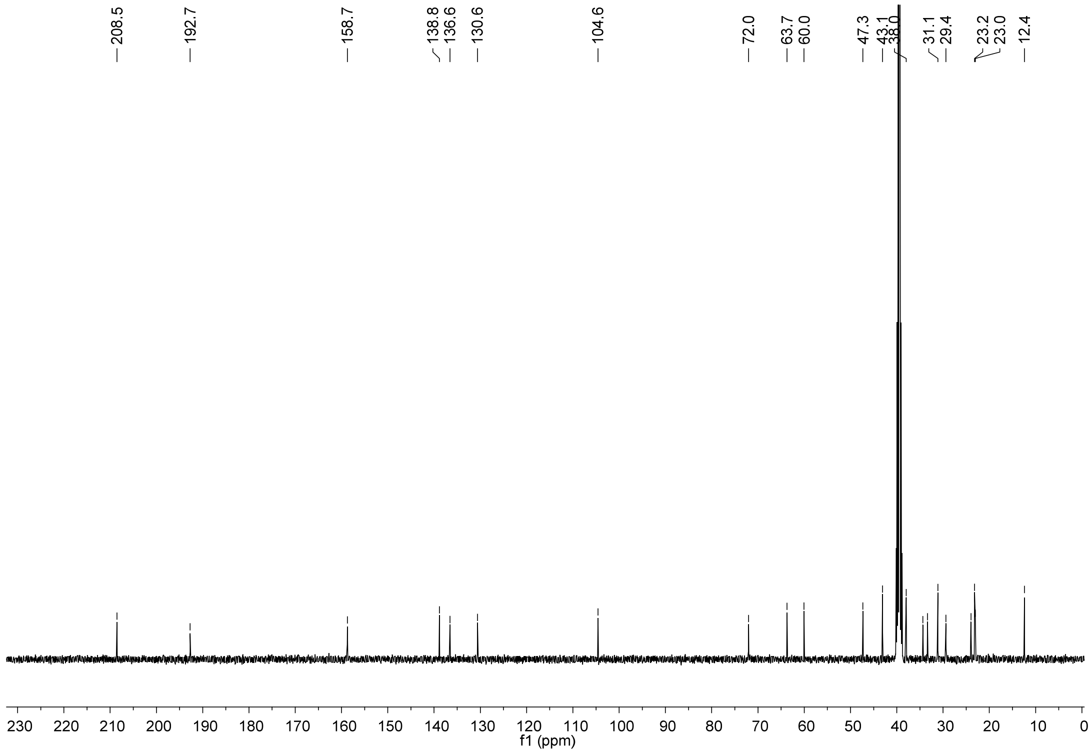

Supplementary Figure 57. The ^{1}H NMR and ^{13}C NMR spectra of 13.

(**a**) ^{1}H NMR spectrum in DMSO-d_6 at 400 MHz; (**b**) ^{13}C NMR spectrum in DMSO-d_6 at 100 MHz.

a

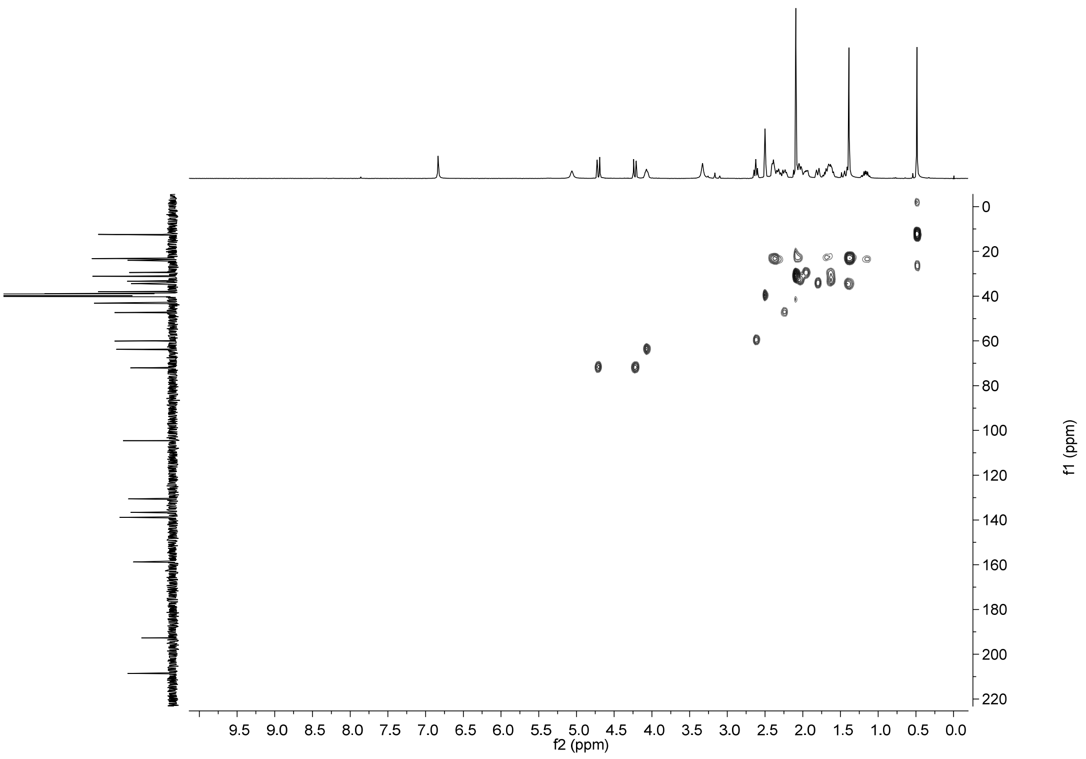

b

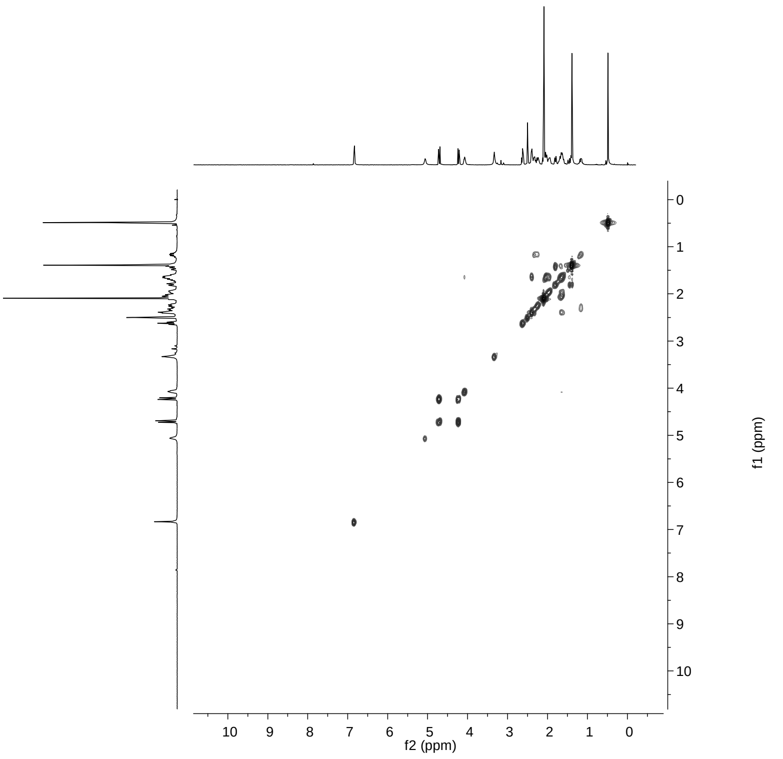

Supplementary Figure 58. The HSQC and ^{1}H-^{1}H COSY spectra of 13.

(**a**) HSQC spectrum in DMSO-d_6 at 400 MHz; (**b**) ^{1}H-^{1}H COSY spectrum in DMSO-d_6 at 400 MHz.

a

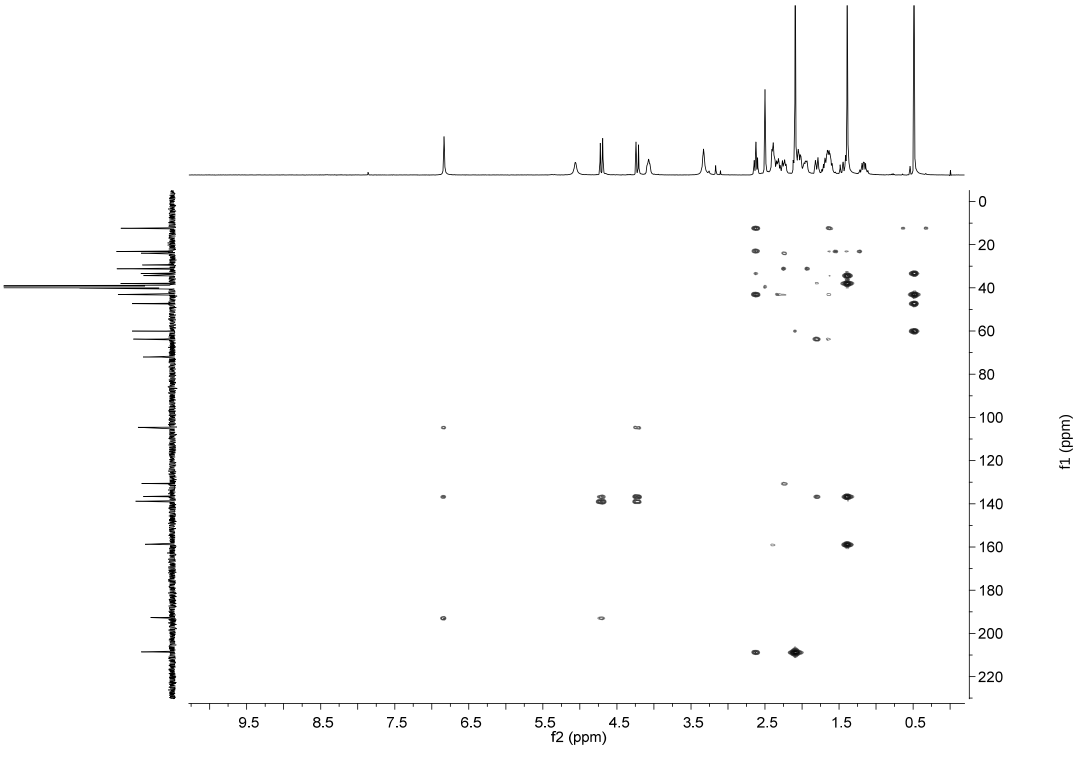

b

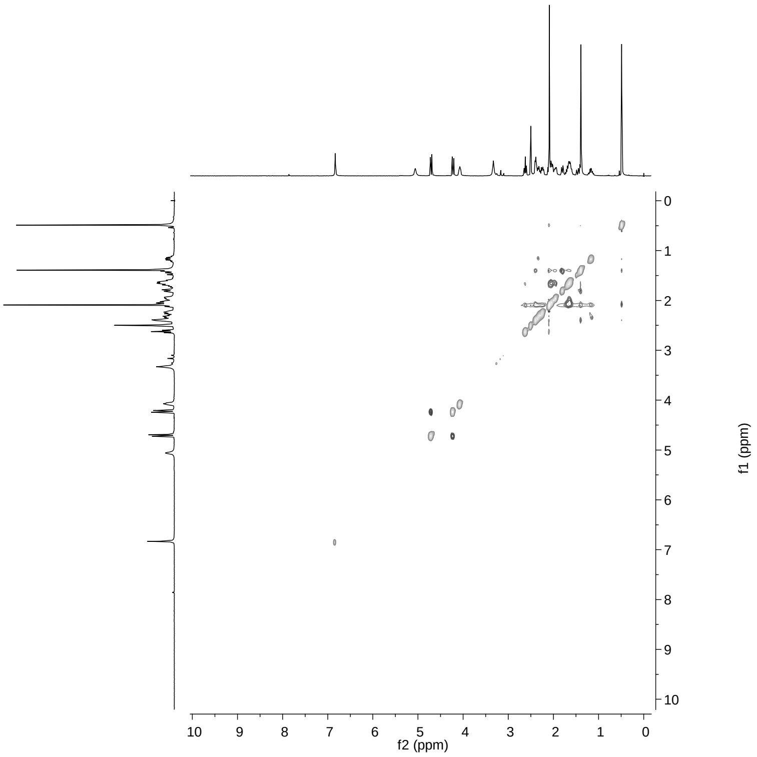

Supplementary Figure 59. The HMBC and ROESY spectra of 13.

(**a**) HMBC spectrum in DMSO-d_6 at 400 MHz; (**b**) ROESY spectrum in DMSO-d_6 at 400 MHz.

Single Mass Analysis
Tolerance = 5.0 mDa / DBE: min = -1.5, max = 50.0
Element prediction: Off
Number of isotope peaks used for i-FIT = 3

Monoisotopic Mass, Even Electron Ions
158 formula(e) evaluated with 3 results within limits (up to 50 best isotopic matches for each mass)
Elements Used:
C: 0-500 H: 0-1000 O: 0-200 Na: 0-1
3286-2-2
2016112116 72 (0.595)

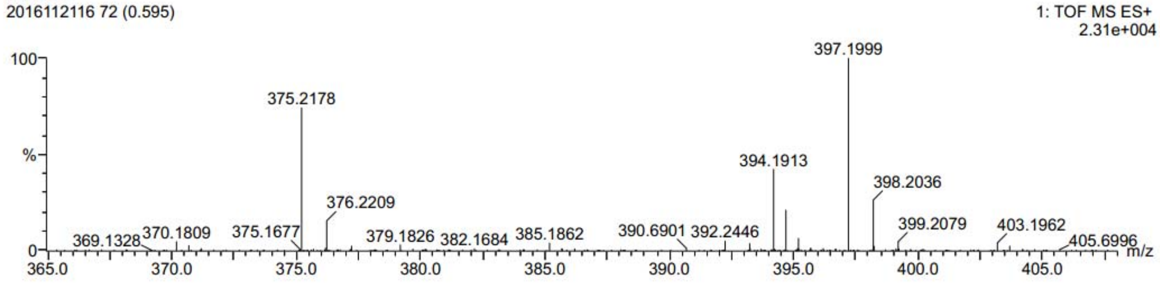

Minimum:				-1.5				
Maximum:		5.0	10.0	50.0				

Mass	Calc. Mass	mDa	PPM	DBE	i-FIT	Norm	Conf(%)	Formula
397.1999	397.1991	0.8	2.0	7.5	127.7	4.869	0.77	C22 H30 O5 Na

Supplementary Figure 60. The HRESIMS spectrum of 14.

The HRESIMS spectrum (positive) showed *m/z* 397.1999 [M + Na]$^+$ (calcd. for $C_{22}H_{30}O_5$ Na, 397.1991).

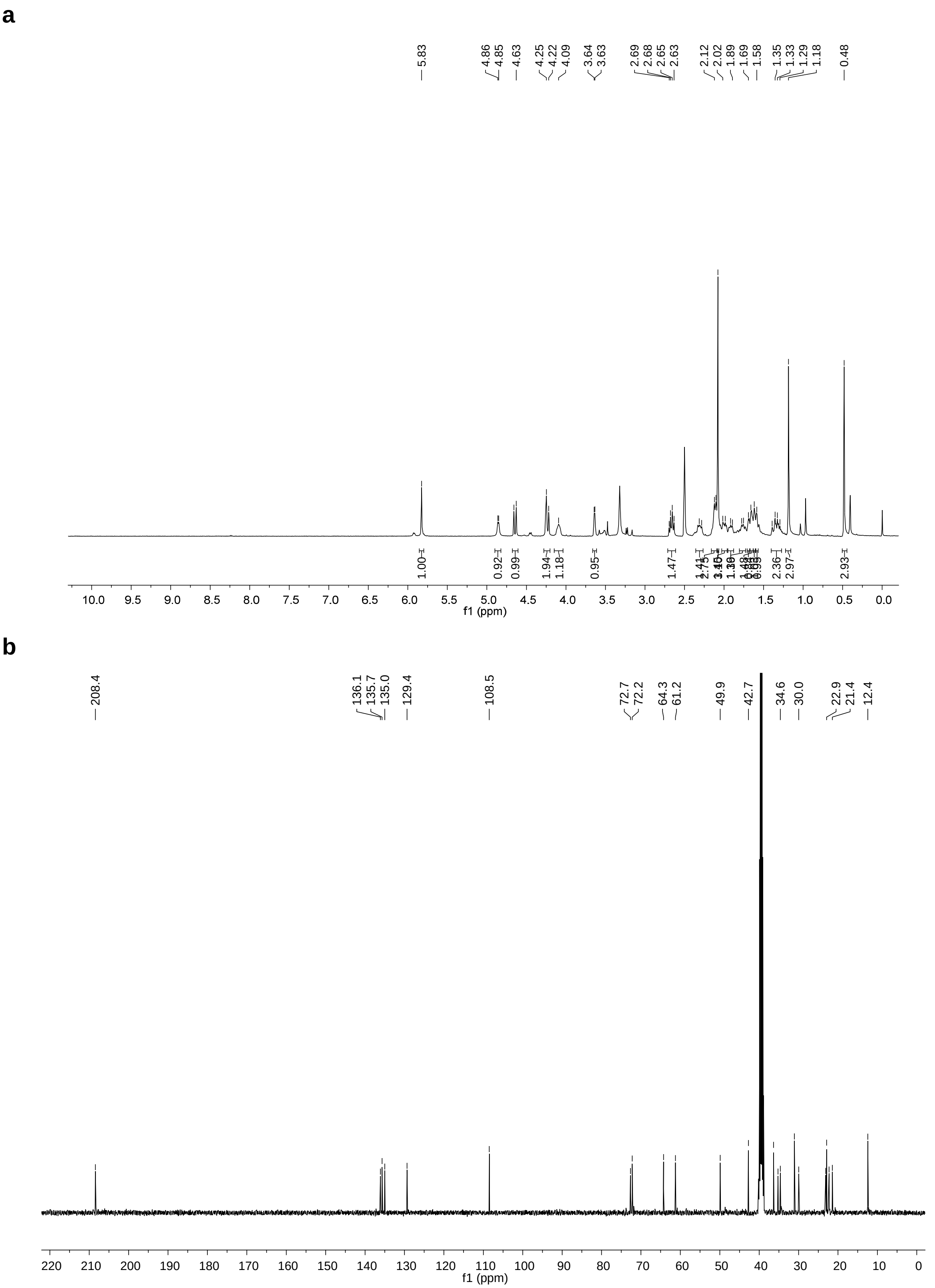

Supplementary Figure 61. The ^{1}H NMR and ^{13}C NMR spectra of 14.

(**a**) ^{1}H NMR spectrum in DMSO-d_6 at 400 MHz; (**b**) ^{13}C NMR spectrum in DMSO-d_6 at 100 MHz.

a

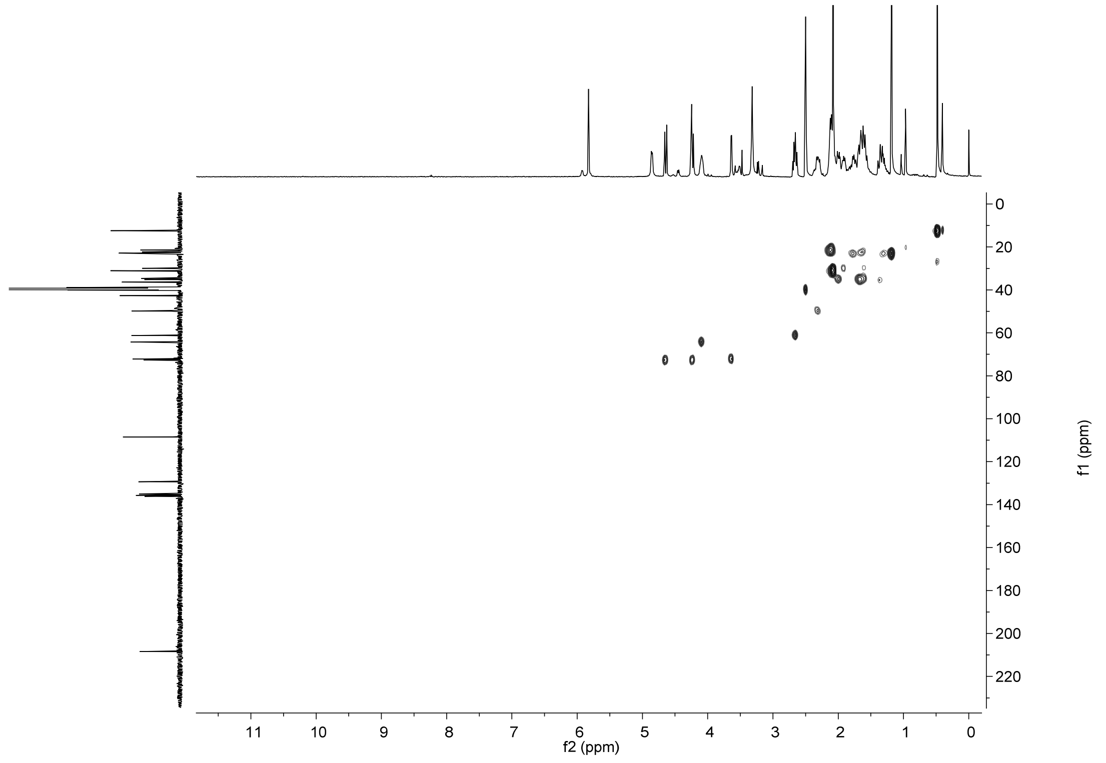

b

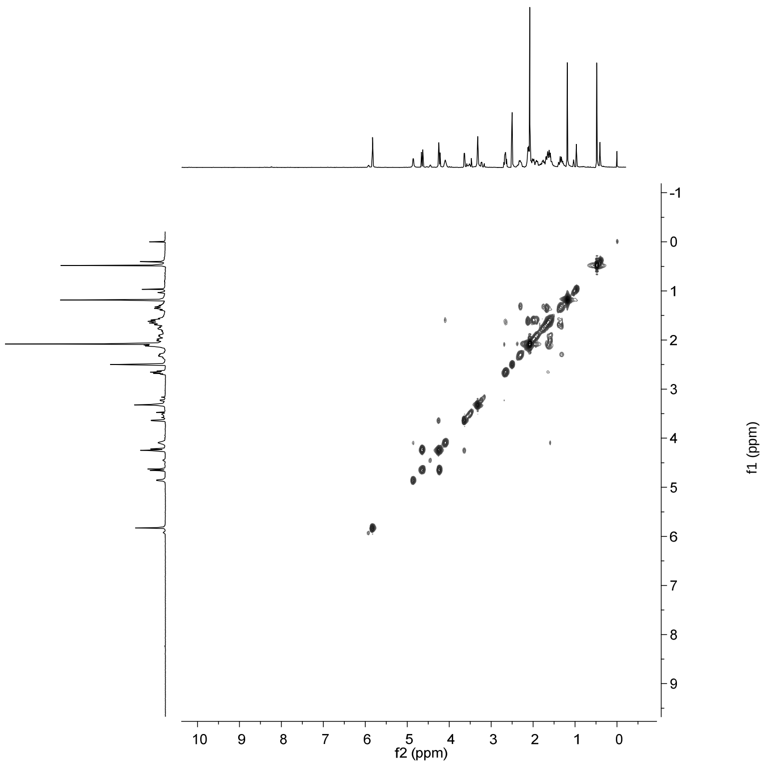

Supplementary Figure 62. The HSQC and ^{1}H-^{1}H COSY spectra of 14.

(**a**) HSQC spectrum in DMSO-d_6 at 400 MHz; (**b**) ^{1}H-^{1}H COSY spectrum in DMSO-d_6 at 400 MHz.

a

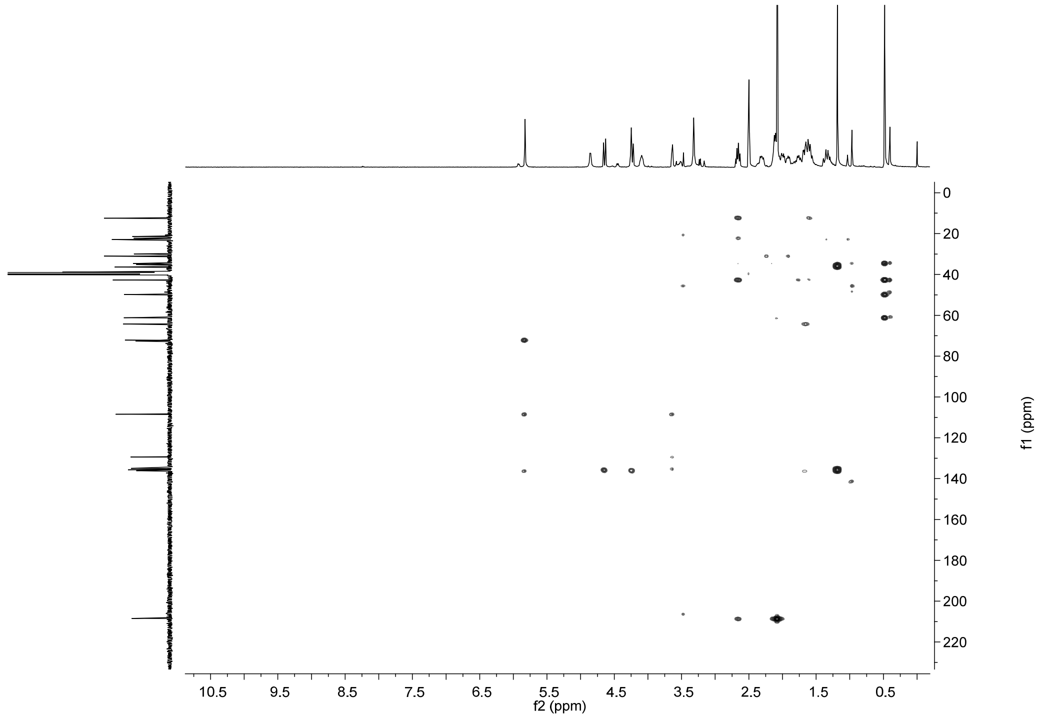

b

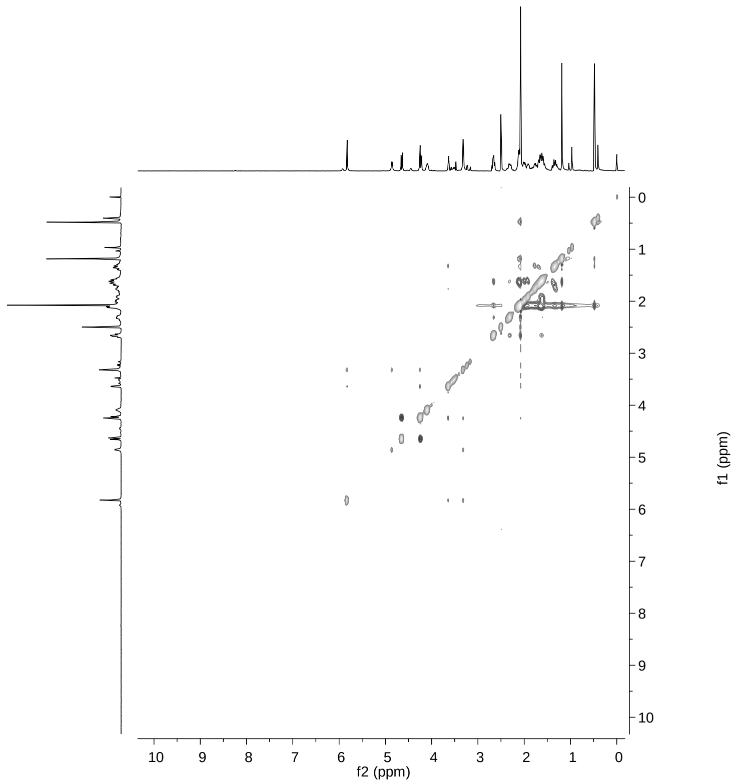

Supplementary Figure 63. The HMBC and ROESY spectra of 14.

(**a**) HMBC spectrum in DMSO-d_6 at 400 MHz; (**b**) ROESY spectrum in DMSO-d_6 at 400 MHz.

Single Mass Analysis
Tolerance = 5.0 PPM / DBE: min = -1.5, max = 50.0
Element prediction: Off
Number of isotope peaks used for i-FIT = 3

Monoisotopic Mass, Even Electron Ions
2217 formula(e) evaluated with 11 results within limits (up to 50 best isotopic matches for each mass)
Elements Used:
C: 0-500 H: 0-1000 N: 0-200 O: 0-200 Na: 0-1

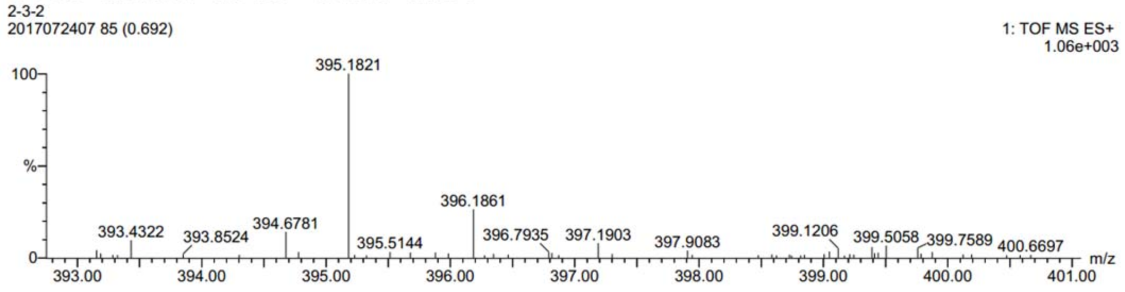

Mass	Calc. Mass	mDa	PPM	DBE	i-FIT	Norm	Conf(%)	Formula
Minimum:				-1.5				
Maximum:		5.0	5.0	50.0				
395.1821	395.1834	-1.3	-3.3	8.5	91.2	0.694	49.94	C22 H28 O5 Na

Supplementary Figure 64. The HRESIMS spectrum of 15.

The HRESIMS spectrum (positive) showed *m/z* 395.1821 [M + Na]$^+$ (calcd. for $C_{22}H_{30}O_5$ Na, 395.1834).

a

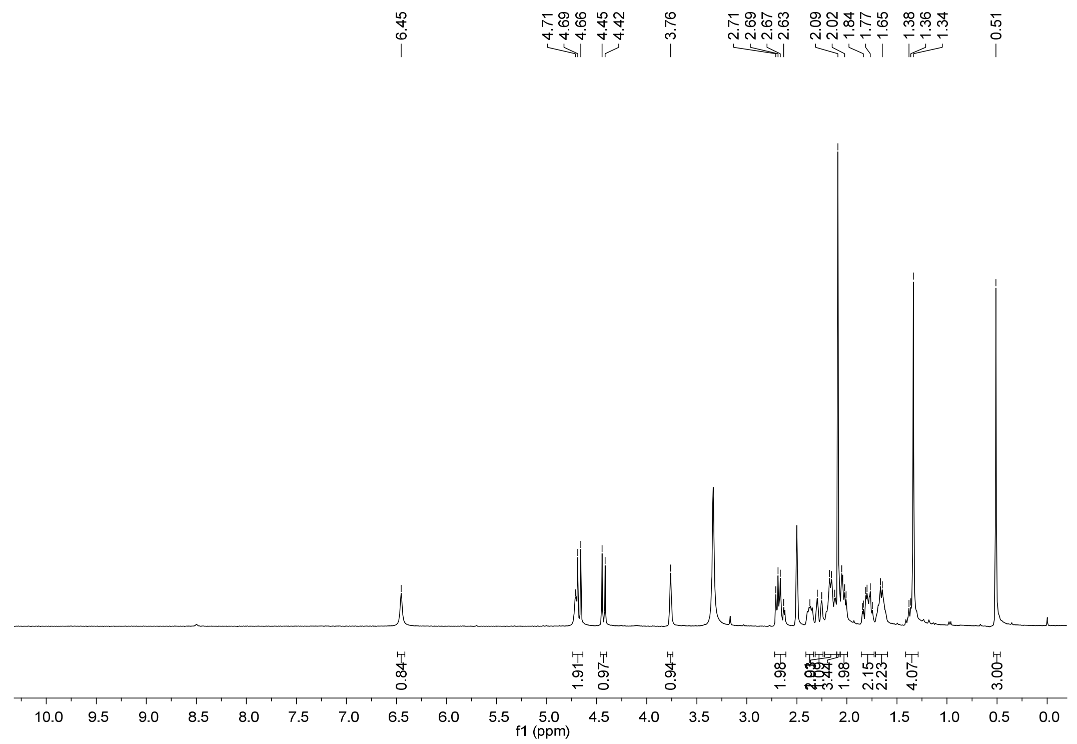

b

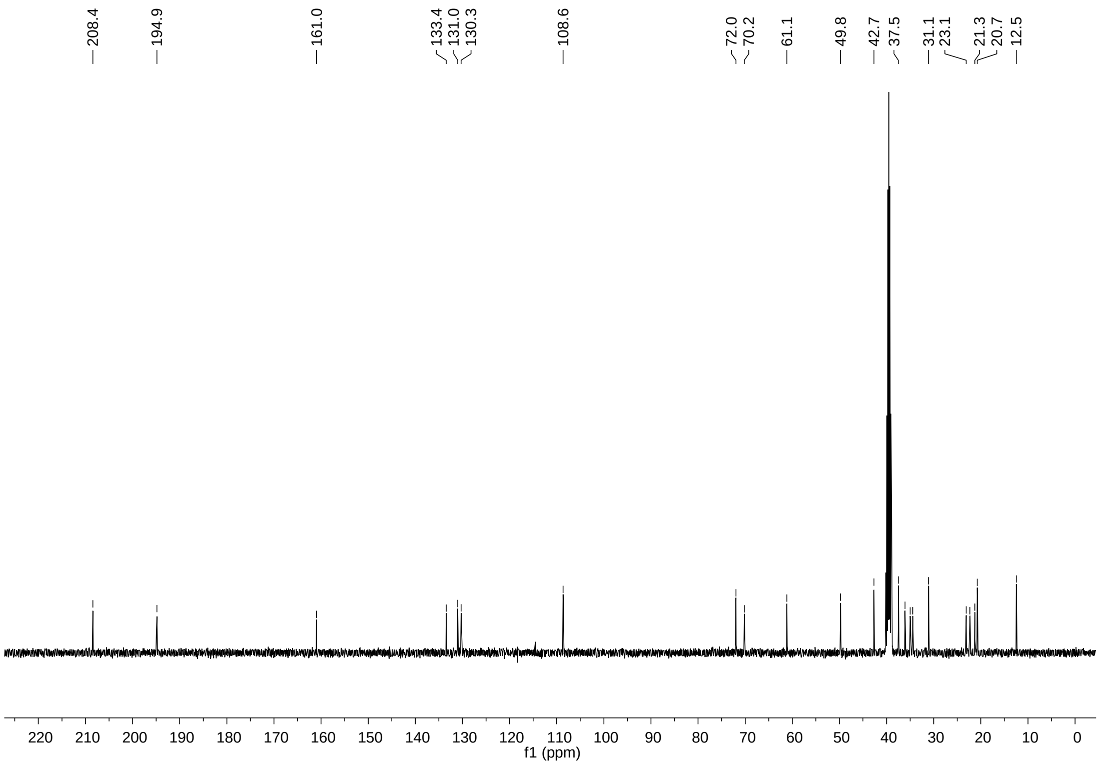

Supplementary Figure 65. The ^{1}H NMR and ^{13}C NMR spectra of 15.

(**a**) ^{1}H NMR spectrum in DMSO-d_6 at 400 MHz; (**b**) ^{13}C NMR spectrum in DMSO-d_6 at 100 MHz.

a

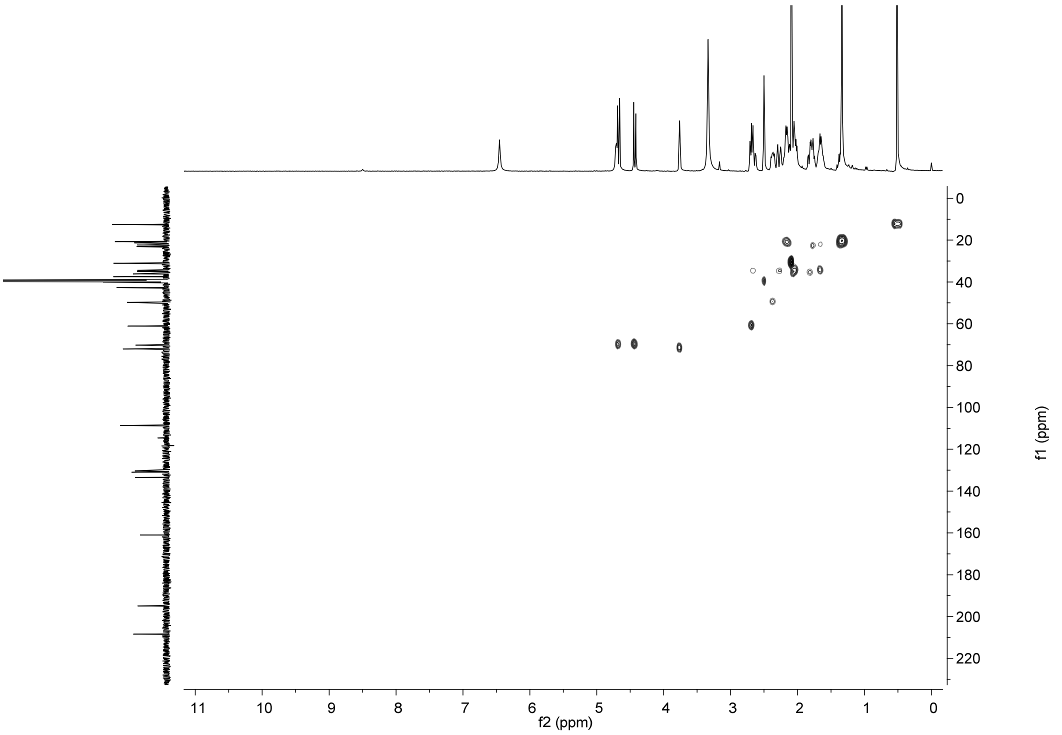

b

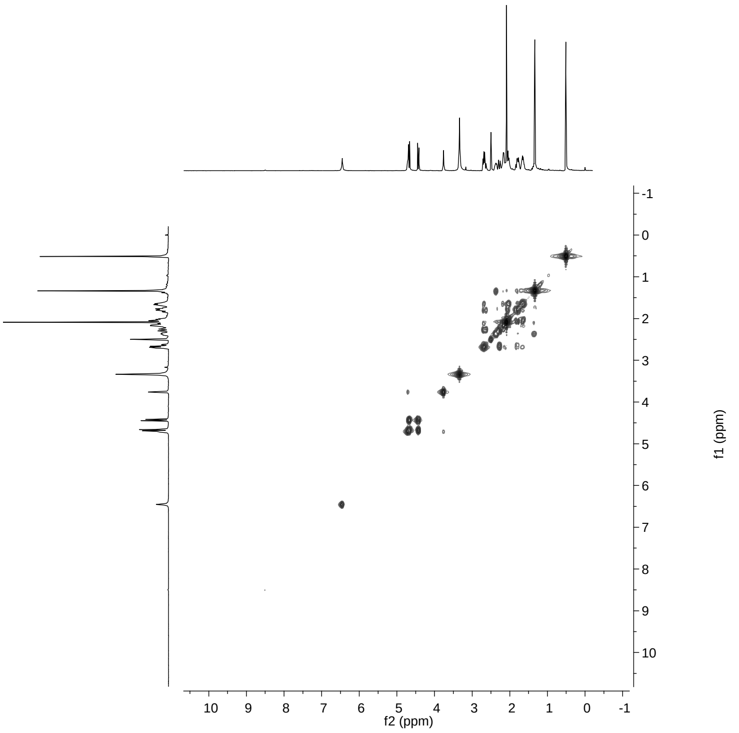

Supplementary Figure 66. The HSQC and ^{1}H-^{1}H COSY spectra of 15.

(**a**) HSQC spectrum in DMSO-d_6 at 400 MHz; (**b**) ^{1}H-^{1}H COSY spectrum in DMSO-d_6 at 400 MHz.

a

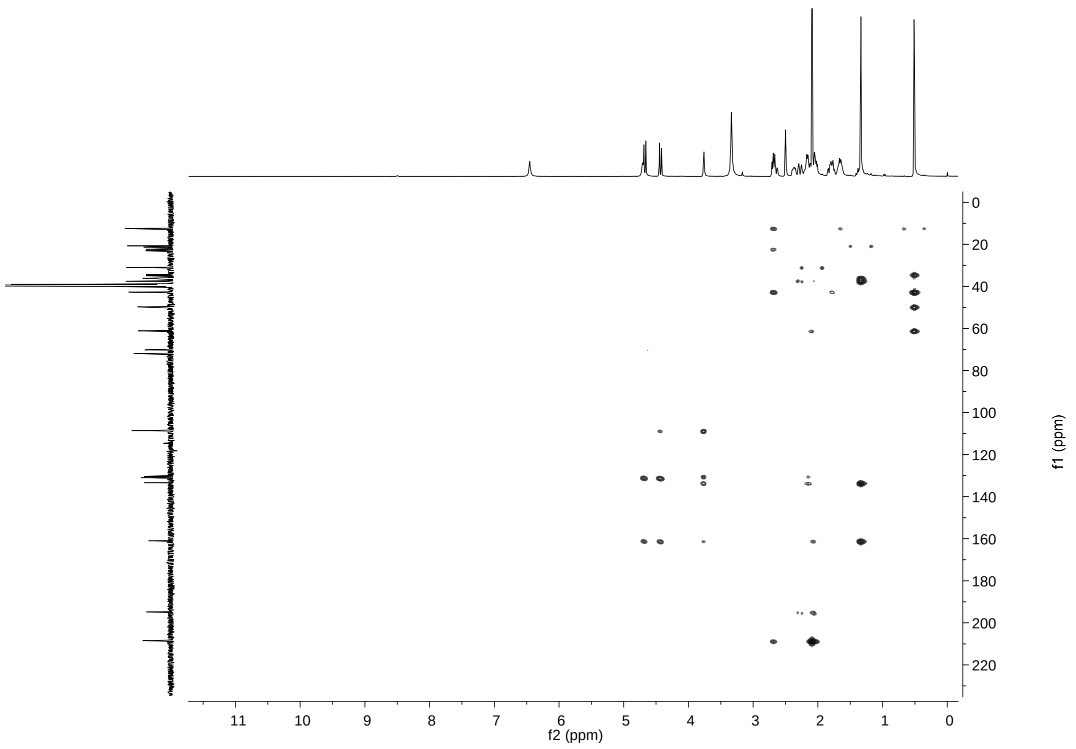

b

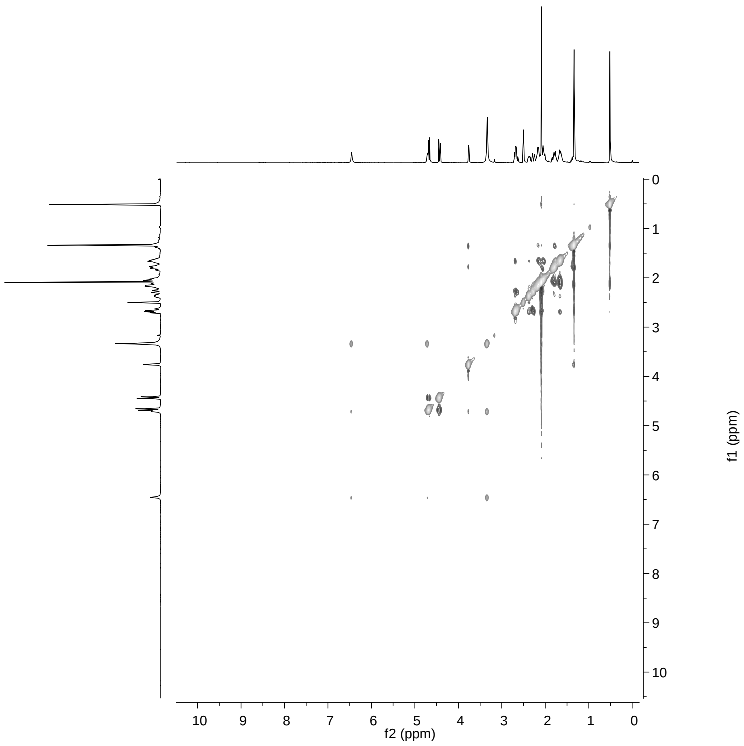

Supplementary Figure 67. The HMBC and ROESY spectra of 15.

(**a**) HMBC spectrum in DMSO-d_6 at 400 MHz; (**b**) ROESY spectrum in DMSO-d_6 at 400 MHz.

Single Mass Analysis
Tolerance = 10.0 PPM / DBE: min = -1.5, max = 50.0
Element prediction: Off
Number of isotope peaks used for i-FIT = 3

Monoisotopic Mass, Even Electron Ions
66 formula(e) evaluated with 1 results within limits (up to 50 closest results for each mass)
Elements Used:
C: 0-100 H: 0-100 O: 0-50
YUN-3
2017110604 243 (1.963) 1: TOF MS ES+
2.06e+006

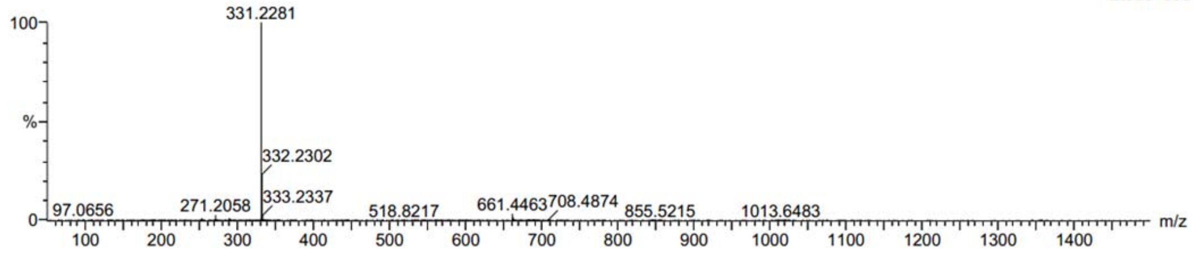

		mDa	PPM	DBE				
Minimum:				-1.5				
Maximum:		5.0	10.0	50.0				

Mass	Calc. Mass	mDa	PPM	DBE	i-FIT	Norm	Conf(%)	Formula
331.2281	331.2273	0.8	2.4	6.5	697.1	n/a	n/a	C21 H31 O3

Supplementary Figure 68. The HRESIMS spectrum of 21.

The HRESIMS spectrum (positive) showed *m/z* 331.2281 $[M + H]^+$ (calcd. for $C_{21}H_{31}O_3$, 331.2273).

a

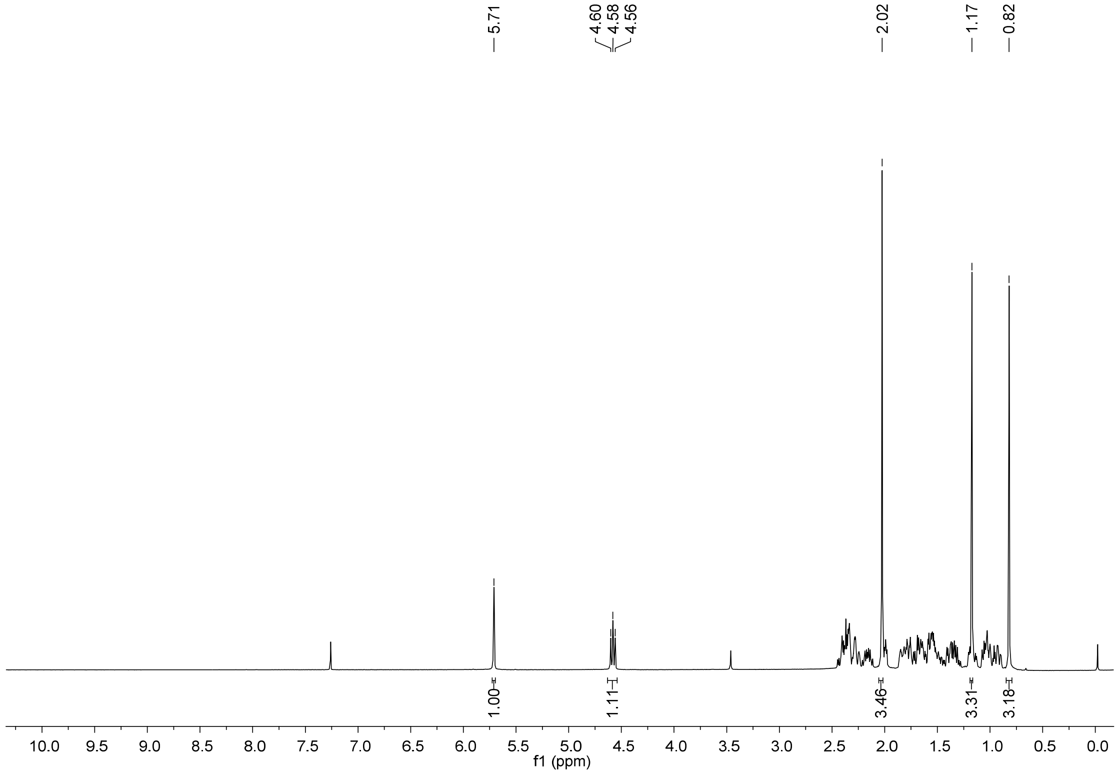

b

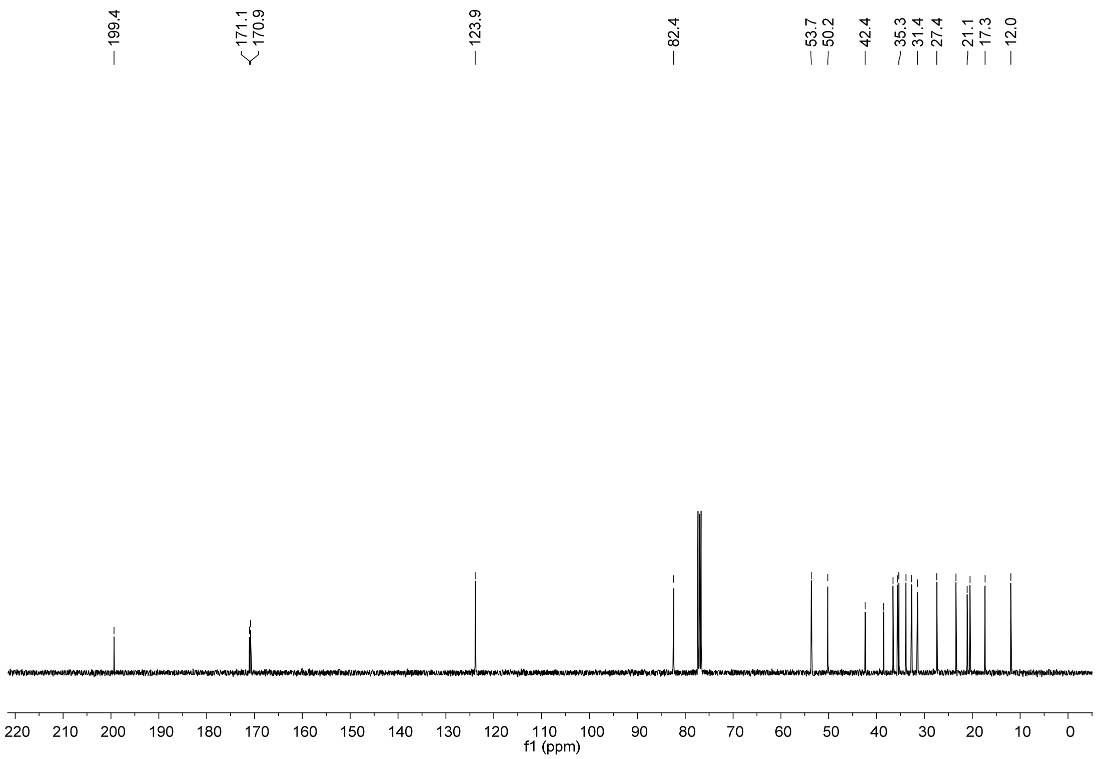

Supplementary Figure 69. The ^{1}H NMR and ^{13}C NMR spectra of 21.

(**a**) ^{1}H NMR spectrum in $CDCl_3$ at 400 MHz; (**b**) ^{13}C NMR spectrum in $CDCl_3$ at 100 MHz.

Single Mass Analysis
Tolerance = 10.0 PPM / DBE: min = -1.5, max = 50.0
Element prediction: Off
Number of isotope peaks used for i-FIT = 3

Monoisotopic Mass, Even Electron Ions
57 formula(e) evaluated with 1 results within limits (up to 50 closest results for each mass)
Elements Used:
C: 0-100 H: 0-100 O: 0-50
YUN-1
2017110602 155 (1.260)

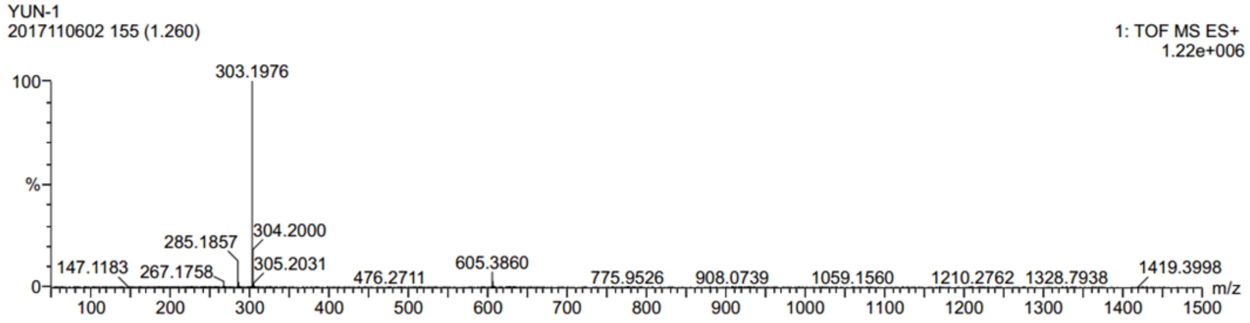

Minimum:				-1.5				
Maximum:		5.0	10.0	50.0				
Mass	Calc. Mass	mDa	PPM	DBE	i-FIT	Norm	Conf(%)	Formula
303.1976	303.1960	1.6	5.3	6.5	561.7	n/a	n/a	C19 H27 O3

Supplementary Figure 70. The HRESIMS spectrum of 24.

The HRESIMS spectrum (positive) showed *m/z* 303.1976 $[M + H]^+$ (calcd. for $C_{19}H_{27}O_3$, 303.1960).

a

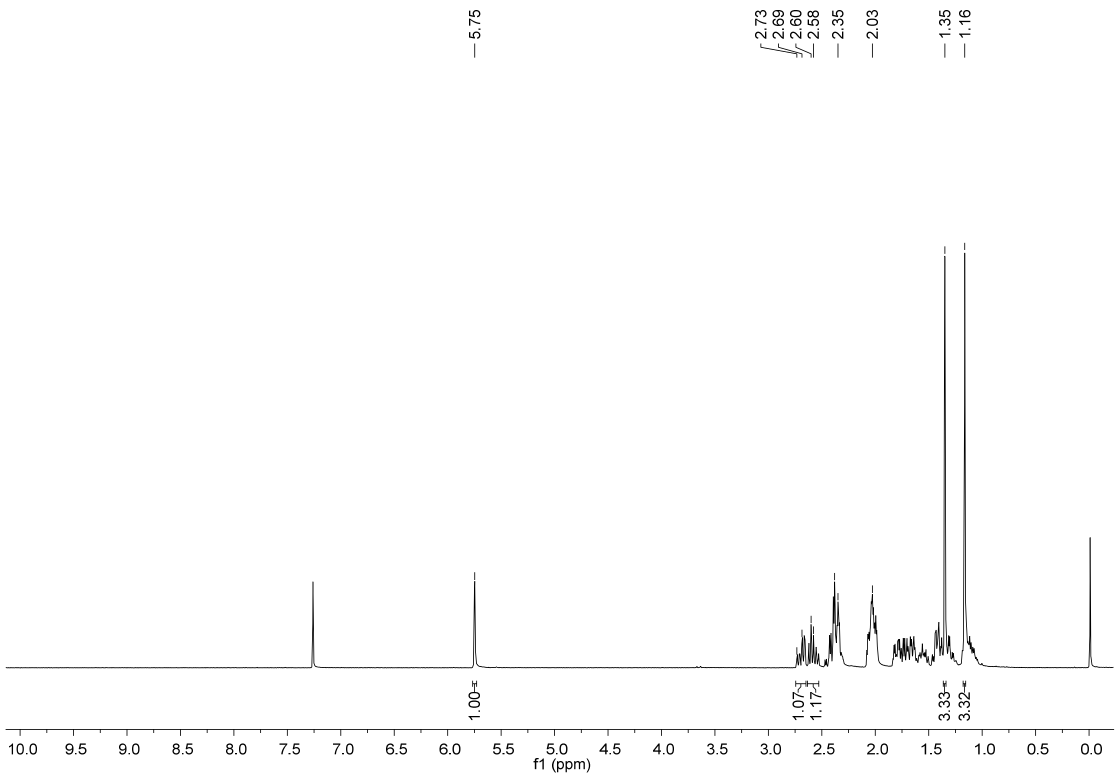

b

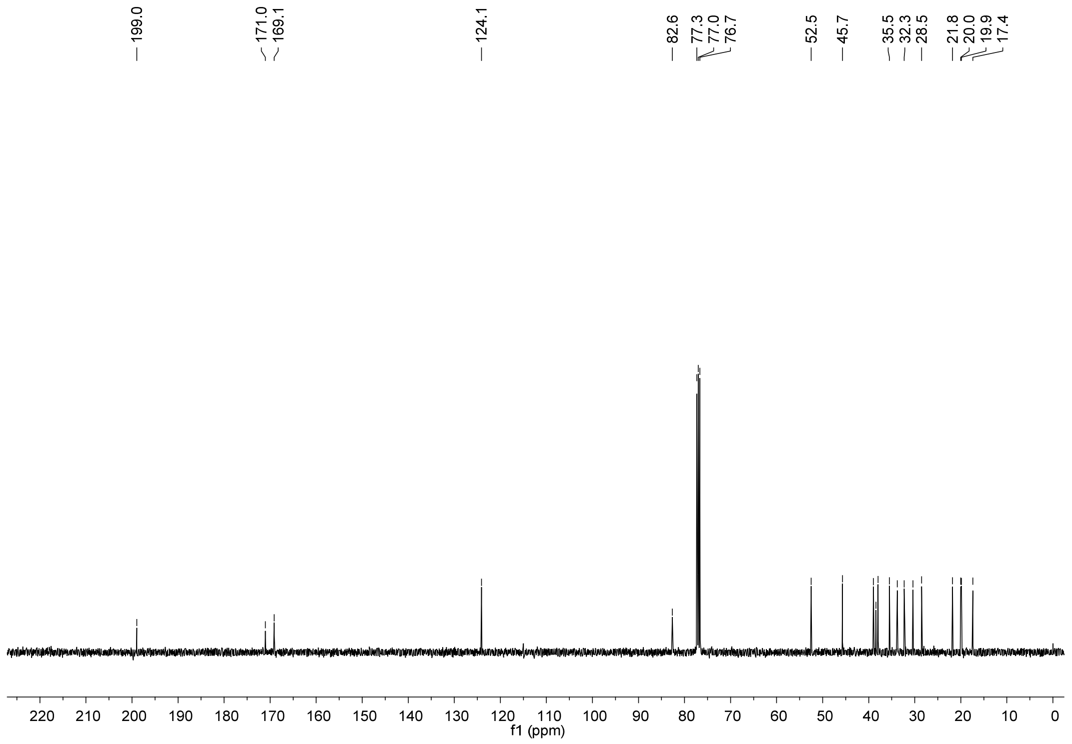

Supplementary Figure 71. The ^{1}H NMR and ^{13}C NMR spectra of 24.

(**a**) ^{1}H NMR spectrum in $CDCl_3$ at 400 MHz; (**b**) ^{13}C NMR spectrum in $CDCl_3$ at 100 MHz.

Supplementary Tables

Supplementary Table 1. The genes and primers used in this study for RT-PCR. Sequence data for the genes have been deposited at GenBank.

Gene	GenBank	Primer	Primer Sequence (5' to 3')	Primer Usage
GAPDH	KY977747	GAPDH-F	AATGGCAAGCTCACCGGAATG	RT-PCR analysis of *GAPDH*
		GAPDH-R	GTTGGTGTTGCCGTTCAAGTC	
g2188	MG886385	g2188-F	ACGCATGGCAGTCGACTCTG	RT-PCR analysis of *g2188*
		g2188-R	CTCGAGACGGTCCTTCACGC	
g2592	MG886388	g2592-F	CGCGTAACTGCATCGGAATGG	RT-PCR analysis of *g2592*
		g2592-R	GGATGCACCGTGCCGTTTC	
g2700	MG886389	g2700-F	TGTTCCCGCACCTCCTCAATC	RT-PCR analysis of *g2700*
		g2700-R	GAGAACTGACGCATTCACGTTG	
g3099	MG886390	g3099-F	TACGCGATATCTTGACGCCG	RT-PCR analysis of *g3099*
		g3099-R	GCCAGATCTCGGGGTTCTTG	
g3270 (*vidE*)	MG886384	g3270-F	CCAACACCGCAAGTAACCTTG	RT-PCR analysis of *g3270*
		g3270-R	CCAACACCGCAAGTAACCTTG	
g3340	MG886391	g3340-F	TAGTCGCGCTCGTTCCTGG	RT-PCR analysis of *g3340*
		g3340-R	CGCTGAAGGGACTTTCGGC	
g3939	MG886392	g3939-F	AGGAAGTACGCCGCTTGGAG	RT-PCR analysis of *g3939*
		g3939-R	TCGGGATTGCGGTTCATACTG	
g8806	MG886393	g8806-F	TGGTCGTGGGAAGACAAGCG	RT-PCR analysis of *g8806*
		g8806-R	CCCGGTTTGTCGCGTTGTCT	
g11474	MG886395	g11474-F	AGATACCCGGGCCAGTGCTAG	RT-PCR analysis of *g11474*
		g11474-R	CGAACCTCGGCTGCAAGATG	
g12398	MG886396	g12398-F	CTGCTTGCGGCGATGATGAG	RT-PCR analysis of *g12398*
		g12398-R	TCAGCTGCGAGAGGTGCTC	
g12726	MG886397	g12726-F	AGGAAGCTGGTGGAATGGGC	RT-PCR analysis of *g12726*
		g12726-R	TTGACGAACTCGTCGGCCC	
g13550	MG886398	g13550-F	ACTCCCTCCAAAACCCCGAG	RT-PCR analysis of *g13550*
		g13550-R	AGTGTTGTTCTCGGCTTGCTC	
g2588	MG886386	g2588-F	AACGTCGTGCGAATCTCCG	RT-PCR analysis of *g2588*
		g2588-R	TAGCCGTATGGCGGAGGAC	
g2590	MG886387	g2590-F	AATGGCACCTTCAAGCGTG	RT-PCR analysis of *g2590*
		g2590-R	TTGGTCTCCGGGAATCGAG	
g11473	MG886394	g11473-F	ATCCTTCTCGAGTCCAGGGG	RT-PCR analysis of *g11473*
		g11473-R	AGTTCCCCGAGACAGTCGAAG	
g3266 (*vidA*)	MG886384	g3266-F	TCGCAGCATCTGGGTATGAC	RT-PCR analysis of *g3266*
		g3266-R	ACGCAGAGGTTACATGTGCC	
g3269 (*vidD*)	MG886384	g3269-F	ACAGACACGCAGGATGAAGG	RT-PCR analysis of *g3269*
		g3269-R	TGTCCCTGTAGTATCGTGGGC	
g3271 (*vidF*)	MG886384	g3271-F	ATACATTGGGAGGCCACCG	RT-PCR analysis of *g3271*
		g3271-R	TGTGGTCGAGTCACAAGCAC	
g3272 (*vidG*)	MG886384	g3272-F	ATCCAGCACGAGTGGGAGAC	RT-PCR analysis of *g3272*
		g3272-R	TGAGCACGATGCACATGGC	

g3275 (*vidJ*)	MG886384	g3275-F	TGCCCAGACTGATTTGCCG	RT-PCR analysis of *g3275*
		g3275-R	TGCAGAACCCCAAGTGCAAG	
g3276 (*vidK*)	MG886384	g3276-F	AACGGCCTTGGAGTGGTGG	RT-PCR analysis of *g3276*
		g3276-R	TCGACGATTTCGTACGCCGG	
g3278 (*vidM*)	MG886384	g3278-F	TTCTCCGGCTTCATGAGGGC	RT-PCR analysis of *g3278*
		g3278-R	TGCGGACTCGGCTACCTTC	
g3281 (*vidP*)	MG886384	g3281-F	TGACCAGGCCGAGATTACTG	RT-PCR analysis of *g3281*
		g3281-R	TTAGAGCCGCGTAGACGTC	
g3283 (*vidR*)	MG886384	g3283-F	CCGTTGCTCTTCATGAGCTG	RT-PCR analysis of *g3283*
		g3283-R	CTGAGCGCGAAGATCTCGTG	

Supplementary Table 2. Primers used for constructing recombinant plasmids. Restriction sites are indicated by bold letters, target sequences of genes controlled by T7 promoter are underlines.

Primer	Sequence (5' to 3')	Usage
PtrpC-XbaI-F	GC**TCTAGA**GCGCAATTAACCCTCACTAA	Amplification of PtrPC-*neo*-TtrPC from pBSKII-PtrPC-*neo*-TtrPC plasmid.
TtrpC-HindIII-R	CCC**AAGCTT**CAGGGCTGGTGACGGAATTTTCATAG	
gRNA-g3262-F	TAATACGACTCACTATAGGTGCTGGTATCAAGGCGTCGTTTTAGAGCTAGAAATAGC	Cloning of gRNA cassette for construction of pUCm-gRNA-*g3262*
gRNA-g3264-F	TAATACGACTCACTATAGGAGAAGTACCCGGTCTCCGGTTTTAGAGCTAGAAATAGC	Cloning of gRNA cassette for construction of pUCm-gRNA-*g3264*
gRNA-g3265-F	TAATACGACTCACTATAGGAATCTCTCTGTGTTGAGAGTTTTAGAGCTAGAAATAGC	Cloning of gRNA cassette for construction of pUCm-gRNA-*g3265*
gRNA-g3285-F	TAATACGACTCACTATAGGGATGAGCCGAGAGTAATCGTTTTAGAGCTAGAAATAGC	Cloning of gRNA cassette for construction of pUCm-gRNA-*g3285*
gRNA-vidA-F	TAATACGACTCACTATAGGACAGCGTGAATTGCTGGTGTTTTAGAGCTAGAAATAGC	Cloning of gRNA cassette for construction of pUCm-gRNA-*vidA*
gRNA-vidD-F	TAATACGACTCACTATAGGTTTCGGCACTTCCCGTGAGTTTTAGAGCTAGAAATAGC	Cloning of gRNA cassette for construction of pUCm-gRNA-*vidD*
gRNA-vidE-F	TAATACGACTCACTATAGGTTGCCTATGTCGTGACTAGTTTTAGAGCTAGAAATAGC	Cloning of gRNA cassette for construction of pUCm-gRNA-*vidE*
gRNA-vidF-F	TAATACGACTCACTATAGGTCCTTCTCATACTGTTCGGTTTTAGAGCTAGAAATAGC	Cloning of gRNA cassette for construction of pUCm-gRNA-*vidF*
gRNA-vidG-F	TAATACGACTCACTATAGGTACGATTTCCTAAGCTGTGTTTTAGAGCTAGAAATAGC	Cloning of gRNA cassette for construction of pUCm-gRNA-*vidG*
gRNA-vidH-F	TAATACGACTCACTATAGGTCATAGCAGACGTCAATCGTTTTAGAGCTAGAAATAGC	Cloning of gRNA cassette for construction of pUCm-gRNA-*vidH*
gRNA-vidJ-F	TAATACGACTCACTATAGGGCTCTGAGGTGCTTATGTGTTTTAGAGCTAGAAATAGC	Cloning of gRNA cassette for construction of pUCm-gRNA-*vidJ*
gRNA-vidK-F	TAATACGACTCACTATAGGAGCCAATGCGCCAAGACCGTTTTAGAGCTAGAAATAGC	Cloning of gRNA cassette for construction of pUCm-gRNA-*vidK*

gRNA-vidM-F	TAATACGACTCACTATAGGAGAACCAGGTGCATGGTAGTTTTAGAGCTAGAAATAGC	Cloning of gRNA cassette for construction of pUCm-gRNA-*vidM*
gRNA-vidN-F	TAATACGACTCACTATAGGGCCGTTGCCTTCCCCATGGTTTTAGAGCTAGAAATAGC	Cloning of gRNA cassette for construction of pUCm-gRNA-*vidN*
gRNA-vidO-F	TAATACGACTCACTATAGGTCGGCGTACGGACCTCCCGTTTTAGAGCTAGAAATAGC	Cloning of gRNA cassette for construction of pUCm-gRNA-*vidO*
gRNA-vidP-F	TAATACGACTCACTATAGGGAGGCGGCATCGCTGCGTGTTTTAGAGCTAGAAATAGC	Cloning of gRNA cassette for construction of pUCm-gRNA-*vidP*
gRNA-vidQ-F	TAATACGACTCACTATAGGAGCATAGTGTGGTTGACCGTTTTAGAGCTAGAAATAGC	Cloning of gRNA cassette for construction of pUCm-gRNA-*vidQ*
gRNA-vidR-F	TAATACGACTCACTATAGGCGCGGAATCCGTCGAACCGTTTTAGAGCTAGAAATAGC	Cloning of gRNA cassette for construction of pUCm-gRNA-*vidR*
gRNA-vidS-F	TAATACGACTCACTATAGGCACGGATACCCCGCTCCCGTTTTAGAGCTAGAAATAGC	Cloning of gRNA cassette for construction of pUCm-gRNA-*vidS*
eGFP-R	TTACACCTTCCTCTTCTTC	Cloning of the gRNA cassettes
pUCm-F	TCGCGCGTTTCGGTGATGAC	Amplification of transcription templates
gRNA-R	AAAAGCACCGACTCGGTGCC	
Inf-pTAex3-vidE-F	AGCAAGCTCCGAATTATGGCGGAACCACTCTCCTC	Cloning of *vidE* from *Nodulisporium* sp. (No. 65-12-7-1) genome
Inf-pTAex3-vidE-R	ACTACAGATCCCCGGCTATTGTCCATCTCCAGAGAG	
Inf-pTAex3-vidG-F	AGCAAGCTCCGAATTATGTTCACACCGAAGCCGCA	Cloning of *vidG* from *Nodulisporium* sp. (No. 65-12-7-1) genome
Inf-pTAex3-vidG-R	ACTACAGATCCCCGGTCAGACGGCGGCCGTGGAG	
Inf-pTAex3-vidN-F	AGCAAGCTCCGAATTATGCTTTCGAGACTGCTGGA	Cloning of *vidN* from *Nodulisporium* sp. (No. 65-12-7-1) genome
Inf-pTAex3-vidN-R	ACTACAGATCCCCGGTCAAATACGTGGTGGTTCTA	
Inf-pTAex3-vidO-F	AGCAAGCTCCGAATTATGGCCTCTTATCTTGTCAC	Cloning of *vidO* from *Nodulisporium* sp. (No. 65-12-7-1) genome
Inf-pTAex3-vidO-R	ACTACAGATCCCCGGCTACCAAGGTATCTCGGCC	
Inf-pUSA-vidR-F	TCGAGCTCGGTACCCATGTCCTACGACAAGAATC	Cloning of *vidR* from *Nodulisporium* sp. (No. 65-12-7-1) genome
Inf-pUSA-vidR-R	CTACTACAGATCCCCCTACTCGACATCTAGGAGATC	
Inf-pTAex3-vidF-F	AGCAAGCTCCGAATTATGCATTACGATCTCGATGC	Cloning of *vidF* from *Nodulisporium* sp. (No. 65-12-7-1) genome
Inf-pTAex3-vidF-R	ACTACAGATCCCCGGCTAATTACTGTCTGTTGTCT	
Inf-pTAex3-vidH-F	AGCAAGCTCCGAATTATGTCGGTATTGACACCACC	Cloning of *vidH* from *Nodulisporium* sp. (No. 65-12-7-1) genome
Inf-pTAex3-vidH-R	ACTACAGATCCCCGGTCACAGCAGCAACTTGACAAC	
Inf-pTAex3-vidP-F	AGCAAGCTCCGAATTATGGATGCCGCGCCAAGGC	Cloning of *vidP* from *Nodulisporium* sp. (No. 65-12-7-1) genome
Inf-pTAex3-vidP-R	ACTACAGATCCCCGGTCACATACTTGTCAGTGCTC	
Inf-pAdeA-Pamy-F	GCAGGTCGACTCTAGACGACTCCAATCTTCAAGAGC	Construction of recombinant pAdeA plasmids containing two or three exogenous genes using the In-Fusion® HD Cloning Kit
Inf-pAdeA-Tamy-R	TAGTAGATCCTCTAGAGTAAGATACATGAGCTTCGG	
Inf-pTAex3-Pamy-F1	GCTCGCGAGCGCGTTCCACTGCATCATCAGTCTAG	
Inf-pTAex3-Tamy-R1	AACGCGCTCGCGAGCAAGTACCATACAGTACCGCG	
Inf-pTAex3-Pamy-F2	TCGCGTGCGCGTTTACCCATCATGGTGTTTTGATC	
Inf-pTAex3-Tamy-R2	TAAACGCGCACGCGACATTAATCCGGATCCTTTCC	
VidF-NdeI-F	GGAATTC**CATATG**ATGCATTACGATCTCGATGC	Cloning of *vidF* from *Nodulisporium* sp. cDNA for construction of pET28a-*vidF*
VidF-NotI-R	AAGGAAAAAAG**CGGCCG**CCTAATTACTGTCTGTTGTCTTC	

Supplementary Table 3. Plasmids used in this study

Plasmids	Characteristics	Source
pBSKII-PtrPC-*neo*-TtrPC	Plasmid containing PtrPC-*neo*-TtrPC maker cassette, (*Amp*^{R})	Zheng, Y. M. *et al.*[1]
pUCm-T	*E. coli* cloning vector, (*Amp*^{R})	Sangon Biotech Co., Ltd.
pUCm-gRNAscaffold-*eGFP*	PUCm-T containing gRNA scaffold, (*Amp*^{R})	Zheng, Y. M. *et al.*[1]
pUCm-gRNA-*g3262*	PUCm-T containing gRNA-*g3262* scaffold, (*Amp*^{R})	This work
pUCm-gRNA-*g3264*	PUCm-T containing gRNA-*g3264* scaffold, (*Amp*^{R})	This work
pUCm-gRNA-*g3265*	PUCm-T containing gRNA-*g3265* scaffold, (*Amp*^{R})	This work
pUCm-gRNA-*g3285*	PUCm-T containing gRNA-*g3285* scaffold, (*Amp*^{R})	This work
pUCm-gRNA-*vidA*	PUCm-T containing gRNA-*vidA* scaffold, (*Amp*^{R})	This work
pUCm-gRNA-*vidD*	PUCm-T containing gRNA-*vidD* scaffold, (*Amp*^{R})	This work
pUCm-gRNA-*vidE*	PUCm-T containing gRNA-*vidE* scaffold, (*Amp*^{R})	This work
pUCm-gRNA-*vidF*	PUCm-T containing gRNA-*vidF* scaffold, (*Amp*^{R})	This work
pUCm-gRNA-*vidG*	PUCm-T containing gRNA-*vidG* scaffold, (*Amp*^{R})	This work
pUCm-gRNA-*vidH*	PUCm-T containing gRNA-*vidH* scaffold, (*Amp*^{R})	This work
pUCm-gRNA-*vidJ*	PUCm-T containing gRNA-*vidJ* scaffold, (*Amp*^{R})	This work
pUCm-gRNA-*vidK*	PUCm-T containing gRNA-*vidK* scaffold, (*Amp*^{R})	This work
pUCm-gRNA-*vidM*	PUCm-T containing gRNA-*vidM* scaffold, (*Amp*^{R})	This work
pUCm-gRNA-*vidN*	PUCm-T containing gRNA-*vidN* scaffold, (*Amp*^{R})	This work
pUCm-gRNA-*vidO*	PUCm-T containing gRNA-*vidO* scaffold, (*Amp*^{R})	This work
pUCm-gRNA-*vidP*	PUCm-T containing gRNA-*vidP* scaffold, (*Amp*^{R})	This work
pUCm-gRNA-*vidQ*	PUCm-T containing gRNA-*vidQ* scaffold, (*Amp*^{R})	This work
pUCm-gRNA-*vidR*	PUCm-T containing gRNA-*vidR* scaffold, (*Amp*^{R})	This work
pUCm-gRNA-*vidS*	PUCm-T containing gRNA-*vidS* scaffold, (*Amp*^{R})	This work
pTAex3	Plasmid containing *argB* maker gene cassette for gene expression in *A. oryzae* NSAR1, (*Amp*^{R})	Fujii, T. *et al.*[2]
pAdeA	Plasmid containing *adeA* maker gene cassette for gene expression in *A. oryzae* NSAR1, (*Amp*^{R})	Jin, F. J. *et al.*[3]
pTAex3-*vidE*	pTAex3 containing *vidE* whose expression is regulated by *amyB* promoter, (*Amp*^{R})	This work
pTAex3-*vidG*	pTAex3 containing *vidG* whose expression is regulated by *amyB* promoter, (*Amp*^{R})	This work
pTAex3-*vidN*	pTAex3 containing *vidN* whose expression is regulated by *amyB* promoter, (*Amp*^{R})	This work
pTAex3-*vidO*	pTAex3 containing *vidO* whose expression is regulated by *amyB* promoter, (*Amp*^{R})	This work
pUSA-*vidR*	pUSA containing *vidR* whose expression is regulated by *amyB* promoter, (*Amp*^{R})	This work
pAdeA-*vidE*-*vidN*-*vidO*	pAdeA containing *vidE, vidN*, and *vidO* whose expressions are independently regulated by *amyB* promoter, (*Amp*^{R})	This work
pTAex3-*vidF*	pTAex3 containing *vidF* whose expression is regulated by *amyB* promoter, (*Amp*^{R})	This work
pTAex3-*vidH*	pTAex3 containing *vidH* whose expression is regulated by *amyB* promoter, (*Amp*^{R})	This work
pTAex3-*vidP*	pTAex3 containing *vidP* whose expression is regulated by *amyB* promoter, (*Amp*^{R})	This work
pAdeA-*vidH*-*vidP*	pAdeA containing *vidH* and *vidP* whose expressions are independently regulated by *amyB* promoter, (*Amp*^{R})	This work
pET-28a (+)	Plasmid for gene expression in *E. coli* BL21-Codon Plus (DE3)	Novagen
pET28a-*vidF*	pET-28a (+) containing *vidF* whose expression is regulated by T7 promoter, (*Kan*^{R})	This work

Supplementary Table 4. Strains used in the study

**Strains**	**Characteristics**	**Source**
E. coli DH5α	Host for general plasmid cloning	TaKaRa
E. coli BL21–Codon Plus (DE3)	Host for gene expression	TaKaRa
A. oryzae NSAR1	Host for gene expression, a quadruple auxotrophic mutant strain (*niaD*$^{-}$, *sC*$^{-}$, *ΔargB*, *adeA*$^{-}$)	Jin, F. J. *et al.*[3]
JA1	*A. oryzae* NSAR1 transformant harboring *vidE*, *vidG, vidN*, *vidO,* and *vidR*	This work
JA2	*A. oryzae* NSAR1 transformant harboring *vidP*	This work
JA3	*A. oryzae* NSAR1 transformant harboring *vidH*	This work
JA4	*A. oryzae* NSAR1 transformant harboring *vidF*, *vidH* and *vidP*	This work
Nodulisporium sp. (No. 65-12-7-1)	Wild-type strain, viridins producing	Lab stock
JN1001	Cas9-expressing *Nodulisporium* sp. (No. 65-12-7-1)	Zheng, Y. M. *et al.*[1]
Δ*g3262*-JN1001	*g3262* deletion mutant of JN1001	This work
Δ*g3264*-JN1001	*g3264* deletion mutant of JN1001	This work
Δ*g3265*-JN1001	*g3265* deletion mutant of JN1001	This work
Δ*g3285*-JN1001	*g3285* deletion mutant of JN1001	This work
Δ*vidA*-JN1001	*vidA* deletion mutant of JN1001	This work
Δ*vidD*-JN1001	*vidD* deletion mutant of JN1001	This work
Δ*vidE*-JN1001	*vidE* deletion mutant of JN1001	This work
Δ*vidF*-JN1001	*vidF* deletion mutant of JN1001	This work
Δ*vidG*-JN1001	*vidG* deletion mutant of JN1001	This work
Δ*vidH*-JN1001	*vidH* deletion mutant of JN1001	This work
Δ*vidJ*-JN1001	*vidJ* deletion mutant of JN1001	This work
Δ*vidK*-JN1001	*vidK* deletion mutant of JN1001	This work
Δ*vidM*-JN1001	*vidM* deletion mutant of JN1001	This work
Δ*vidN*-JN1001	*vidN* deletion mutant of JN1001	This work
Δ*vidO*-JN1001	*VidO* deletion mutant of JN1001	This work
Δ*vidP*-JN1001	*vidP* deletion mutant of JN1001	This work
Δ*vidQ*-JN1001	*vidQ* deletion mutant of JN1001	This work
Δ*vidR*-JN1001	*vidR* deletion mutant of JN1001	This work
Δ*vidS*-JN1001	*vidS* deletion mutant of JN1001	This work

Supplementary Table 5. Differential expression analysis of cluster V

The expression levels were quantified using the *DESeq* with a FDR< 0.05 and an absolute value of fold-change ≥2. M/C means Maltose medium (M) in comparison to Czapek medium (C), the + values indicate the expressions of the genes are up-regulated under the Maltose medium, – values indicate the expressions are down-regulated, and Non means unexpressed; RPKM means Reads Per Kilobase of exon per Million mapped reads.

**Gene**	**Expression** (RPKM)		**Log$_2$Fold Change**	**Gene**	**Expression** (RPKM)		**Log$_2$Fold Change**
	M	C	(M/C)		M	C	(M/C)
g3260	3.8	3.2	-4.3	*g3273*	913.5	17.4	5.7
g3261	Non	Non	Non	*g3274*	383.6	6.0	6.0
g3262	217.4	54.6	2.0	*g3275*	1014.2	30.5	5.1
g3263	Non	Non	Non	*g3276*	593.7	16.4	5.2
g3264	17.2	1.8	3.3	*g3277*	245.9	18.3	3.7
g3265	9.3	0.8	3.6	*g3278*	65.4	12.8	2.4
g3266	918.3	12.0	6.3	*g3279*	305.2	5.4	5.8
g3267	343.8	22.9	3.9	*g3280*	1074.4	92.5	3.5
g3268	343.8	22.9	3.9	*g3281*	369.4	15.6	4.6
g3269	1064.5	21.7	5.6	*g3282*	752.7	13.7	5.8
g3270	1963.0	33.7	5.9	*g3283*	752.7	13.7	5.8
g3271	1158.0	17.5	6.1	*g3284*	103.5	16.3	2.7
g3272	913.5	17.4	5.7	*g3285*	0.5	4.0	-2.9

Supplementary Table 6. NMR data for 2 (400 MHz for ^{1}H and 100 MHz for ^{13}C, in DMSO-d_6)

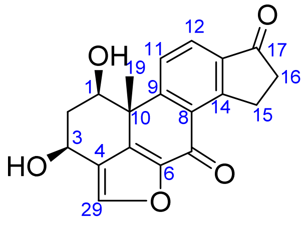

Demethoxyviridiol (2)

position	δ_C, type	δ_H (J in Hz)[a]	^{1}H–^{1}H COSY	HMBC	ROESY
1	70.5, CH	4.02, ddd (11.6, 5.3, 4.2)	2a, 2b, 1-OH		3, 11
2	40.7, CH_2	a: 2.36, ddd (13.3, 7.5, 4.2)	1, 2b, 3	1, 3, 4, 10	1-OH
		b: 2.14, ddd (13.3, 11.6, 9.2)	1, 2a, 3	1, 3, 4, 10	19, 1-OH
3	59.7, CH	4.79	2a, 2b, 3-OH, 29	4	1
4	125.8, C				
5	144.3, C				
6	144.3, C				
7	172.7, C				
8	130.0, C				
9	157.9, C				
10	41.9, C				
11	128.0, CH	8.60, d (8.2)	12	8, 9, 10, 13	1, 1-OH
12	125.9, CH	7.83, d (8.2)	11	9, 11, 14, 17	
13	136.2, C				
14	156.9, C				
15	28.1, CH_2	a: 3.62	15b, 16	14	
		b: 3.52	15a, 16	14	
16	35.8, CH_2	2.63	15a, 15b	14, 17	
17	205.7, C				
19	27.6, CH_3	1.51, s		1, 5, 9, 10	2b, 1-OH
29	146.5, CH	8.05, br s	3	4, 5, 6	
1-OH		5.76, d (5.3)	1	10	2a, 2b, 11, 19
3-OH		5.51, d (5.2)	3		

[a] The indiscernible signals due to overlap or complex multiplicity are reported without designating multiplicity.

Supplementary Table 7. NMR data for 3 (400 MHz for ^1H and 100 MHz for ^{13}C, in DMSO-d_6)

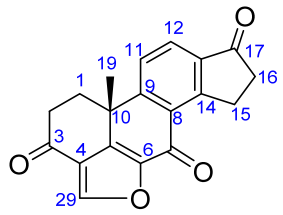

Dehydroxydemethoxyviridin (3)

position	δ_C, type	δ_H (J in Hz)[a]	^1H–^1H COSY	HMBC
1	32.9, CH_2	a: 2.82, br dd (13.3, 5.4)	1b, 2a, 2b	2, 3, 5, 10
		b: 2.16, td (13.3, 4.7)	1a, 2a, 2b	2, 10, 19
2	36.4, CH_2	a: 3.09, ddd (18.5, 13.3, 5.4)	1a, 1b, 2b	1, 3
		b: 2.65	1a, 1b, 2a	3, 10
3	191.7, C			
4	122.1, C			
5	146.3, C			
6	144.6, C			
7	172.8, C			
8	129.7, C			
9	157.0, C			
10	36.5, C			
11	126.1, CH	7.91, d (7.9)	12	8, 10, 13
12	126.7, CH	7.93, d (7.9)	11	9, 14, 17
13	136.7, C			
14	157.5, C			
15	28.0, CH_2	a: 3.68	15b, 16	
		b: 3.56	15a, 16	
16	35.9, CH_2	2.69	15a, 15b	
17	205.4, C			
19	30.2, CH_3	1.62, s		1, 5, 9, 10
29	149.9, CH	8.80, s		4, 5, 6

[a] The indiscernible signals due to overlap or complex multiplicity are reported without designating multiplicity.

Supplementary Table 8. NMR data for 4 (400 MHz for ^{1}H and 100 MHz for ^{13}C, in DMSO-d_6)

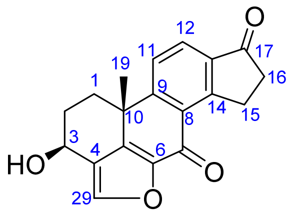

Nodulisporiviridin I (4)

position	δ_C, type	δ_H (J in Hz)[a]	^{1}H–^{1}H COSY	HMBC	ROESY
1	32.1, CH_2	a: 2.54	1b, 2a, 2b	2, 3, 5, 10	11, 19
		b: 1.72, td (12.8, 2.4)	1a, 2a, 2b	2, 10, 19	3
2	30.2, CH_2	a: 2.28	1a, 1b, 2b, 3	3, 10	
		b: 2.06	1a, 1b, 2a, 3	1, 3, 10	19
3	60.8, CH	4.74, br t (7.8)	2a, 2b, 29	2, 4, 5	1b
4	125.8, C				
5	145.7, C				
6	143.6, C				
7	172.7, C				
8	130.0, C				
9	158.3, C				
10	36.9, C				
11	125.2, CH	7.76, d (7.8)	12	8, 10, 13	1a
12	126.2, CH	7.83, d (7.8)	11	9, 14, 17	
13	136.3, C				
14	157.3, C				
15	27.9, CH_2	a: 3.63	15b, 16	14	
		b: 3.49	15a, 16	14	
16	35.9, CH_2	2.63	15a, 15b	17	
17	205.5, C				
19	33.2, CH_3	1.51, s		1, 5, 9, 10	1a, 2b
29	146.9, CH	8.03, br s	3	4, 5, 6	

[a] The indiscernible signals due to overlap or complex multiplicity are reported without designating multiplicity.

Supplementary Table 9. NMR data for 5 (400 MHz for ^{1}H and 100 MHz for ^{13}C, in CD_3OD)

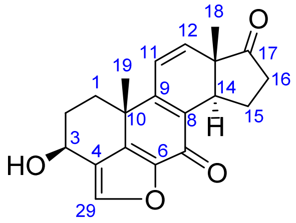

Nodulisporiviridin J (5)

position	δ_C, type	δ_H (J in Hz)[a]	^{1}H–^{1}H COSY	HMBC	NOESY
1	32.3, CH_2	a: 2.21	1b, 2a, 2b	2, 3, 5	11
		b: 1.53	1a, 2a, 2b	2, 5, 19	
2	31.1, CH_2	a: 2.35	1a, 1b, 2b, 3		
		b: 2.13	1a, 1b, 2a, 3	1	
3	62.4, CH	4.86	2a, 2b, 29	4, 5	
4	126.2, C				
5	147.8, C				
6	146.0, C				
7	176.3, C				
8	131.9, C				
9	159.5, C				
10	38.7, C				
11	125.0, CH	6.46, d (8.1)	12	8, 9, 10, 12, 13	1a, 19
12	141.3, CH	6.68, d (8.1)	11	9, 11, 13, 14, 17	18
13	51.3, C				
14	44.6, CH	2.84	15a, 15b	8, 9, 12, 15, 17, 18	
15	22.5, CH_2	a: 2.82	14, 15b, 16a, 16b	8, 13, 14, 17	
		b: 2.36	14, 15a, 16a, 16b	16, 17	
16	38.2, CH_2	a: 2.59	15a, 15b, 16b	14, 17	
		b: 2.36	15a, 15b, 16a	15, 17	
17	216.5, C				
18	12.9, CH_3	0.89, s		12, 13, 14, 17	12, 19
19	29.7, CH_3	1.53, s		1, 5, 9, 10	11, 18
29	147.4, CH	7.80, br s	3	4, 5, 6	

[a] The indiscernible signals due to overlap or complex multiplicity are reported without designating multiplicity.

Supplementary Table 10. NMR data for 6 (300 MHz for ^{1}H and 75 MHz for ^{13}C, in DMSO-d_6)

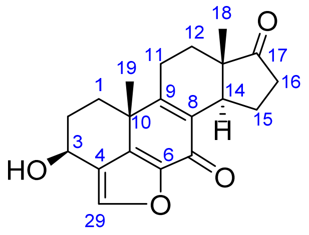

Nodulisporiviridin K (6)

position	δ_C, type	δ_H (J in Hz)[a]	^{1}H–^{1}H COSY[b]	HMBC[b]	NOESY[b]
1	31.1, CH_2	a: 1.94	1b, 2a, 2b	3, 5, 10, 19	19
		b: 1.48	1a, 2a, 2b	3, 5, 19	3
2	30.1, CH_2	a: 2.17	1a, 1b, 2b, 3	1, 3, 4, 10	
		b: 1.90	1a, 1b, 2a, 3	1, 3	19
3	60.6, CH	4.66, br t (7.9)	2a, 2b, 3-OH, 29	2, 4, 5	1b
4	125.3, C				
5	146.5, C				
6	143.5, C				
7	174.0, C				
8	131.7, C				
9	163.4, C				
10	38.2, C				
11	23.9, CH_2	2.57	12a, 12b	8, 9, 10, 12, 13	18, 19
12	27.3, CH_2	a: 1.75	11, 12b	9, 11, 13, 14, 17, 18	18
		b: 1.45	11, 12a	11, 13, 17, 18	
13	47.4, C				
14	43.1, CH	2.57	15a, 15b	8, 13, 15, 18	
15	22.8, CH_2	a: 2.88	14, 15b, 16a, 16b	13, 14, 16, 17	
		b: 1.88	14, 15a, 16a, 16b	8, 14, 16	18
16	36.2, CH_2	a: 2.48	15a, 15b, 16b	14, 15, 17	
		b: 2.16	15a, 15b, 16a	15, 17	
17	218.3, C				
18	13.3, CH_3	0.79, s		12, 13, 14, 17	11, 12a, 15b, 19
19	29.4, CH_3	1.51, s		1, 5, 9, 10	1a, 2b, 11, 18
29	145.4, CH	7.89, d (1.1)	3	4, 5, 6	
3-OH		5.42, br s	3		

[a] The indiscernible signals due to overlap or complex multiplicity are reported without designating multiplicity.

[b] The data were recorded with a Bruker AV 400 spectrometer.

Supplementary Table 11. NMR data for 7 (400 MHz for ^{1}H and 100 MHz for ^{13}C, in DMSO-d_6)

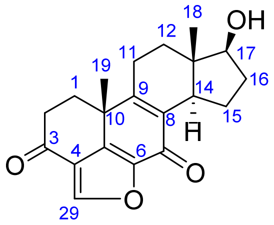

Nodulisporiviridin L (7)

position	δ_C, type	δ_H (J in Hz)[a]	^{1}H–^{1}H COSY	HMBC	ROESY
1	31.8, CH_2	a: 2.22, ddd (13.2, 5.3, 1.4)	1b, 2a, 2b	3, 5	19
		b: 1.89	1a, 2a, 2b	2, 19	
2	36.3, CH_2	a: 2.91, ddd (18.7, 13.3, 5.3)	1a, 1b, 2b	3	19
		b: 2.50	1a, 1b, 2a	1, 3, 10	
3	191.9, C				
4	121.7, C				
5	146.8, C				
6	144.7, C				
7	174.0, C				
8	133.6, C				
9	162.1, C				
10	37.5, C				
11	24.3, CH_2	2.50	12a, 12b	8, 9, 10, 12, 13	
12	31.9, CH_2	a: 1.87	11, 12b	9, 11, 13, 14	18
		b: 1.28	11, 12a	11, 13, 14, 17, 18	14, 17
13	43.0, C				
14	43.0, CH	2.17	15a, 15b	8, 9, 13, 15, 18	12b, 17
15	24.2, CH_2	a: 2.50	14, 15b, 16a, 16b	13, 14, 17	
		b: 1.53	14, 15a, 16a, 16b		
16	30.6, CH_2	a: 1.98	15a, 15b, 16b, 17		
		b: 1.46	15a, 15b, 16a, 17	15, 17	
17	77.5, CH	3.58, t (8.3)	16a, 16b	12, 18	12b, 14
18	10.9, CH_3	0.65, s		12, 13, 14, 17	12a, 19
19	27.0, CH_3	1.54, s		1, 5, 9, 10	1a, 2a, 18
29	148.1, CH	8.60, s		4, 5, 6	

[a] The indiscernible signals due to overlap or complex multiplicity are reported without designating multiplicity.

Supplementary Table 12. NMR data for 8 (400 MHz for ^{1}H and 100 MHz for ^{13}C, in DMSO-d_6)

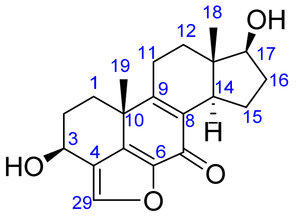

Nodulisporiviridin M (8)

position	δ_C, type	δ_H (J in Hz)[a]	^{1}H–^{1}H COSY	HMBC	ROESY
1	31.3, CH_2	a: 1.92	1b, 2a, 2b	3, 5	19
		b: 1.45	1a, 2a, 2b	19	3
2	30.1, CH_2	a: 2.13	1a, 1b, 2b, 3	3, 4, 10	
		b: 1.88	1a, 1b, 2a, 3	1, 3, 10	
3	60.6, CH	4.64, br t (7.8)	2a, 2b, 3-OH, 29	2, 4, 5	1b
4	125.2, C				
5	146.3, C				
6	143.6, C				
7	174.2, C				
8	133.2, C				
9	162.6, C				
10	37.9, C				
11	24.3, CH_2	2.49	12a, 12b	8, 9, 12, 13	19
12	31.9, CH_2	a: 1.83	11, 12b	9, 13, 14	18
		b: 1.23	11, 12a	11, 13, 14, 17, 18	14, 17
13	43.0, C				
14	43.0, CH	2.10	15a, 15b	8, 9, 13, 15, 18	12b, 17
15	24.2, CH_2	a: 2.49	14, 15b, 16a, 16b	8, 13, 14, 17	
		b: 1.53	14, 15a, 16a, 16b		
16	30.7, CH_2	a: 1.95	15a, 15b, 16b, 17		
		b: 1.45	15a, 15b, 16a, 17	15, 17	
17	77.6, CH	3.56,br t (7.9)	16a, 16b, 17-OH	12, 18	12b, 14
18	10.9, CH_3	0.64, s		12, 13, 14, 17	12a, 19
19	29.5, CH_3	1.49, s		1, 5, 9, 10	1a, 11, 18
29	145.0, CH	7.84, br s	3	4, 5, 6	
3-OH		5.37, br s	3		
17-OH		4.55, br s	17		

[a] The indiscernible signals due to overlap or complex multiplicity are reported without designating multiplicity.

Supplementary Table 13. NMR data for 9 (400 MHz for ^{1}H and 100 MHz for ^{13}C, in DMSO-d_6)

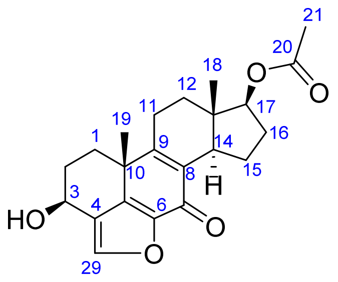

Nodulisporiviridin N (9)

position	δ_C, type	δ_H (J in Hz)[a]	^{1}H–^{1}H COSY	HMBC	NOESY
1	31.2, CH_2	a: 1.92	1b, 2a, 2b	3, 5, 10, 19	19
		b: 1.46	1a, 2a, 2b	3, 5, 10, 19	3
2	30.1, CH_2	a: 2.15	1a, 1b, 2b, 3	3, 4, 10	
		b: 1.89	1a, 1b, 2a, 3	3	
3	60.6, CH	4.65	2a, 2b, 29	1, 2, 4, 5	1b
4	125.2, C				
5	146.5, C				
6	143.5, C				
7	174.0, C				
8	132.4, C				
9	162.7, C				
10	38.0, C				
11	24.0, CH_2	2.49	12a, 12b	8, 9, 10, 12, 13	18, 19
12	31.7, CH_2	a: 1.75	11, 12b	9, 11, 13, 14, 18	18
		b: 1.43	11, 12a	11, 13, 17, 18	14, 17
13	42.6, C				
14	42.7, CH	2.30	15a, 15b	8, 13, 15, 17, 18	12b, 17
15	24.3, CH_2	a: 2.58	14, 15b, 16a, 16b	13, 14, 17	
		b: 1.63	14, 15a, 16a, 16b	8, 14, 16	18
16	28.1, CH_2	a: 2.17	15a, 15b, 16b, 17	13, 14, 17	
		b: 1.55	15a, 15b, 16a, 17	13, 14, 15, 17	18
17	79.9, CH	4.65	16a, 16b	12, 13, 16, 18, 20	12b, 14
18	11.6, CH_3	0.75, s		12, 13, 14, 17	11, 12a, 15b, 16b, 19, 21
19	29.6, CH_3	1.50, s		1, 5, 9, 10	1a, 11, 18
20	170.4, C				
21	20.9, CH_3	2.02, s		17, 20	18
29	145.2, CH	7.86, br s	3	4, 5, 6	

[a] The indiscernible signals due to overlap or complex multiplicity are reported without designating multiplicity.

Supplementary Table 14. NMR data for 10 (600 MHz for ^{1}H and 150 MHz for ^{13}C, in DMSO-d_6)

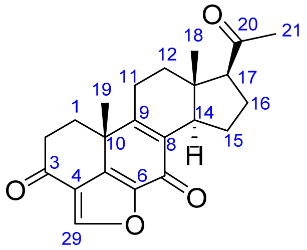

Virone (10)

position	δ_C, type	δ_H (J in Hz)[a]	^{1}H–^{1}H COSY	HMBC	ROESY
1	31.9, CH_2	a: 2.24, ddd (13.3, 5.4, 2.1)	1b, 2a, 2b	2, 3, 5, 10, 19	19
		b: 1.91, td (13.3, 4.7)	1a, 2a, 2b	2, 3, 5, 9, 10, 19	
2	36.3, CH_2	a: 2.91, ddd (18.5, 13.3, 5.4)	1a, 1b, 2b	1, 3	19
		b: 2.54	1a, 1b, 2a	1, 3, 4, 10	
3	191.9, C				
4	121.7, C				
5	146.8, C				
6	144.7, C				
7	173.8, C				
8	133.3, C				
9	162.0, C				
10	37.5, C				
11	24.5, CH_2	2.59	12a, 12b	8, 9, 13	18, 19
12	33.1, CH_2	a: 2.13	11, 12b	9, 11, 13, 14, 18	
		b: 1.69	11, 12a	11, 13, 18	14
13	43.5, C				
14	47.8, CH	2.38	15a, 15b	8, 9, 15, 18	12b
15	24.6, CH_2	a: 2.65	14, 15b, 16a, 16b	13, 14, 16	
		b: 1.48	14, 15a, 16a, 16b	8, 14, 16	18, 21
16	23.5, CH_2	a: 2.13	15a, 15b, 16b, 17	13, 14, 15, 17	
		b: 1.77	15a, 15b, 16a, 17	13, 15, 17	
17	59.5, CH	2.65	16a, 16b	12, 13, 16, 18, 20	
18	12.5, CH_3	0.56, s		12, 13, 14, 17	11, 15b, 19, 21
19	27.0, CH_3	1.53, s		1, 5, 9, 10	1a, 2a, 11, 18
20	208.7, C				
21	31.2, CH_3	2.13, s		17, 20	15b, 18
29	148.2, CH	8.62, s		4, 5, 6	

[a] The indiscernible signals due to overlap or complex multiplicity are reported without designating multiplicity.

Supplementary Table 15. NMR data for 13 (400 MHz for ^{1}H and 100 MHz for ^{13}C, in DMSO-d_6)

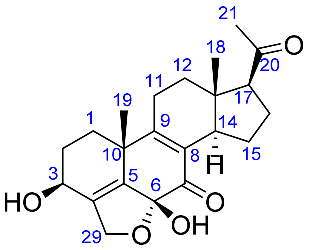

Nodulisporiviridin O (13)

position	δ_C, type	δ_H (J in Hz)[a]	^{1}H–^{1}H COSY	HMBC	ROESY
1	34.4, CH_2	a: 1.79	1b, 2a, 2b	2, 3, 5, 10	19
		b: 1.41	1a, 2a, 2b	3, 19	3
2	29.4, CH_2	a: 1.95	1a, 1b, 2b, 3	1, 3	
		b: 1.63	1a, 1b, 2a, 3	1, 3	
3	63.7, CH	4.07, br t (7.3)	2a, 2b, 3-OH	4, 5, 29	1b, 29a
4	138.8, C				
5	136.6, C				
6	104.6, C				
7	192.7, C				
8	130.6, C				
9	158.7, C				
10	38.0, C				
11	24.0, CH_2	2.39	12a, 12b	8, 9, 12, 13	18, 19
12	33.4, CH_2	a: 2.03	11, 12b	9, 11, 13, 14, 17	
		b: 1.62	11, 12a	13, 18	
13	43.1, C				
14	47.3, CH	2.23	15a, 15b	8, 12, 13, 15, 18	17
15	23.0, CH_2	a: 2.33	14, 15b, 16a, 16b	13, 14, 17	
		b: 1.16, qd (12.3, 7.0)	14, 15a, 16a, 16b	14	18, 21
16	23.1, CH_2	a: 2.06	15a, 15b, 16b, 17	14, 15, 17	
		b: 1.66	15a, 15b, 16a, 17	13, 15, 17	
17	60.0, CH	2.62, t (9.2)	16a, 16b	12, 13, 16, 18, 20	14
18	12.4, CH_3	0.49, s		12, 13, 14, 17	11, 15b, 19, 21
19	23.2, CH_3	1.39, s		1, 5, 9, 10	1a, 11, 18
20	208.5, C				
21	31.1, CH_3	2.09, s		17, 20	15b, 18
29	72.0, CH_2	a: 4.71, d (13.2)	29b	4, 5, 7	3
		b: 4.22, d (13.2)	29a	4, 5, 6	
3-OH		5.06, br s	3	2	
6-OH		6.83, br s		5, 6, 7	

[a] The indiscernible signals due to overlap or complex multiplicity are reported without designating multiplicity.

Supplementary Table 16. NMR data for 14 (400 MHz for ^{1}H and 100 MHz for ^{13}C, in DMSO-d_6)

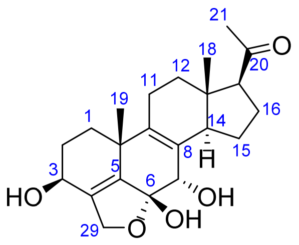

Nodulisporiviridin P (14)

position	δ_C, type	δ_H (J in Hz)[a]	^{1}H–^{1}H COSY	HMBC	ROESY
1	35.2, CH_2	a: 1.68	1b, 2a, 2b	2, 3, 5, 10	19
		b: 1.35	1a, 2a, 2b	3, 10, 19	3
2	30.0, CH_2	a: 1.91	1a, 1b, 2b, 3	1, 4	3-OH
		b: 1.58	1a, 1b, 2a, 3	1, 3	3-OH
3	64.3, CH	4.09	2a, 2b, 3-OH	4, 5	1b
4	135.7, C				
5	136.1, C				
6	108.5, C				
7	72.2, CH	3.64, d (3.9)	7-OH	5, 6, 8, 9	6-OH
8	129.4, C				
9	135.0, C				
10	36.3, C				
11	21.4, CH_2	2.12	12a, 12b	8, 9	18, 19
12	34.6, CH_2	a: 1.99	11, 12b	9, 11	18
		b: 1.61	11, 12a	11, 13, 18	14
13	42.7, C				
14	49.9, CH	2.30	15a, 15b	8, 15	12b, 17
15	23.1, CH_2	a: 1.77	14, 15b, 16a, 16b	13	
		b: 1.30	14, 15a, 16a, 16b	14, 16	18, 21
16	22.3, CH_2	a: 2.10	15a, 15b, 16b, 17	13,17	
		b: 1.63	15a, 15b, 16a, 17	13	
17	61.2, CH	2.66	16a, 16b	12, 13, 16, 18, 20	14
18	12.4, CH_3	0.48, s		12, 13, 14, 17	11, 12a, 15b, 19, 21
19	22.9, CH_3	1.18, s		1, 5, 9, 10	1a, 11, 18, 6-OH
20	208.4, C				
21	31.1, CH_3	2.08, s		17, 20	15b, 18
29	72.7, CH_2	a: 4.64, d (12.0)	29b	4, 5	
		b: 4.23, d (12.0)	29a	4, 5, 6	
3-OH		4.86, d (4.8)	3		2a, 2b
6-OH		5.83, br s		5, 6, 7	7, 19
7-OH		4.23, br s	7		

[a] The indiscernible signals due to overlap or complex multiplicity are reported without designating multiplicity.

Supplementary Table 17. NMR data for 15 (400 MHz for ^{1}H and 100 MHz for ^{13}C, in DMSO-d_6)

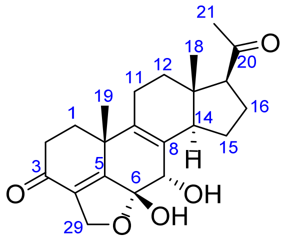

Nodulisporiviridin Q (15)

position	δ_C, type	δ_H (J in Hz)[a]	^{1}H–^{1}H COSY	HMBC	ROESY
1	36.1, CH_2	a: 2.06	1b, 2a, 2b	2, 3, 5, 10	
		b: 1.82	1a, 2a, 2b	2, 10	
2	35.0, CH_2	a: 2.65	1a, 1b, 2b	1, 3, 10	
		b: 2.27	1a, 1b, 2a	3, 4, 10	
3	194.9, C				
4	131.0, C				
5	161.0, C				
6	108.6, C				
7	72.0, CH	3.76, br s	7-OH	5, 6, 8, 9, 14	15a, 15b, 6-OH
8	130.3, C				
9	133.4, C				
10	37.5, C				
11	21.3, CH_2	2.16	12a, 12b	8, 9, 12, 13	18, 19
12	34.5, CH_2	a: 2.03	11, 12b	9, 11	
		b: 1.66	11, 12a	11, 13, 18	
13	42.7, C				
14	49.8, CH	2.37	15a, 15b	8, 9, 13, 15	17
15	23.1, CH_2	a: 1.78	14, 15b, 16a, 16b	13, 14, 17	7
		b: 1.36	14, 15a, 16a, 16b	14	7
16	22.3, CH_2	a: 2.10	15a, 15b, 16b, 17	13, 14, 17	
		b: 1.65	15a, 15b, 16a, 17	13	
17	61.1, CH	2.69	16a, 16b	12, 13, 16, 18, 20	14
18	12.5, CH_3	0.51, s		12, 13, 14, 17	11, 19, 21
19	20.7, CH_3	1.34, s		1, 5, 9, 10	11, 18, 6-OH
20	208.4, C				
21	31.1, CH_3	2.09, s		17, 20	18
29	70.0, CH_2	a: 4.67, d (12.2)	29b	4, 5, 7	
		b: 4.43, d (12.2)	29a	4, 5, 6	
6-OH		6.45, br s		6	7, 19
7-OH		4.71	7		

[a] The indiscernible signals due to overlap or complex multiplicity are reported without designating multiplicity.

Supplementary Notes

Supplementary Note 1. Structural characterization of 1

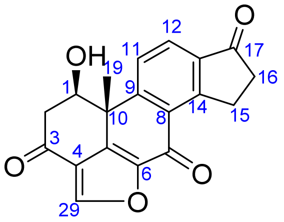

Demethoxyviridin (1)

Compound **1** was obtained as a white amorphous powder and its molecular formula was determined to be $C_{19}H_{14}O_5$ (13 degrees of unsaturation) according to a quasi-molecular ion at *m/z* 323.0923 $[M + H]^+$ in its HRESIMS spectrum. ^{1}H NMR (600 MHz, DMSO-d_6) δ_H 8.79 (H, s), 8.61 (H, d, 8.2), 7.88 (H, d, 8.2), 6.15 (H, br d, 5.8), 4.38 (H, br dt, 10.8, 5.3), 3.62 (H, ddd, 19.1, 7.4, 4.0), 3.52 (H, ddd, 19.1, 7.4, 4.0), 3.08 (H, dd, 18.1, 10.8), 2.74 (H, dd, 18.1, 5.3), 2.64 (2H, m), 1.57 (3H, s); ^{13}C NMR (150 MHz, DMSO-d_6) δ_C 205.7, 190.1, 172.7, 157.1, 156.4, 149.5, 145.7, 144.5, 136.4, 129.6, 128.5, 126.3, 123.0, 70.9, 46.6, 41.6, 35.8, 28.2, 25.3. The NMR data were in good agreement with those of demethoxyviridin[4].

Supplementary Note 2. Structural characterization of 2

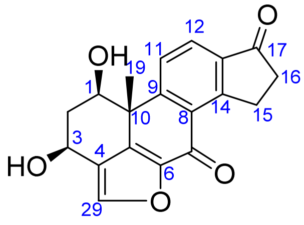

Demethoxyviridiol (2)

Compound **2** was obtained as a white amorphous powder and its molecular formula was determined to be $C_{19}H_{16}O_5$ (12 degrees of unsaturation) according to a quasi-molecular ion at *m/z* 325.1071 $[M + H]^+$ in its HRESIMS spectrum. The ^{13}C NMR spectrum of **2** showed 19 carbons. Combined with the DEPT-135 experiment, these carbons could be categorized into two carbonyls, ten aromatic or olefinic carbons (including three sp^2 methine carbons), one sp^3 quaternary carbon, two oxygenated sp^3 methine carbons, three sp^3 methylene carbons, and one methyl carbon. In the ^1H NMR spectrum of **2**, the characteristic signals of one methyl protons [δ_H 1.51 (3H, s)] and three olefinic or aromatic protons [δ_H 7.83 (H, d, 8.2), 8.05 (H, br s), 8.60 (H, d, 8.2)] were observed. The ^1H NMR and ^{13}C NMR data were similar to those of demethoxyviridin (**1**), which suggested the structural similarities for the two compounds. Compared with **1**, **2** had an oxygenated sp^3 methine carbon (δ_C 59.7) instead of a carbonyl (δ_C 190.1, C-3), which indicated that **2** was the carbonyl reduction derivative of **1** at C-3. This deduction was confirmed by the analysis of ^1H–^1H COSY and HMBC correlations (**Supplementary Figure 11**), and the assignments of all proton and carbon resonances are shown in **Supplementary Table 6**. The ROESY correlations between H_3-19 and Hb-2/1-OH, and between H-1 and H-3 demonstrated that C-19, Hb-2, and 1-OH were on the same face of ring A, while H-1 and H-3 were on the other face of ring A. On the basis of the above analysis, the relative configuration of **2** was established as shown in **Supplementary Figure 12**. Considering that **2** was biosynthesized from the precursor **1** (**Figure 3**), the absolute configuration of C-10 in **2** was assigned as *R*. Thus, the absolute configuration of **2** was assigned as 1*R*, 3*S*, 10*R*. Therefore, **2** was demethoxyviridiol[5].

Supplementary Note 3. Structural characterization of 3

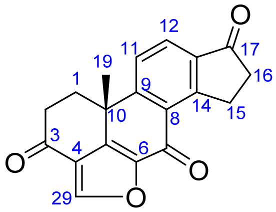

Dehydroxydemethoxyviridin (3)

Compound **3** was obtained as a white amorphous powder with the molecular formula $C_{19}H_{14}O_4$ (13 degrees of unsaturation) as determined by the quasi-molecular ion at *m/z* 307.0966 $[M + H]^+$ in the HRESIMS. The ^{13}C NMR spectrum of **3** showed 19 carbons. Combined with the DEPT-135 experiment, these carbons could be categorized into three carbonyls, ten aromatic or olefinic carbons (including three sp^2 methine carbons), one sp^3 quaternary carbon, four sp^3 methylene carbons, and one methyl carbon. In the ^1H NMR spectrum of **3**, the characteristic signals of one methyl protons [δ_H 1.62 (3H, s)] and three olefinic or aromatic protons [δ_H 7.91 (H, d, 7.9), 7.93 (H, d, 7.9), 8.80 (H, s)] were observed. The ^1H NMR and ^{13}C NMR data were similar to those of demethoxyviridin (**1**), except that an oxygenated methine at δ_C 70.9 (C-1) in **1** was replaced by an sp^3 methylene at δ_C 32.9 (C-1) in **3**, which indicted **3** is the corresponding 1-dehydroxyl product of **1**. Detailed analyses of ^1H–^1H COSY and HMBC data of **3** confirmed above deduction (**Supplementary Figure 11**), and the assignments of all proton and carbon resonances are shown in **Supplementary Table 7**. In the ECD experiment, **3** and **1** had the similar ECD spectra, which suggested that **3** shared an identical configuration at C-10 with **1** (**Supplementary Figure 13a**). Therefore, the absolute configuration of **3** was deduced to be 10*R*. On the basis of the above analysis, **3** was dehydroxydemethoxyviridin[6].

Supplementary Note 4. Structural characterization of 4

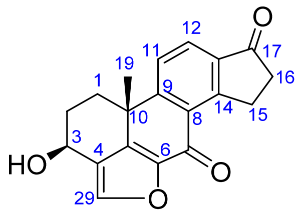

Nodulisporiviridin I (4)

Compound **4** was obtained as a white amorphous powder with the molecular formula $C_{19}H_{16}O_4$ (12 degrees of unsaturation) as determined by the quasi-molecular ion at *m/z* 309.1129 $[M + H]^+$ in the HRESIMS, which was a 2 Da larger molecular weight than that of **3**. Except that a carbonyl (δ_C 191.7, C-3) was replaced by an oxygenated sp^3 methine carbon (δ_C 60.8, C-3), the ^1H NMR and ^{13}C NMR data were similar to those of **3**, which indicated that **4** was the carbonyl reduction derivative of **3** at C-3. This deduction was confirmed by the analysis of ^1H–^1H COSY and HMBC correlations (**Supplementary Figure 11**), and the assignments of all proton and carbon resonances are shown in **Supplementary Table 8**. The ROESY correlations between H_3-19 and Ha-1/Hb-2, and between Hb-1 and H-3 demonstrated that C-19, Hb-2, and Ha-1 were on the same face of ring A, while Hb-1 and H-3 were on the other face of ring A. Based on above analysis, the relative configuration of **4** was established as shown in **Supplementary Figure 12**. Due to the steroids' biosynthesis and the ECD of **4** similar to that of **2** (**Supplementary Figure 13b**), the absolute configuration of **4** was deduced to be 3*S*, 10*R*, and the structure of **4** was elucidated as (3*S*,11b*R*)-3-hydroxy-11b-methyl-1,2,3,7,8,11b-hexahydrocyclopenta[7,8]phenanthro[10,1-*bc*]furan-6,9-dione and named nodulisporiviridin I.

Supplementary Note 5. Structural characterization of 5

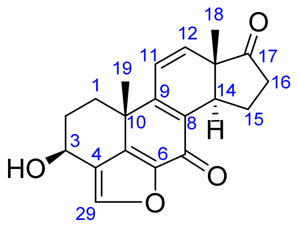

Nodulisporiviridin J (5)

Compound **5** was obtained as a colorless plate-like crystal. The molecular formula was determined to be $C_{20}H_{20}O_4$ (11 degrees of unsaturation) according to a quasi-molecular ion at *m/z* 325.1446 $[M + H]^+$ in its HRESIMS spectrum. The ^{13}C NMR spectrum of **5** showed 20 carbons. Combined with the DEPT-135 experiment, these carbons could be categorized into two carbonyls, eight aromatic or olefinic carbons (including three sp^2 methine carbons), two sp^3 quaternary carbons, two sp^3 methine carbons (including one oxygenated methine carbon), four sp^3 methylene carbons, and two methyl carbons. In the ^1H NMR spectrum of **5**, the characteristic signals of two methyl protons [δ_H 0.89 (3H, s), 1.53 (3H, s)] and three olefinic or aromatic protons [δ_H 6.46 (H, d, 8.1), 6.68 (H, d, 8.1), 7.80 (H, br s)] were observed. All proton resonances were associated with the directly attached carbon atoms in the HSQC experiment. Analysis of the ^1H–^1H COSY experiment indicated the presence of three isolated spin systems (C-1–C-2–C-3, C-11–C-12, and C-14–C-15–C-16). Combined with the ^1H–^1H COSY data, molecular formula, degrees of unsaturation, and the HMBC correlations (**Supplementary Figure 11**), the planar structure of **5** was established, and the assignments of all proton and carbon resonances are shown in **Supplementary Table 9**. The single-crystal X-ray crystallographic analysis of **5** confirmed the above elucidation (**Supplementary Note 18**). The values of the Flack parameter (0.01 (16)) and the Hooft parameter (0.14 (3)) enabled the absolute configuration of **5** to be assigned as 3*S*, 10*R*, 13*S*, 14*R*. Therefore, the structure of **5** was elucidated (3*S*,6b*R*,9a*S*,11b*R*)-3-hydroxy-9a,11b-dimethyl-1,2,3,6b,7,8,9a,11b-octahydrocyclopenta[7,8]phenanthro[10,1-*bc*]furan-6,9-dione and named nodulisporiviridin J.

Supplementary Note 6. Structural characterization of 8

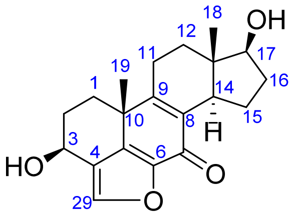

Nodulisporiviridin M (8)

Compound **8** was obtained as a colorless plate-like crystal with the molecular formula $C_{20}H_{24}O_4$ (9 degrees of unsaturation) as determined by the quasi-molecular ion at *m/z* 329.1753 $[M + H]^+$ in the HRESIMS. The ^{13}C NMR spectrum of **8** showed 20 carbons. Combined with the DEPT-135 experiment, these carbons could be categorized into one carbonyl, six aromatic or olefinic carbons (including one sp^2 methine carbon), two sp^3 quaternary carbons, three sp^3 methine carbons (including two oxygenated methine carbons), six sp^3 methylene carbons, and two methyl carbons. In the ^1H NMR spectrum of **8**, the characteristic signals of two methyl protons [δ_H 0.64 (3H, s), 1.49 (3H, s)] and one olefinic or aromatic proton [δ_H 7.84 (H, br s)] were observed. On the basis of the ^1H–^1H COSY data, molecular formula, degrees of unsaturation, and the HMBC correlations (**Supplementary Figure 11**), the planar structure of **8** was established, and the assignments of all proton and carbon resonances are shown in **Supplementary Table 12**. The single-crystal X-ray crystallographic analysis of **8** confirmed the above deduction (**Supplementary Note 19**). The values of the Flack parameter (0.04 (6)) and the Hooft parameter (0.07 (5)) allowed that the assignment of the absolute configuration of **8** was 3*S*, 10*R*, 13*S*, 14*R*, 17*S*. Furthermore, the observed ROESY correlations (**Supplementary Table 12** and **Supplementary Figure 12**) between Ha-1 and H_3-19, between Hb-1 and H-3, between H_2-11 and H_3-19, between Ha-12 and H_3-18, between Hb-12 and H-14, between H-14 and H-17, and between H_3-18 and H_3-19 were consistent with the deduced configuration from X-ray crystallographic analysis. Therefore, the structure of **8** was elucidated as (3*S*,6b*R*,9*S*,9a*S*,11b*R*)-3,9-dihydroxy-9a,11b-dimethyl-2,3,6b,7,8,9,9a,10,11,11b-decahydrocyclopenta [7,8]phenanthro[10,1-*bc*]furan-6(1*H*)-one and named nodulisporiviridin M.

Supplementary Note 7. Structural characterization of 7

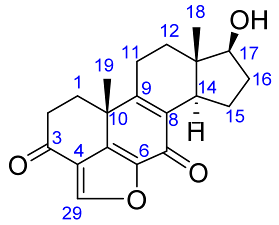

Nodulisporiviridin L (7)

Compound **7** was purified as a white amorphous powder with the molecular formula $C_{20}H_{22}O_4$ (10 degrees of unsaturation) as determined by the quasi-molecular ion at *m/z* 327.1602 $[M + H]^+$ in the HRESIMS. The ^{13}C NMR spectrum of **7** showed 20 carbons. Combined with the DEPT-135 experiment, these carbons could be categorized into two carbonyls, six aromatic or olefinic carbons (including one sp^2 methine carbon), two sp^3 quaternary carbons, two sp^3 methine carbons (including one oxygenated methine carbon), six sp^3 methylene carbons, and two methyl carbons. In the ^1H NMR spectrum of **7**, the characteristic signals of two methyl protons [δ_H 0.65 (3H, s), 1.54 (3H, s)] and one olefinic or aromatic proton [δ_H 8.60 (H, s)] were observed. Except that an oxygenated sp^3 methine carbon (δ_C 60.6, C-3) was replaced by a carbonyl (δ_C 191.9, C-3), the ^1H NMR and ^{13}C NMR data of **7** were similar to those of **8,** which indicated that **7** was the carbonyl derivative of **8** at the C-3 position. Detailed analyses of ^1H–^1H COSY and HMBC data of **7** confirmed above deduction (**Supplementary Figure 11**), and the assignments of all proton and carbon resonances are shown in **Supplementary Table 11**. Combined with the similar ^{13}C NMR data of C-11–C-17 to those of **8**, the observed ROESY correlations (**Supplementary Table 11** and **Supplementary Figure 12**) between Ha-12 and H_3-18, between Hb-12 and H-14, between H-14 and H-17, and between H_3-18 and H_3-19 were consistent with those of **8**, which indicated that the relative configurations of C-10, C-13, C-14, and C-17 should be the same as those of **8**, thus, the relative configuration of **7** was established as shown in **Supplementary Figure 12**. Considering that **7** can be biosynthesized from **8** (**Figure 3**) and the ECD of **7** similar to that of **8** (**Supplementary Figure 13c**), the absolute configuration of **7** was deduced to be 10*R*, 13*S*, 14*R*, 17*S*, and the structure of **7** was elucidated as (6b*R*,9*S*,9a*S*,11b*R*)-9-hydroxy-9a,11b-dimethyl-1,2,6b,7,8,9,9a,10,11,11b-decahydrocyclopenta[7,8]phenanthro[10,1-*bc*]furan-3,6-dione and named nodulisporiviridin L.

Supplementary Note 8. Structural characterization of 6

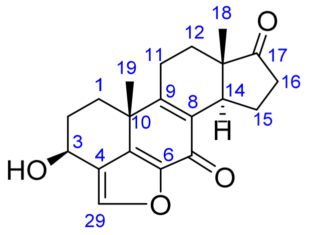

Nodulisporiviridin K (6)

Compound **6** was obtained as a yellowish amorphous powder and its molecular formula was determined to be $C_{20}H_{22}O_4$ (10 degrees of unsaturation) according to a quasi-molecular ion at *m/z* 327.1593 $[M + H]^+$ in its HRESIMS spectrum. The ^{13}C NMR spectrum of **6** showed 20 carbons. Combined with the DEPT-135 experiment, these carbons could be categorized into two carbonyls, six aromatic or olefinic carbons (including one sp^2 methine carbon), two sp^3 quaternary carbons, two sp^3 methine carbons (including one oxygenated methine carbon), six sp^3 methylene carbons, and two methyl carbons. In the ^1H NMR spectrum of **6**, the characteristic signals of two methyl protons [δ_H 0.79 (3H, s), 1.51 (3H, s)] and one olefinic or aromatic proton [δ_H 7.89 (H, d, 1.1)] were observed. Except that an oxygenated sp^3 methine carbon (δ_C 77.6) was replaced by a carbonyl (δ_C 218.3, C-17), the ^1H NMR and ^{13}C NMR data of **6** were similar to those of **8,** which indicated that **6** was the carbonyl derivative of **8** at the C-17 position. Further detailed NMR analyses involving ^1H–^1H COSY and HMBC correlations conformed the above deduction (**Supplementary Figure 11**). And the assignments of all proton and carbon resonances are shown in **Supplementary Table 10**. Combined with the similar ^{13}C NMR data of C-1–C-10 to those of **8**, the observed NOESY correlations (**Supplementary Table 10** and **Supplementary Figure 12**) between Ha-1 and H_3-19, between Hb-1 and H-3, between H_2-11 and H_3-19, between Ha-12 and H_3-18, and between H_3-18 and H_3-19 were consistent with those of **8**, which indicated that the relative configurations of C-3, C-10, and C-13 should be the same as those of **8**. Considering that **6** was biosynthesized from **8** (**Figure 3**), the relative configuration of **6** was established as shown in **Supplementary Figure 12**. Due to the relationship between **6** and **8** in biosynthesis and the ECD of **6** similar to that of **8** (**Supplementary Figure 13c**), the absolute configuration of **6** was deduced to be 3*S*, 10*R*, 13*S*, 14*R*. Thus, the structure of **6** was elucidated as (3*S*,6b*R*,9a*S*,11b*R*)-3-hydroxy-9a,11b-dimethyl-1,2,3,6b,7,8,9a,10,11,11b-decahydrocyclopenta[7,8]p

h-enanthro[10,1-*bc*]furan-6,9-dione and named nodulisporiviridin K.

Supplementary Note 9. Structural characterization of 9

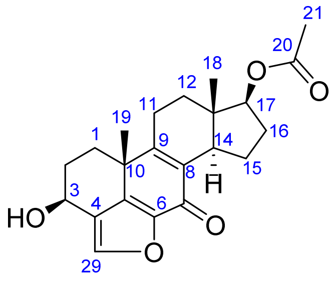

Nodulisporiviridin N (9)

Compound **9** was obtained as a white amorphous powder with the molecular formula $C_{22}H_{26}O_5$ (10 degrees of unsaturation) as determined by the quasi-molecular ion at *m/z* 371.1865 $[M + H]^+$ in the HRESIMS. The ^{1}H and ^{13}CNMR spectroscopic data of **9** showed similar to those of **8** expect for an additional acetyl group (δ_C 20.9/δ_H 2.02, δ_C 170.4) and the significant downfield shift of H-17 (from δ_H 3.56 to δ_H 4.65), which indicated that **9** was a 17-hydroxyl acetylated derivative of **8**. The detailed NMR analyses involving ^{1}H–^{1}H COSY and HMBC correlations shown in **Supplementary Figure 11** conformed the above deduction, and the assignments of all proton and carbon resonances are shown in **Supplementary Table 13**. Combined with the similar ^{13}C NMR data of C-1–C-15 to those of **8**, the observed NOESY correlations (**Supplementary Table 13** and **Supplementary Figure 12**) between Ha-1 and H_3-19, between Hb-1 and H-3, between H_2-11 and H_3-19, between Ha-12 and H_3-18, between Hb-12 and H-14, between H-14 and H-17, and between H_3-18 and H_3-19 were consistent with those of **8**, which indicated that the relative configurations of C-3, C-10, C-13, C-14, and C-17 should be the same as those of **8**, therefore, the relative configuration of **9** was established as shown in **Supplementary Figure 12**. Due to the relationship between **9** and **8** in biosynthesis (**Figure 3**) and the ECD of **9** similar to that of **8** (**Supplementary Figure 13c**), the absolute configuration of **9** was deduced to be 3*S*, 10*R*, 13*S*, 14*R*, 17*S*, and the structure of **9** was elucidated as (3*S*,6b*R*,9*S*,9a*S*,11b*R*)-3-hydroxy-9a,11b-dimethyl-6-oxo-1,2,3,6,6b,7,8,9,9a,10,11,11b-dodecahydrocyclopenta[7,8]phenanthro[10,1-*bc*]furan-9-yl acetate and named nodulisporiviridin N.

Supplementary Note 10. Structural characterization of 11

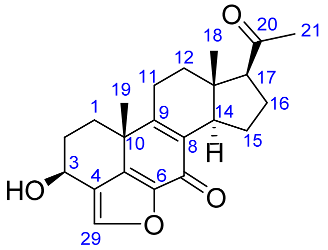

3-Dihydrovirone (11)

Compound **11** was obtained as a yellowish amorphous powder and its molecular formula was determined to be $C_{22}H_{26}O_4$ (10 degrees of unsaturation) according to a quasi-molecular ion at *m/z* 355.1915 $[M + H]^+$ in its HRESIMS spectrum. ^{1}H NMR (600 MHz, $CDCl_3$) δ_H 7.63 (H, s), 4.91 (H, t, 8.1), 2.86 (H, m), 2.53 (2H, m), 2.55 (H, m), 2.40 (H, m), 2.39 (H, m), 2.24 (H, m), 2.19 (H, m), 2.17 (3H, s), 2.07 (H, m), 2.01 (H, dt, 13.4, 3.4), 1.94 (H, m), 1.69 (H, m), 1.65 (H, m), 1.56 (3H, s), 1.55 (H, m), 0.68 (3H, s); ^{13}C NMR (150 MHz, $CDCl_3$) δ_C 209.6, 175.0, 161.5, 145.7, 144.7, 144.7, 134.2, 123.8, 62.3, 60.6, 48.6, 44.0, 38.2, 34.0, 32.1, 31.4, 30.7, 29.4, 24.9, 24.7, 24.5, 12.8. The NMR data were in good agreement with those of 3-dihydrovirone[7].

Supplementary Note 11. Structural characterization of 10

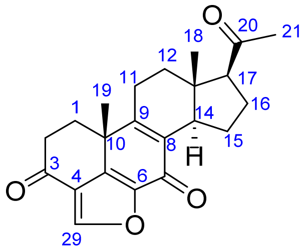

Virone (10)

Compound **10** was obtained as a yellowish amorphous powder. The molecular formula was determined to be $C_{22}H_{24}O_4$ (11 degrees of unsaturation) according to a quasi-molecular ion at *m/z* 353.1758 $[M + H]^+$ in its HRESIMS spectrum. The ^{13}C NMR spectrum of **10** showed 22 carbons. Combined with the DEPT-135 experiment, these carbons could be categorized into three carbonyls, six aromatic or olefinic carbons (including one sp^2 methine carbon), two sp^3 quaternary carbons, two sp^3 methine carbons, six sp^3 methylene carbons, and three methyl carbons. In the ^1H NMR spectrum of **10**, the characteristic signals of three methyl protons [δ_H 0.56 (3H, s), 1.53 (3H, s), 2.13 (3H, s)] and one olefinic or aromatic protons [δ_H 8.62 (H, s)] were observed. All proton resonances were associated with the directly attached carbon atoms in the HSQC experiment. Combined with the ^1H–^1H COSY data, molecular formula, degrees of unsaturation, and HMBC spectra, the planar structure of **10** was established (**Supplementary Figure 11**), and the assignments of all proton and carbon resonances are shown in **Supplementary Table 14**. The observed ROESY correlations (**Supplementary Table 14** and **Supplementary Figure 12**) between H_2-11 andH_3-19, between Hb-12 and H-14, and between H_3-18 and H_3-19 were consistent with those of **8**, which confirmed that the relative configurations of C-10, C-13, and C-14 were the same as those of **8**, furthermore, **10** was biosynthesized from **11** (**Figure 3**), they should have the identical relative configurations of C-10, C-13, C-14, and C-17, so the relative configuration of **10** was established as shown in **Supplementary Figure 12**. Due to the relationship between **10** and **11** in biosynthesis and the ECD of **10** similar to that of **11** (**Supplementary Figure 13c**), the absolute configuration of **10** was deduced to be 10*R*, 13*S*, 14*R*, 17*S*. Thus, **10** was virone[6].

Supplementary Note 12. Structural characterization of 12

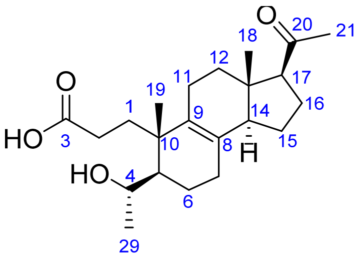

Nodulisporisteroid C (12)

Compound **12** was obtained as a white amorphous powder and its molecular formula was determined to be $C_{22}H_{34}O_4$ (6 degrees of unsaturation) according to a quasi-molecular ion at *m/z* 363.2545 $[M + H]^+$ in its HRESIMS spectrum. ^1H NMR (400 MHz, DMSO-d_6) δ_H 3.77 (H, m), 2.65 (H, br t, 8.6), 2.27 (H, m), 2.26 (H, m), 2.12 (H, m), 2.10 (H, m), 2.07 (3H, s), 2.01 (H, m), 1.98 (H, m), 1.92 (H, m), 1.92 (H, m), 1.85 (H, m), 1.77 (H, m), 1.66 (H, m), 1.65 (H, m), 1.64 (H, m), 1.63 (H, m), 1.61 (H, m), 1.43 (H, br d, 12.0), 1.31 (H, m), 1.22 (H, m), 1.02 (3H, d, 6.1), 0.87 (3H, s), 0.46 (3H, s); ^{13}C NMR (100 MHz, DMSO-d_6) δ_C 208.4, 175.3, 131.5, 131.0, 65.4, 61.3, 51.6, 45.6, 42.9, 39.3, 35.4, 32.4, 31.1, 29.3, 27.2, 23.6, 22.4, 22.0, 21.7, 21.1, 18.7, 12.7. The NMR data were in good agreement with those of nodulisporisteroid C[8].

Supplementary Note 13. Structural characterization of 13

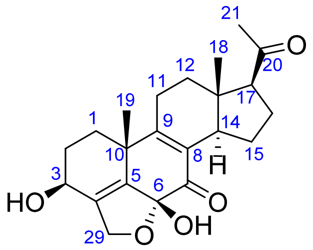

Nodulisporiviridin O (13)

Compound **13** was obtained as a colorless plate-like crystal. The molecular formula was determined to be $C_{22}H_{28}O_5$ (9 degrees of unsaturation) according to a quasi-molecular ion at *m/z* 373.2019 $[M + H]^+$ in its HRESIMS spectrum. The ^{13}C NMR spectrum of **13** showed 22 carbons. Combined with the DEPT-135 experiment, these carbons could be categorized into two carbonyls, four aromatic or olefinic carbons, three sp^3 quaternary carbons (including one oxygenated quaternary carbon), three sp^3 methine carbons (including one oxygenated methine carbon), seven sp^3 methylene carbons (including one oxygenated methylene carbon), and three methyl carbons. In the ^1H NMR spectrum of **13**, the characteristic signals of three methyl protons [δ_H 0.49 (3H, s), 1.39 (3H, s), 2.09 (3H, s)] were observed. All proton resonances were associated with the directly attached carbon atoms in the HSQC experiment. Analysis of the ^1H–^1H COSY experiment indicated the presence of three isolated spin systems (C-1–C-2–C-3–OH, C-11–C-12, and C-14–C-15–C-16–C-17). On the basis of the ^1H–^1H COSY data, molecular formula, degrees of unsaturation, and the HMBC correlations shown in **Supplementary Figure 11**, the planar structure of **13** was established. The single-crystal X-ray crystallographic analysis of **13** confirmed the above deduction (**Supplementary Note 20**). The values of the Flack parameter (0.04 (9)) and Hooft parameter (-0.01 (5)) allowed that the assignment of the absolute configuration of **13** was 3*S*, 6*R*, 10*R*, 13*S*, 14*R*, 17*S*. And the observed ROESY correlations (**Supplementary Table 15** and **Supplementary Figure 12**) between Ha-1 and H_3-19, between Hb-1 and H-3, between H_3-18 and H_2-11/H_3-19/H_3-21, between H-14 and H-17, and between Hb-15 and H_3-18/H_3-21 were consistent with the deduced configuration from X-ray crystallographic analysis. Therefore, the structure of **13** was elucidated as (3*S*,5a*R*,6b*R*,9*S*,9a*S*,11b*R*)-9-acetyl-3,5a-dihydroxy-9a,11b-dimethyl-2,3,4,5a,6b,7,8,9,9a,10,11,11b-dodecahydrocyclopenta[7,8]phenanthro[10,1-*bc*]furan-6(1*H*)-one and named nodulisporiviridin O.

Supplementary Note 14. Structural characterization of 14

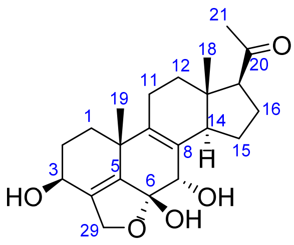

Nodulisporiviridin P (14)

Compound **14** was obtained as a yellowish amorphous powder with the molecular formula $C_{22}H_{30}O_5$ (8 degrees of unsaturation) as determined by the quasi-molecular ion at *m/z* 397.1999 $[M + Na]^+$ in the HRESIMS. The ^{1}H and ^{13}CNMR spectroscopic data of **14** showed similar to **13**, except that a carbonyl (δ_C 192.7, C-7) in **13** was replaced by an oxygenated sp^3 methine carbon (δ_C 72.2) in **14**, which indicated that **14** was the corresponding 7-alcohol. Further detailed NMR analyses involving ^{1}H–^{1}H COSY and HMBC correlations confirmed the above deduction (**Supplementary Figure 11**). According to the analyses of 1D and 2D NMR, the planar structure of **14** was established, and the assignments of all proton and carbon resonances are shown in **Supplementary Table 16**. And the observed ROESY correlations (**Supplementary Table 16** and **Supplementary Figure 12**) between Ha-1 and H_3-19, between Hb-1 and H-3, between H_3-18 and H_2-11/H_3-19/H_3-21, between H-14 and H-17, and between Hb-15 and H_3-18/H_3-21 were consistent with those of **13**, which indicated that the relative configurations of C-3, C-10, C-13, C-14, and C-17 should be the same as those of **13**. Furthermore, the ROESY correlations between 6-OH and H-7/H_3-19 revealed that 6-OH, H-7, and C-19 had identical orientations on ring B, thus, the relative configuration of **14** was established as shown in **Supplementary Figure 12**. Considering that the relationship between **14** and **13** in biosynthesis (**Figure 3**), the absolute configuration of C-10 and C-13 in **14** was assigned as 10*R*, 13*S*. Therefore, the absolute configuration of **14** was deduced to be 3*S*, 6*R*, 7*S*, 10*R*, 13*S*, 14*R*, 17*S*, and the structure of **14** was elucidated as 1-((3*S*,5a*R*,6*S*,6b*R*,9*S*,9a*S*,11b*R*)-3,5a,6-trihydroxy-9a,11b-dimethyl-1,2,3,4,5a,6,6b,7,8,9,9a,10,11,11b-tetradecahydrocyclopenta[7,8]phenanthro[10,1-*bc*]furan-9-yl)ethan-1-one and named nodulisporiviridin P.

Supplementary Note 15. Structural characterization of 15

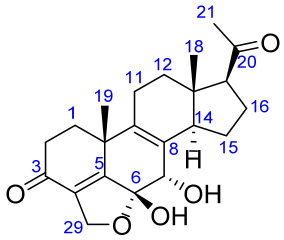

Nodulisporiviridin Q (15)

Compound **15** was obtained as a yellowish amorphous powder with the molecular formula $C_{22}H_{28}O_5$ (9 degrees of unsaturation) as determined by the quasi-molecular ion at *m/z* 395.1821 $[M + Na]^+$ in the HRESIMS. The ^{1}H and ^{13}CNMR spectroscopic data of **15** showed similar to **14**, except that an oxygenated sp^3 methine carbon (δ_C 64.3) in **14** was replaced by carbonyl (δ_C 194.9, C-3) in **15**, which indicated that **15** was the hydroxyl oxidation derivative of **14** at C-3. Further detailed NMR analyses involving ^{1}H–^{1}H COSY and HMBC data confirmed the above deduction (**Supplementary Figure 11**), and the assignments of all proton and carbon resonances are shown in **Supplementary Table 17**. Combined with the similar ^{13}C NMR data of C-11–C-17 to those of **14**, the observed ROESY correlations (**Supplementary Table 17** and **Supplementary Figure 12**) between 6-OH and H-7/H_3-19, between H_3-18 and H_2-11/H_3-19/H_3-21, between H-14 and H-17, which were consistent with those of **14**, indicated that the relative configurations of C-6, C-7, C-10, C-13, C-14, and C-17 should be the same as those of **14**, and the relative configuration of **15** was established as shown in **Supplementary Figure 12**. Due to the relationship between **15** and **14** in biosynthesis (**Figure 3**), the absolute configuration of C-10 and C-13 in **15** was assigned as 10*R*, 13*S*. Therefore, the absolute configuration of **15** was deduced to be 6*R*, 7*S*, 10*R*, 13*S*, 14*R*, 17*S*, and the structure of **15** was elucidated as (5a*R*,6*S*,6b*R*,9*S*,9a*S*,11b*R*)-9-acetyl-5a,6-dihydroxy-9a,11b-dimethyl-1,4,5a,6,6b,7,8,9,9a,10,11,11b-dodecahydrocyclopenta[7,8]phenanthro[10,1-bc]furan-3(2*H*)-one and named nodulisporiviridin Q.

Supplementary Note 16. Structural characterization of 21

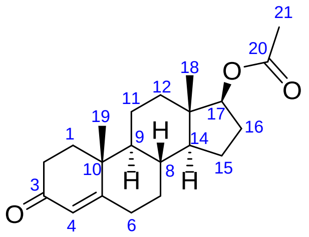

Testosterone acetate (21)

Compound **21** was obtained as a white powder with the molecular formula $C_{21}H_{30}O_3$ (7 degrees of unsaturation) as determined by the quasi-molecular ion at *m/z* 331.2281 $[M + H]^+$ in the HRESIMS. ^1H NMR (400 MHz, $CDCl_3$) δ_H 5.71 (H, s), 4.58 (H, t, 8.1), 2.02 (3H, s), 1.17 (3H, s), 0.82 (3H, s); ^{13}C NMR (100 MHz, $CDCl_3$) δ_C 199.4, 171.1, 170.9, 123.9, 82.4, 53.7, 50.2, 42.4, 38.6, 36.6, 35.6, 35.3, 33.9, 32.7, 31.4, 27.4, 23.4, 21.1, 20.5, 17.3, 12.0. The NMR data were in good agreement with those of testosterone acetate[9].

Supplementary Note 17. Structural characterization of 24

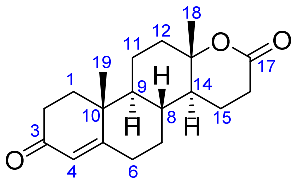

Testolactone (24)

Compound **24** was obtained as a white powder with the molecular formula $C_{19}H_{26}O_3$ (7 degrees of unsaturation) as determined by the quasi-molecular ion at *m/z* 303.1976 $[M + H]^+$ in the HRESIMS. ^1H NMR (400 MHz, $CDCl_3$) δ_H 5.75 (H, s), 2.69 (H, m), 2.59 (H, m), 1.35 (3H, s), 1.16 (3H, s); ^{13}C NMR (100 MHz, $CDCl_3$) δ_C 199.0, 171.0, 169.1, 124.1, 82.6, 52.5, 45.7, 39.0, 38.4, 38.0, 35.5, 33.8, 32.3, 30.4, 28.5, 21.8, 20.0, 19.9, 17.4. The NMR data were in good agreement with those of testolactone[10].

Supplementary Note 18. X-ray crystallographic analysis of 5.

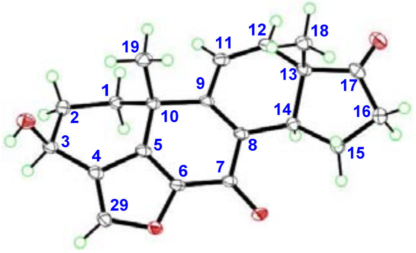

Nodulisporiviridin J (5)

Upon crystallization from CH_3OH using the vapor diffusion method, colorless plate-like crystals of **5** were obtained. Data was collected at 100 K on a Rigaku Oxford Diffraction Supernova Dual Source, Cu at Zero equipped with an AtlasS2 CCD using Cu Kα radiation (λ = 1.54184 Å). Data reduction was carried out with the diffractometer's software[11]. Crystal data: 2 $(C_{20}H_{20}O_5)\cdot H_2O$, M = 666.73, monoclinic, space group $P2_1$; unit cell dimensions were determined to be a = 10.60490(5) Å, b = 7.87989(6) Å, c = 19.68326(9) Å, α = 90.00 °, β = 98.1476(4) °, γ = 90.00 °, V = 1628.238(16) Å^3, Z = 2, Dx = 1.360 g/cm^3, F (000) = 708.0, μ (Cu K_α) = 0.781 mm^{-1}. 57457 reflections were collected (8.422° ≤ θ ≤ 147.376°), in which 6089 independent unique reflections (R_{int} = 0.0688, R_{sigma} = 0.0251) were used in all calculations. Using Olex2[12], the structure was solved by direct methods using the SHELXS program, and refined by the SHELXL program. In the structure refinements, hydrogen atoms were fixed geometrically at the calculated distances and allowed to ride on their parent atoms. The final refinement gave R_1 = 0.0455 [I >2σ(I)], $_wR_2$ = 0.1241 (all data), S = 1.053, Flack = 0.01 (16), and Hooft = 0.14 (3). Crystallographic data for nodulisporiviridin J (**5**) have been deposited in the Cambridge Crystallographic Data Center as supplementary publication no. CCDC 1821761. Copies of the data can be obtained, free of charge, on application to the Director, CCDC, 12 Union Road, Cambridge CB2 1EZ, UK (fax: +44-(0)1223-336033, or e-mail: deposit@ccdc.cam.ac. uk).

Supplementary Note 19. X-ray crystallographic analysis of 8.

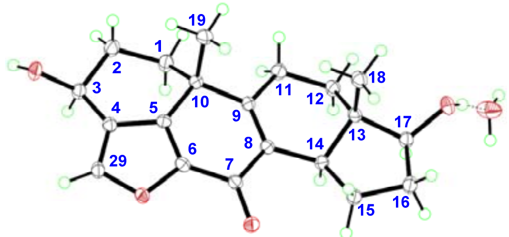

Nodulisporiviridin M (8)

Upon crystallization from CH_3OH using the vapor diffusion method, colorless plate-like crystals of **8** were obtained. Data was collected at 100 K on a Rigaku Oxford Diffraction Supernova Dual Source, Cu at Zero equipped with an AtlasS2 CCD using Cu Kα radiation (λ = 1.54184 Å). Data reduction was carried out with the diffractometer's software[11]. Crystal data: $C_{20}H_{24}O_4 \cdot H_2O$, M = 346.41, orthorhombic, space group $P2_12_12_1$; unit cell dimensions were determined to be a = 6.44547(11) Å, b = 12.89961(16) Å, c = 20.8418(3) Å, α = 90.00 °, β = 90 °, γ = 90.00 °, V = 1732.88(4) Å^3, Z = 4, Dx = 1.328 g/cm^3, F (000) = 744.0, μ (Cu K_α) = 0.770 mm^{-1}. 11583 reflections were collected (8.06° ≤ θ ≤ 147.1°), in which 3437 independent unique reflections (R_{int} = 0.0264, R_{sigma} = 0.0207) were used in all calculations. Using Olex2[12], the structure was solved by direct methods using the SHELXS program, and refined by the SHELXL program. In the structure refinements, hydrogen atoms were fixed geometrically at the calculated distances and allowed to ride on their parent atoms. The final refinement gave R_1 = 0.0301 [I >2σ(I)], $_wR_2$ = 0.0794 (all data), S = 1.067, Flack = 0.04 (6), and Hooft = 0.07 (5). Crystallographic data for nodulisporiviridin M (**8**) have been deposited in the Cambridge Crystallographic Data Center as supplementary publication no. CCDC 1821762. Copies of the data can be obtained, free of charge, on application to the Director, CCDC, 12 Union Road, Cambridge CB2 1EZ, UK (fax: +44-(0)1223-336033, or e-mail: deposit@ccdc.cam.ac. uk).

Supplementary Note 20. X-ray crystallographic analysis of 13.

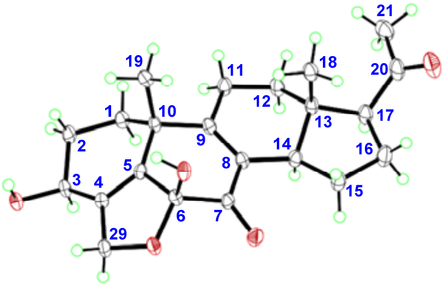

Nodulisporiviridin O (13)

Upon crystallization from CH_3OH using the vapor diffusion method, colorless plate-like crystals of **13** were obtained. Data was collected at 100 K on a Rigaku Oxford Diffraction Supernova Dual Source, Cu at Zero equipped with an AtlasS2 CCD using Cu Kα radiation (λ = 1.54184 Å). Data reduction was carried out with the diffractometer's software[11]. Crystal data: $C_{22}H_{28}O_5$, M = 372.44, monoclinic, space group $C2$; unit cell dimensions were determined to be a = 13.0574(3) Å, b = 8.04518(11) Å, c = 18.9198(4) Å, α = 90.00 °, β = 110.139(2) °, γ = 90.00 °, V = 1865.99(6) Å^3, Z = 4, Dx = 1.326 g/cm^3, F (000) = 800.0, μ (Cu K_α) = 0.754 mm^{-1}. 17476 reflections were collected (9.96° ≤ θ ≤ 148.054°), in which 3560 independent unique reflections (R_{int} = 0.0459, R_{sigma} = 0.0236) were used in all calculations. Using Olex2[12], the structure was solved by direct methods using the SHELXS program, and refined by the SHELXL program. In the structure refinements, hydrogen atoms were fixed geometrically at the calculated distances and allowed to ride on their parent atoms. The final refinement gave R_1 = 0.0337 [I >2σ(I)], $_{W}R_2$ = 0.0885 (all data), S = 1.068, Flack = 0.04 (9), and Hooft = -0.01 (5). Crystallographic data for nodulisporiviridin O (**13**) have been deposited in the Cambridge Crystallographic Data Center as supplementary publication no. CCDC 1821763. Copies of the data can be obtained, free of charge, on application to the Director, CCDC, 12 Union Road, Cambridge CB2 1EZ, UK (fax: +44-(0)1223-336033, or e-mail: deposit@ccdc.cam.ac. uk).

Supplementary Methods

Purification conditions for **1** and **2** from JN1001.

The extract from a 2 L culture of wild-type strain JN1001 was subjected to ODS on MPLC and eluted with an MeOH–H_2O gradient (25:75 to 100:0, v/v). Fraction that contained **1** and **2** was further purified by preparative HPLC (25% aqueous acetonitrile, 3.0 mL min^{-1}) to yield **1** (18.2 mg) and **2** (5.8 mg).

Purification conditions for **3** and **4** from Δ*vidK*-JN1001.

The extract from a 1 L culture of Δ*vidK*-JN1001 strain was subjected to ODS on MPLC and eluted with an MeOH–H_2O gradient (30:70 to 100:0, v/v). Fraction that contained **3** and **4** was further purified by preparative HPLC (40% aqueous methanol, 3.0 mL min^{-1}) to yield **3** (3.5 mg) and **4** (10.5 mg).

Purification conditions for **5–7** from Δ*vidA*-JN1001.

The extract from a 2 L culture of Δ*vidA*-JN1001 strain was subjected to ODS on MPLC and eluted with an MeOH–H_2O gradient (30:70 to 100:0, v/v). Fraction that contained **5–7** was further purified by preparative HPLC (25% aqueous acetonitrile, 3.0 mL min^{-1}) to yield **5** (5.2 mg), **6** (6.8 mg), and **7** (4.1 mg).

Purification conditions for **6** and **8** from Δ*vidD*-JN1001.

The extract from a 1 L culture of Δ*vidD*-JN1001 strain was subjected to ODS on MPLC and eluted with an MeOH–H_2O gradient (25:75 to 100:0, v/v). Fraction that contained **6** and **8** was further purified by preparative HPLC (25% aqueous acetonitrile, 3.0 mL min^{-1}) to yield **6** (6.5 mg) and **8** (4.5 mg).

Purification conditions for **7** and **8** from Δ*vidH*-JN1001.

The extract from a 1 L culture of Δ*vidH*-JN1001 strain was subjected to ODS on MPLC and eluted with an MeOH–H_2O gradient (25:75 to 100:0, v/v). Fraction that contained **7** and **8** was further purified by preparative HPLC (25% aqueous acetonitrile, 3.0 mL min^{-1}) to yield **7** (5.5 mg) and **8** (3.5

mg).

Purification conditions for **9** from Δ*vidP*-JN1001.

The extract from a 1 L culture of Δ*vidP*-JN1001 strain was subjected to ODS on MPLC and eluted with an MeOH–H_2O gradient (35:65 to 100:0, v/v). Fraction that contained **9** was further purified by preparative HPLC (45% aqueous methanol 3.0 mL min^{-1}) to yield **9** (9.5 mg).

Purification conditions for **10**–**12** from Δ*vidF*-JN1001.

The extract from a 3 L culture of Δ*vidF*-JN1001 strain was subjected to ODS on MPLC and eluted with an MeOH–H_2O gradient (35:65 to 100:0, v/v). Fraction that contained **10** and **11** was further purified by preparative HPLC (30% aqueous acetonitrile, 3.0 mL min^{-1}) to yield **10** (6.5 mg) and yield **11** (9.5 mg). Fraction that contained **12** was further purified by preparative HPLC (60% aqueous methanol 3.0 mL min^{-1}) to yield **12** (5.2 mg).

Purification conditions for **13** from Δ*vidS*-JN1001.

The extract from a 1 L culture of Δ*vidS*-JN1001 strain was subjected to ODS on MPLC and eluted with an MeOH–H_2O gradient (30:70 to 100:0, v/v). Fraction that contained **13** was further purified by preparative HPLC (30% aqueous acetonitrile, 3.0 mL min^{-1}) to yield **13** (12.6 mg).

Purification conditions for **14** from Δ*vidQ*-JN1001.

The extract from a 1 L culture of Δ*vidQ*-JN1001 strain was subjected to ODS on MPLC and eluted with an MeOH–H_2O gradient (25:75 to 100:0, v/v). Fraction that contained **14** was further purified by preparative HPLC (25% aqueous acetonitrile, 3.0 mL min^{-1}) to yield **14** (14.2 mg).

Purification conditions for **15**, **14**, and **13** from JA1.

The extract from a 3 L culture of JA1 strain was subjected to ODS on MPLC and eluted with an MeOH–H_2O gradient (25:75 to 100:0, v/v). Fraction that contained **15**, **14**, and **13** was further purified by preparative HPLC (27% aqueous acetonitrile, 3.0 mL min^{-1}) to yield **15** (5.0 mg), **14** (4.1 mg), and **13** (3.2 mg).

Purification conditions for **21** and **24**.

The extract from a 1 L culture of fermentation with progesterone (150 mg dissolved in 2 mL DMSO) by JA4 was subjected to silica-gel column chromatography using a chloroform−methanol gradient (100:0 to 80:20, v/v). Fraction that contained **21** was further purified by preparative HPLC (70% aqueous methanol, 3.0 mL min^{-1}) to yield **21** (23.5 mg). Fraction that contained **24** was further purified by preparative HPLC (50% aqueous methanol 3.0 mL min^{-1}) to yield **24** (14.5 mg).

Supplementary References

1. Zheng, Y. -M. *et al*. Development of a versatile and conventional technique for gene disruption in filamentous fungi based on CRISPR-Cas9 technology. *Sci. Rep.* **7**, 9250 (2017).

2. Fujii, T., Yamaoka, H., Gomi, K., Kitamoto, K. & Kumaga, C. Cloning and nucleotide sequence of the ribonuclease T_1 gene (*rntA*) from *Aspergillus oryzae* and its expression in *Saccharomyces cerevisiae* and *Aspergillus oryzae*. *Biosci. Biotechnol. Biochem.* **59**, 1869-1874 (1995).

3. Jin, F. J., Maruyama, J., Juvvadi, P. R., Arioka, M. & Kitamoto, K. Development of a novel quadruple auxotrophic host transformation system by *argB* gene disruption using *adeA* gene and exploiting adenine auxotrophy in *Aspergillus oryzae*. *FEMS Microbiol. Lett.* **239**, 79-85 (2004).

4. Zheng, Q. -C. *et al*. Nodulisporisteriods A and B, the first 3,4-seco-4-methyl-progesteroids from *Nodulisporium* sp. *Steroids* **78**, 896-901 (2013).

5. Cole, R. J. *et al*. Desmethoxyviridiol, a new toxin from *Nodulisporium hinnuleum*. *Phytochemistry* **14**, 1429-1432 (1975).

6. Blight, M. M. & Grove, J. F. Viridin. part 8. structures of the analogues virone and wortmannolone. *J. Chem. Soc., Perkin Trans. 1*, 1317-1322 (1986).

7. Andersson, P. F., Bengtsson, S., Cleary, M., Stenlid, J. & Broberg, A. Viridin-like steroids from *Hymenoscyphus pseudoalbidus*. *Phytochemistry* **86**, 195-200 (2013).

8. Zhao, Q. *et al.* Nodulisporisteroids C–L, new 4-methyl-progesteroid derivatives from *Nodulisporium* sp. *Steroids* **102**, 101-109 (2015).

9. Hunter, A. C., Watts, K. R., Dedi, C. & Dodd, H. T. An unusual ring–A opening and other reactions in steroid transformation by the thermophilic fungus *Myceliophthora thermophila*. *J.*

Steroid Biochem. Mol. Biol. **116**, 171-177 (2009).

10. Kolek, T., Szpineter, A. & Swizdor, A. Baeyer-Villiger oxidation of DHEA, pregnenolone, and androstenedione by *Penicillium lilacinum* AM111. *Steroids* **73**, 1441-1445 (2008).

11. Agilent Technologies, CrysAlisPRO, Version 1.171.36.28 (2013).

12. Dolomanov, O. V., Bourhis, L. J., Gildea, R. J., Howard, J. A. K. & Puschmann, H. J. *Appl. Cryst.* **42**, 339-341 (2009).
